# Alisol B 23‐Acetate Down‐Regulated GRP94 to Restore Endoplasmic Reticulum Homeostasis on Non‐Alcoholic Steatohepatitis

**DOI:** 10.1002/fsn3.70086

**Published:** 2025-03-05

**Authors:** Fei Qu, Yuming Wang, Yanping Zhang, Feng Chen, Yuanliang Ai, Weibo Wen, Jiabao Liao, Hanzhou Li, Huan Pei, Mingxi Lu, Ling Yang, Ning Wang, Huantian Cui

**Affiliations:** ^1^ Jiaxing Hospital of Traditional Chinese Medicine Jiaxing China; ^2^ Tianjin University of Traditional Chinese Medicine Tianjin China; ^3^ Kunming Municipal Hospital of Traditional Chinese Medicine Kunming China; ^4^ Yunnan University of Chinese Medicine Kunming China; ^5^ Qingdao Agricultural University Qingdao China

**Keywords:** Alisol B 23‐acetate, endoplasmic reticulum stress, ER‐associated degradation, GRP94, non‐alcoholic steatohepatitis, transcriptomics

## Abstract

Non‐alcoholic steatohepatitis (NASH) poses a serious threat to human health. Alisol B 23‐Acetate (AB23A) has shown beneficial effects on NASH, but its mechanism of action remains unclear. We conducted in vitro experiments by inducing L02 cell damage with free fatty acids (FFA) and administering various concentrations of AB23A. We found that AB23A intervention reduced triglyceride (TG) levels in FFA‐induced L02 cells and improved cellular steatosis. Transcriptomic analysis revealed that AB23A intervention significantly downregulated glucose‐regulated protein 94 (Grp94), indicating that AB23A primarily regulates the protein processing pathway in the endoplasmic reticulum. Within this pathway, AB23A intervention also significantly downregulated endoplasmic reticulum stress (ERS)‐related genes (*PERK*, *eIF2α*, *ATF4*) and ER‐associated degradation (ERAD)‐related genes (*FBXO2*, *DERL*, *HSP90AA1*). When we silenced GRP94, the regulatory effects of AB23A on TG levels, cellular steatosis, ERS‐related proteins (p‐PERK/PERK, p‐eIF2α/eIF2α, ATF4), and ERAD‐related proteins (FBXO2, DERL, HSP90α) disappeared. In vivo, AB23A intervention promoted recovery of the liver index in NASH mice, reduced hepatic inflammatory infiltration and lipid deposition, improved serum alanine aminotransferase (ALT) and aspartate aminotransferase (AST) activities, and reduced liver TG levels. RT‐qPCR and Western blot results demonstrated that AB23A intervention dose‐dependently downregulated the gene and protein expression of GRP94 and ERS‐ and ERAD‐related factors. There was no significant difference between the effects of high‐dose AB23A intervention and PPC intervention. This study demonstrated, through both in vitro and in vivo experiments, that AB23A improves hepatic steatosis. This effect may be related to the downregulation of GRP94, which suppresses ERS and ERAD, thereby restoring ER homeostasis.

## Graphical abstract

Alisol B 23‐Acetate (AB23A) alleviated hepatic injury and lipid deposition in MCD diet‐induced non‐alcoholic steatohepatitis (NASH) mice. Alisol B 23‐Acetate reduced liver cell damage by restoring endoplasmic reticulum homeostasis. AB23A down‐regulated GRP94, suppressing ER stress and ER‐associated degradation (ERAD).

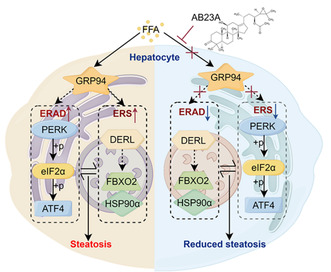

## 1 Introduction

There is an increasing prevalence of worldwide obesity and metabolic syndrome. Non‐alcoholic fatty liver disease (NAFLD) is one of the most common chronic liver diseases globally, with its incidence now over 25% (Wei et al. 2024). NAFLD encompasses multiple stages, ranging from simple fatty liver to non‐alcoholic steatohepatitis (NASH) and can progress to advanced liver fibrosis and hepatocellular carcinoma. NASH, a more severe form of NAFLD, serves as a critical intermediate stage in the progression from NAFLD to liver fibrosis and liver cancer. Approximately 20% of NAFLD patients will develop NASH, and alarmingly, about 20% of NASH patients will progress to liver cirrhosis (Povsic et al. 2019; Sheka et al. 2020). Given the serious threat NASH poses to human health, in‐depth research on the mechanisms underlying NASH development is crucial, especially for the search for effective intervention drugs.

Hepatocytes are rich in endoplasmic reticulum (ER), an essential organelle involved in the synthesis and processing of numerous proteins and lipids. During the development of NASH, abnormal lipid accumulation can disrupt ER function in hepatocytes, leading to the buildup of unfolded or misfolded proteins in the ER. This triggers the cell's defense mechanism, causing ER stress (ERS) and activating the unfolded protein response (UPR). As a result, protein synthesis is blocked, and ER‐associated degradation (ERAD) is induced (Ajoolabady et al. 2023). A randomized controlled trial revealed that severe ER stress exists in the livers of NASH patients, and promoting the restoration of ER homeostasis can effectively improve liver function (Okada et al. 2018). Consequently, restoring ER homeostasis has become a crucial target for controlling NASH progression.

Glucose‐regulated protein 94 (Grp94) is a principal molecular chaperone protein in the ER and a member of the HSP90 family, which is involved in the folding and quality control of various proteins (Xu et al. 2024). It plays a significant role in the development of NASH, with studies indicating its activation in NASH mouse models (Lebeaupin et al. 2018; Li et al. 2020). Notably, The regulation of GRP94 is crucial for maintaining the homeostasis of the ER (Marzec et al. 2012).

Natural products hold great potential in restoring ER homeostasis. Studies have shown that artemisinin (Yin et al. 2021), curcumin (Zhou et al. 2021), and ginsenoside Rb1 (Shaukat et al. 2021) can inhibit ERS‐related proteins, particularly protein kinase‐like ER kinase (PERK) phosphorylation, to alleviate ERS. Alisol B 23‐Acetate (AB23A), an important active ingredient in the traditional Chinese medicine *Alisma orientale* (Sam.) Juzep., has demonstrated beneficial effects on NASH (Li et al. 2024). Furthermore, Zexie decoction, a traditional Chinese medicine formula with *Alisma orientale* (Sam.) Juzep. as its main ingredient, has been widely used in treating NAFLD and effectively protecting liver function (Li et al. 2024). However, the precise mechanism by which AB23A exerts its therapeutic effects on NASH remains to be explored.

In this study, we first conducted in vitro experiments using FFA‐induced L02 cells and administered different concentrations of AB23A to evaluate its effect on improving cellular steatosis. Based on these findings, we hypothesized that AB23A improves NASH by restoring ER homeostasis. To validate this hypothesis, we evaluated the effects of AB23A on ERS‐ and ERAD‐related factors after silencing GRP94. Furthermore, we conducted in vivo experiments to verify the anti‐NASH effects of AB23A.

## 2 Materials and Methods

### 2.1 Materials

The comprehensive information regarding reagents, kits, antibodies, and other materials utilized in this study is provided in Appendix S1.

### 2.2 In Vitro Experiments

#### 2.2.1 Cell Culture

L02 cells were provided by iCell Bioscience Inc. (Shanghai, China). Subsequently, we cultured L02 cells in RPMI‐1640 medium supplemented with 10% fetal bovine serum, 100 μg/mL penicillin, and 100 μg/mL streptomycin. The cells were maintained in a constant temperature incubator with 5% CO_2_ at 37°C. Passaging was performed when the cell density reached 60%–70%.

#### 2.2.2 Cell Transfection

Following cell plating, allow the cells to grow to a confluency rate of 30%–40%. Subsequently, replace the medium with a fresh medium containing Lipo2000 transfection reagent, siGRP94 plasmid, and the empty vector (siNC) for a 6‐h incubation period. The sequence of siNC:siNC: Sense sequence: UUCUCCGAACGUGUCACGUTT, Antisense sequence: ACGUAGCACGUUCGGAGAATT. The sequence of siGRP94: siGRP94‐1: Sense sequence: GAAGAAGCAUCUGAUUACC, Antisense sequence: GGUAAUCAGAUGCUUCUUC; siGRP94‐2: Sense sequence: GGUCAGAGCUGACGAUGAAGU, Antisense sequence: UUCAUCGUCAGCUCUGACCGA. Afterward, replace the medium with RPMI‐1640 medium supplemented with 10% fetal bovine serum and penicillin–streptomycin, and continue culturing for 24 h.

#### 2.2.3 MTT Assay

Following the plating of L02 cells, we treated them with varying concentrations of AB23A (0, 20, 40, 80, and 160 μM) for 24 h. Subsequently, 10 μL of MTT solution (5 mg/mL) was added to each well. After a 4‐h incubation period, the medium was discarded, and 100 μL of DMSO was added. The absorbance of the samples was measured at 490 nm, and the relative cell viability was calculated based on the absorbance values.

#### 2.2.4 Establishment of NASH Cell Model

After the cells were plated, they were allowed to grow until cell confluence reached 80%. Subsequently, the cells were stimulated with 1 mM FFA (palmitic acid: oleic acid at a ratio of 1:2) for 24 h to establish a NASH cell model. The model was evaluated by measuring the TG level in the cells and performing Oil Red O staining on the cells.

#### 2.2.5 Transcriptomic Analysis

After plating, the cells were cultured in an incubator for 24 h and subsequently divided into Control, FFA, and FFA + 80 μM AB23A groups. These groups underwent further culturing for 24 h. Following this, all media were removed, and the cells were washed twice with cold PBS. Total RNA was isolated using TRIzol, and the purity, concentration, and integrity of the RNA samples were assessed to ensure the utilization of qualified samples for transcriptome sequencing. After passing quality control, library construction was performed, and sequencing was conducted on the Illumina platform. DESeq2 software was employed to analyze the differentially expressed genes (DEGs) between the control, FFA, and FFA + 80 μM AB23A groups. Genes with |Log_2_(FoldChange)| ≥ 1 and *p*_adj_ ≤ 0.05 were identified as DEGs, and KEGG analysis was conducted on these DEGs.

### 2.3 In Vivo Experiment

#### 2.3.1 Animals

We obtained sixty healthy male C57BL/6 mice, aged 6–8 weeks, with a body weight of approximately 22 g from Si Pei Fu (Beijing) Biotechnology Co. Ltd. (Production License No.: SYXK (Beijing) 2019–0030). The mice were housed under standard conditions, including a 12‐h light–dark cycle, an ambient temperature of approximately 25°C, and an ambient humidity of approximately 50%. Food and water were provided ad libitum. Approval for all animal experiments was obtained from the Ethical Review Committee of Animal Experiments at Yunnan University of Chinese Medicine (Approval No.: R‐062024G256).

#### 2.3.2 Modeling, Grouping, and Administration

After a week of adaptive feeding, we randomly assigned the 60 C57BL/6 mice into six groups: Control group, NASH group, Polyene Phosphatidylcholine (PPC) intervention group, and three AB23A intervention groups with different doses, namely Low‐dose AB23A (L‐AB23A), Medium‐dose AB23A (M‐AB23A), and High‐dose AB23A (H‐AB23A). With the exception of the Control group, all other five groups received a methionine and choline deficiency (MCD) diet, as established in previous studies (Li et al. 2024). Additionally, the PPC intervention group received 88 mg/kg/d of PPC via oral administration, while the low, medium, and high‐dose AB23A intervention groups received 15, 30, and 60 mg/kg/d of AB23A via oral administration, respectively. The dosage settings for PPC and AB23A were determined based on prior research (Li et al. 2024). The Control and NASH groups were administered an equal volume of saline daily via gastric gavage as a parallel control. The administration of drugs continued for 6 weeks, during which we recorded the mice's body weights weekly. After 6 weeks, we euthanized the mice and collected blood. The serum was separated by centrifugation and stored frozen. We promptly removed and weighed the liver, calculating the liver index. The liver index was calculated using the following formula: liver index (%) = liver weight (g)/body weight (g) × 100. Subsequently, the left lateral lobe from identical locations was fixed for pathological sectioning to observe pathological damage, while the remaining liver lobes were frozen for RT‐qPCR and Western blot analysis.

### 2.4 Biochemical Detection

We employed reagent kits to measure the alanine aminotransferase (ALT) and aspartate aminotransferase (AST) activities in the serum of mice from each group. Furthermore, we collected liver tissues and cells cultured in vitro, normalizing the total protein using the BCA method. The triglyceride (TG) levels in both liver tissues and cultured cells were detected using reagent kits.

### 2.5 Pathological Staining

To assess pathological changes and inflammation in liver tissue, we prepared paraffin sections of liver tissue, stained them with hematoxylin and eosin (HE), and observed them under an optical microscope. For observing the level of lipid deposition in hepatocytes, we prepared frozen sections of liver tissue, stained them with Oil Red O, and examined them under an optical microscope. Quantification of Oil Red O‐positive areas was conducted using ImageJ software.

### 2.6 RT‐qPCR

We extracted total RNA from liver tissue using a total RNA extraction kit and measured the RNA concentration. Subsequently, we obtained cDNA using a reverse transcription kit. We utilized qPCR to determine the target gene mRNA expression levels. The $2^{-\Delta \Delta C_{T}}$ method relative to β‐actin was used to compute the relative expression levels of each target mRNA. Primer sequences are provided in Appendix S1.

### 2.7 Western Blot

We extracted total proteins from liver tissue samples or cells cultured in vitro and determined their concentrations using a BCA kit. Subsequently, we separated the total liver protein using SDS‐PAGE and transferred it to a PVDF membrane. To minimize non‐specific binding, we blocked the PVDF membrane with a 5% skim milk powder solution for 1 h. Following this, we incubated the membrane with target protein antibodies at 4°C for over 12 h to ensure adequate binding between the antibodies and target proteins. After multiple washes, the membrane was incubated with HRP‐labeled secondary antibodies for 1 h at room temperature. Upon completion of the incubation, we washed the membrane multiple times to eliminate excess reagents and unbound antibodies. Finally, we utilized ECL imaging technology to visualize the protein bands on the membrane, and ImageJ software facilitated precise quantitative analysis of the results.

### 2.8 Statistic Analysis

We conducted statistical analysis using the SPSS Pro online data analysis platform. All data were presented as mean ± standard deviation (SD). We applied the Shapiro–Wilk test to evaluate the normality of the data distribution. To assess the significance of differences between groups, the Student's unpaired *t*‐test was utilized, as well as one‐way or two‐way ANOVA, with Tukey's and Bonferroni's post hoc tests for multiple comparisons. If the data did not follow a normal distribution, the rank‐sum test was employed for analysis. For statistical significance, we set a threshold of a *p*‐value of less than 0.05 (*p* < 0.05).

## 3 Results

### 3.1 AB23A Intervention Improved FFA‐Induced Steatosis of L02 Cells

**FIGURE 1:**
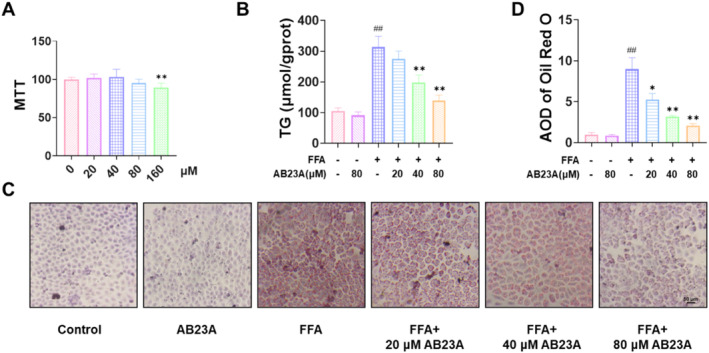
AB23A intervention improves FFA‐induced steatosis of L02 cells. MTT results showing that AB23A at concentrations below 80 μM has no significant effect on the viability of L02 cells (A). (B–D) Effect of AB23A on hepatocyte steatosis in vitro assessed by the TG levels in cells from each group and the level of lipid deposition in cells via Oil Red O staining. (B) AB23A intervention reduces the TG levels in FFA‐induced L02 cells. (C, D) Oil Red O staining results showing that AB23A intervention improves FFA‐induced steatosis in L02 cells. Cell groups: Control, Control+80 μM AB23A, FFA, FFA + 20 μM AB23A, FFA + 40 μM AB23A, and FFA + 80 μM AB23A. Data are presented as the mean ± SD. In (A) *n* = 6. **p < 0.01 compared to the 0 μM group. In (B–D) *n* = 3. ^#^ *p* < 0.05, ^##^ *p* < 0.01 compared to the Control group; *p < 0.05, **p < 0.01 compared to the FFA group. One‐way or two‐way ANOVA followed by post hoc analysis with Tukey's test for comparison between more groups.

We initiated our investigation by conducting an MTT assay to assess the cytotoxicity of AB23A on L02 cells. The outcomes revealed that AB23A concentrations below 80 μM had no significant impact on the viability of L02 cells (Figure 1A). Consequently, we opted for 20, 40, and 80 μM AB23A for subsequent analysis. We categorized the cells into several groups: Control, Control + 80 μM AB23A, FFA, FFA + 20 μM AB23A, FFA + 40 μM AB23A, and FFA + 80 μM AB23A. By evaluating the TG levels of cells in each group and observing the level of lipid deposition in cells through Oil Red O staining, we assessed the impact of AB23A on hepatocyte steatosis in vitro. The findings illustrated that FFA‐induced L02 cells exhibited elevated TG levels and severe steatosis. AB23A intervention mitigated TG levels and ameliorated cell steatosis, with 80 μM AB23A demonstrating the most pronounced effect (Figure 1B–D). Thus, we selected the 80 μM AB23A intervention group for further analysis.

### 3.2 AB23A Intervention Down‐Regulated *GRP94* Expression

**FIGURE 2:**
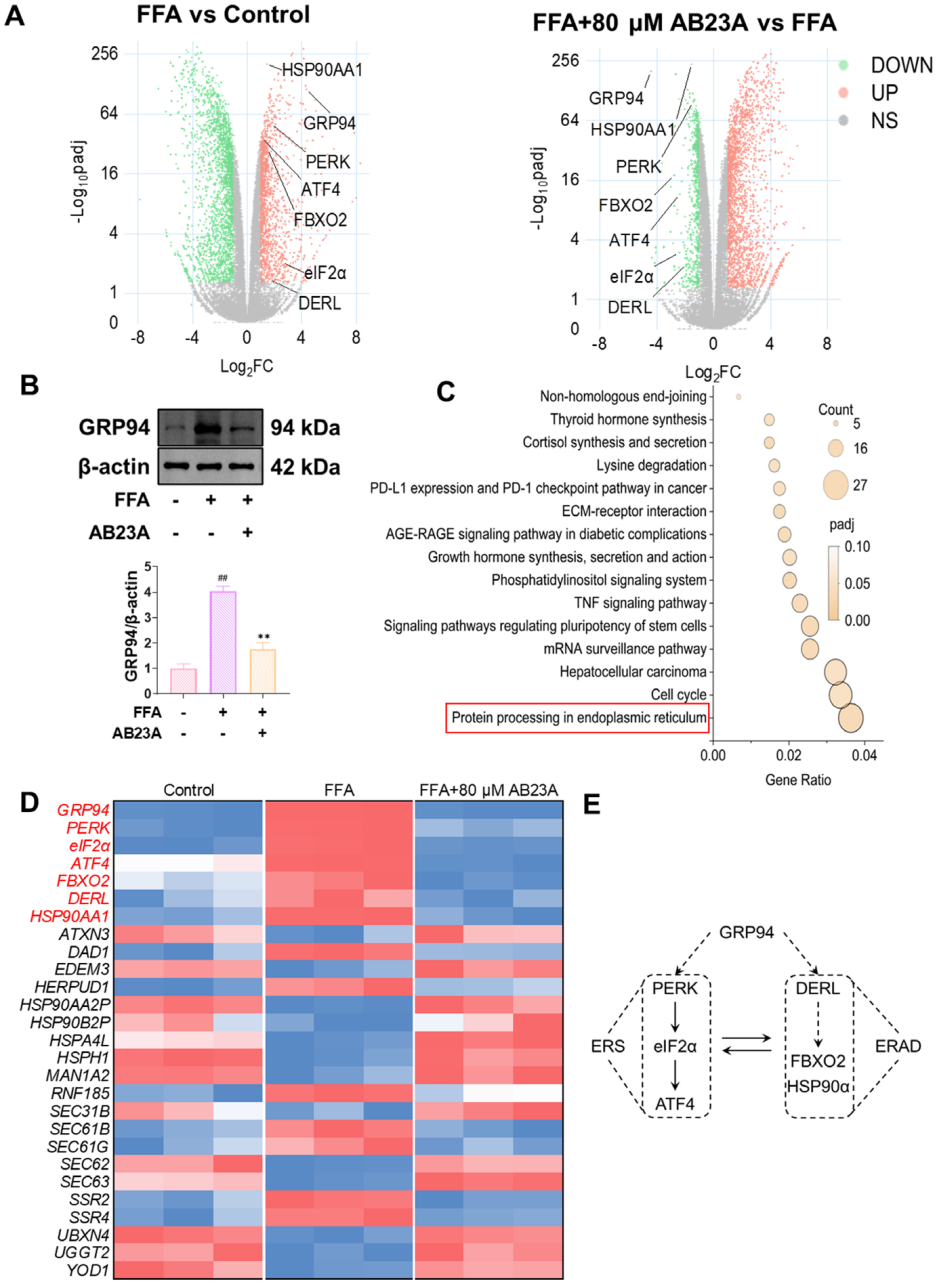
AB23A intervention down‐regulates GRP94 expression. Cells were divided into Control, FFA, and FFA + 80 μM AB23A groups and transcriptomic analysis was performed. Using the criteria of |Log_2_(FoldChange)| ≥ 1 and padj ≤ 0.05, DEGs were screened between the FFA group vs. Control group and the FFA + 80 μM AB23A group vs. FFA group. (A, B) DEGs visualized using volcano plots. We found that AB23A intervention significantly downregulates GRP94 expression (A), which was also confirmed by western blot. (C–E) KEGG pathway enrichment analysis revealing that GRP94 was enriched in the “Protein processing in endoplasmic reticulum” pathway (C). In this pathway, ERS‐related genes (*PERK*, *eIF2α*, *ATF4*) and ERAD‐related genes (*FBXO2*, *DERL*, *HSP90AA1*) are highlighted in the heatmap (D), and the relationship between GRP94, ERS, and ERAD is simplified and presented (E). Data are presented as the mean ± SD. *n* = 3 per group. ^##^ *p* < 0.01 compared to the Control group; **p < 0.01 compared to the FFA group. One‐way or two‐way ANOVA followed by post hoc analysis with Bonferroni test for comparison between more groups.

We organized the cells into three groups: Control group, FFA group, and FFA + 80 μM AB23A group, and conducted transcriptome analysis. DEGs were identified between the FFA group vs. Control group and the FFA + 80 μM AB23A group vs. FFA group, based on the criteria of |Log_2_(FoldChange)| ≥ 1 and *p*_adj_ ≤ 0.05. Detailed information regarding the DEGs is provided in Appendix S1. Our findings revealed that compared to the Control group, GRP94 exhibited upregulation in the FFA group, whereas after AB23A intervention, the downregulation of GRP94 was most pronounced (Figure 2A). Western blot results demonstrated that relative to the Control group, the protein expression of GRP94 increased in the FFA group, whereas after AB23A intervention, a significant reduction in GRP94 protein expression was observed (Figure 2B). Subsequently, KEGG pathway enrichment analysis was performed, revealing enrichment of GRP94 in the “Protein processing in endoplasmic reticulum” pathway. Consequently, our subsequent analysis focused on the effects of AB23A on this pathway (Figure 2C).

Upon visualization of the heatmap of genes associated with the “Protein processing in endoplasmic reticulum” pathway, we observed that, in addition to significantly downregulating GRP94 expression, AB23A also markedly downregulated ERS‐related genes (*PERK*, *eIF2α*, *ATF4*) and ERAD‐related genes (*FBXO2*, *DERL*, *HSP90AA1*) (Figure 2D). Previous study (Li et al. 2024) have highlighted GRP94 as a pivotal protein promoting ERS and ERAD, and down‐regulating GRP94 can ameliorate ERS and ERAD. We plotted the relationship between GRP94, ERS and ERAD (Figure 2E). Building upon this, we speculate that AB23A may improve FFA‐induced L02 cell steatosis by down‐regulating GRP94, thereby improving ERS and ERAD processes.

### 3.3 AB23A Improves ERS and ERAD by Down‐Regulating GRP94

**FIGURE 3:**
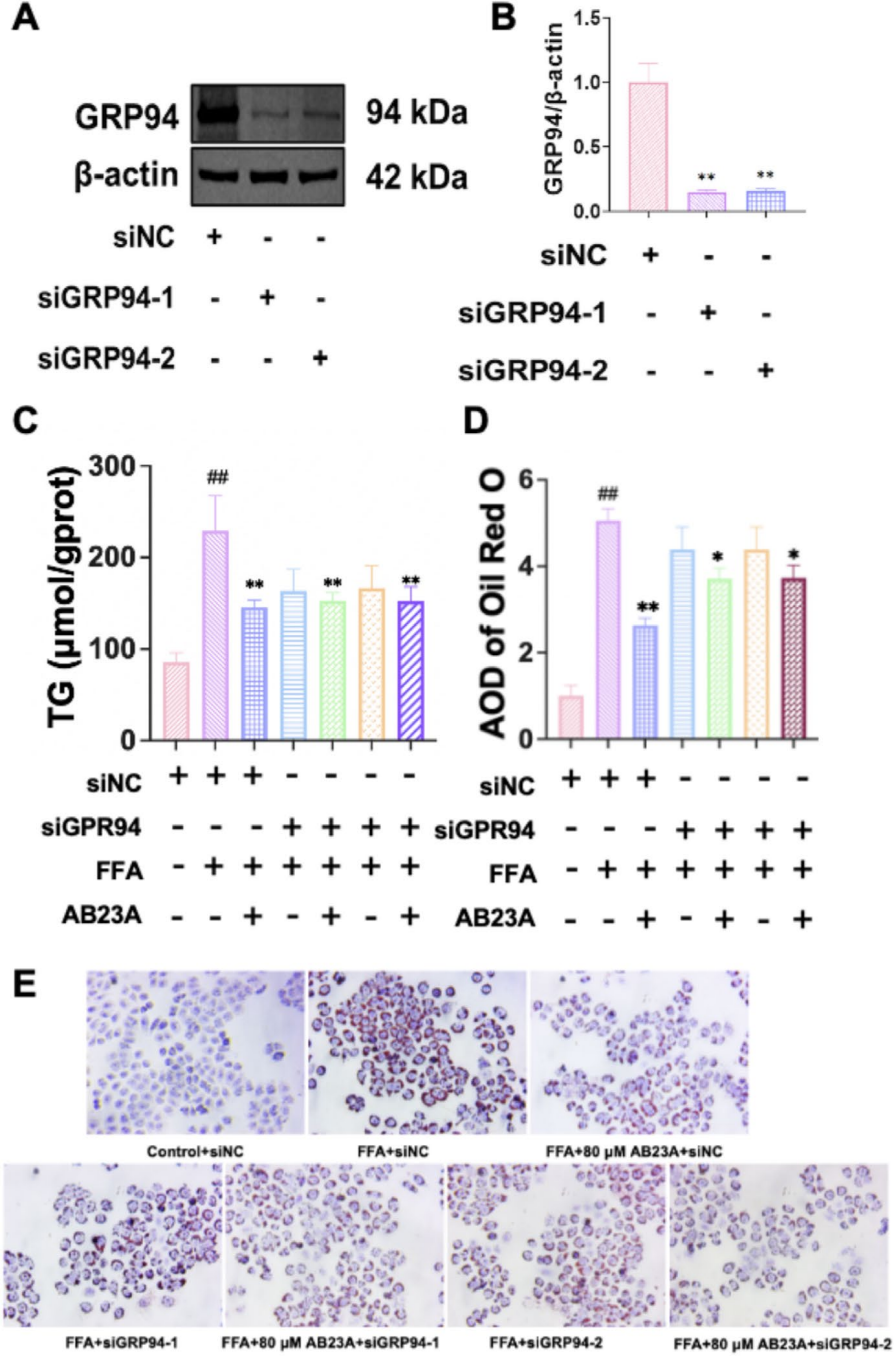
Silencing GRP94 abolishes the improvement effect of AB23A intervention on FFA‐induced steatosis in L02 cells. The effect of AB23A intervention on FFA‐induced steatosis in L02 cells was evaluated by silencing the GRP94 gene. Western blot results demonstrated that both siRNAs significantly suppressed the expression of GRP94 (A, B). **p < 0.01 compared to the siNC group. The results show that after silencing GRP94, the effect of AB23A intervention on reducing the TG levels (C) and improving steatosis (D, E) in FFA‐induced L02 cells disappears. Cell groups: Control+siNC, FFA + siNC, FFA + 80 μM AB23A + siNC, FFA + siGRP94‐1, FFA + siGRP94‐1 + 80 μM AB23A, FFA + siGRP94‐2, and FFA + siGRP94‐2 + 80 μM AB23A. Data are presented as the mean ± SD. *n* = 3 per group. ^##^ *p* < 0.01 compared to the Control+siNC group; *p < 0.05, **p < 0.01 compared to the FFA + siNC group. One‐way or two‐way ANOVA followed by post hoc analysis with Tukey's test for comparison between more groups.

**FIGURE 4:**
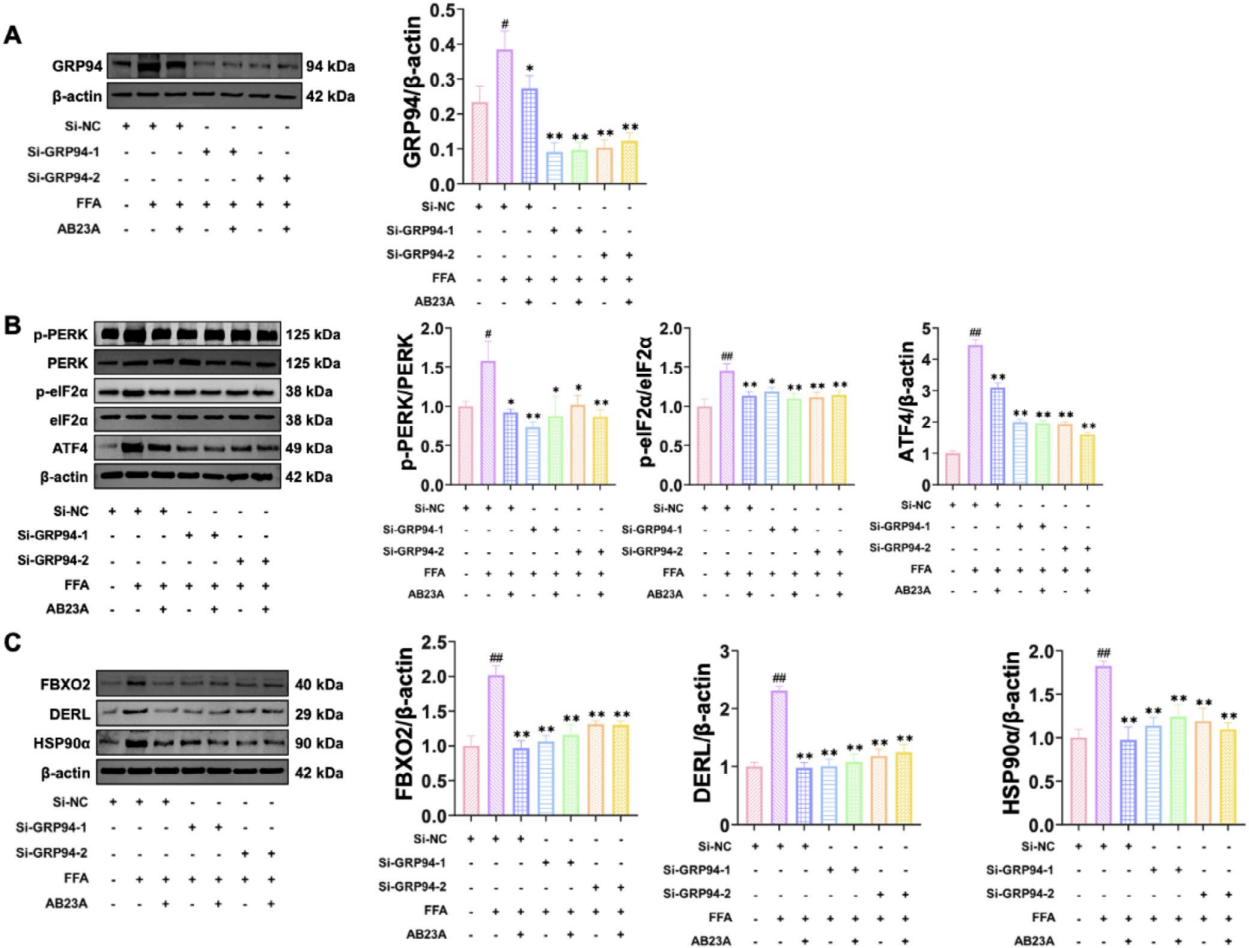
AB23A intervention improves ERS and ERAD via down‐regulated of GRP94. The effect of AB23A intervention on the expression of ERS and ERAD‐related proteins was evaluated by silencing the GRP94 gene. Western blot results demonstrated that both siRNAs significantly suppressed the expression of GRP94 (A) the effect of AB23A on downregulating the expression of ERS‐related proteins (p‐PERK/PERK, p‐eIF2α/eIF2α, ATF4) (B) and ERAD‐related proteins (FBXO2, DERL, HSP90α) (C) abolishes. Data are presented as the mean ± SD. *n* = 3 per group. ^#^ *p* < 0.05, ^##^ *p* < 0.01 compared to the Control+siNC group; *p < 0.05, **p < 0.01 compared to the FFA + siNC group. One‐way or two‐way ANOVA followed by post hoc analysis with Tukey's test for comparison between more groups.

To validate the aforementioned speculation, we proceeded to assess the effects of AB23A on FFA‐induced L02 cell steatosis and the expression of ERS and ERAD‐related proteins following the silencing of the GRP94 gene. We selected two different GRP94 siRNAs to reduce the potential risk of off‐target effects. The results showed that both siRNAs effectively suppressed the expression of GRP94 (Figure 3A). Subsequently, we allocated the cells into the following groups: Control+siNC, FFA + siNC, FFA + 80 μM AB23A + siNC, FFA + siGRP94‐1, FFA + siGRP94‐1 + 80 μM AB23A, FFA + siGRP94‐2, and FFA + siGRP94‐2 + 80 μM AB23A. The results demonstrated that relative to the Control+siNC group, both the TG level and lipid deposition level in the FFA + siNC group exhibited a significant increase, which was subsequently reversed by AB23A intervention. However, silencing GRP94 annulled the ameliorative effect of AB23A on FFA‐induced L02 cell steatosis (Figure 3B–D). Western blot results unveiled that in comparison to the Control+siNC group, the protein expression of GRP94, p‐PERK/PERK, p‐eIF2α/eIF2α, ATF4, FBXO2, DERL, and HSP90α was upregulated in the FFA + siNC group, with AB23A intervention resulting in the downregulation of these proteins. Upon silencing GRP94, the expression of GRP94 protein in all groups nearly vanished, and the regulatory effects of AB23A on ERS‐related proteins (p‐PERK/PERK, p‐eIF2α/eIF2α, ATF4) and ERAD‐related proteins (FBXO2, DERL, HSP90α) were nullified (Figure 4A–C). This suggests that AB23A improves FFA‐induced L02 cell steatosis by downregulatingGRP94 and thereby enhancing ERS and ERAD.

### 3.4 AB23A Can Improve Steatosis in Hepatocytes of NASH Mice and Restore ER Homeostasis

**FIGURE 5:**
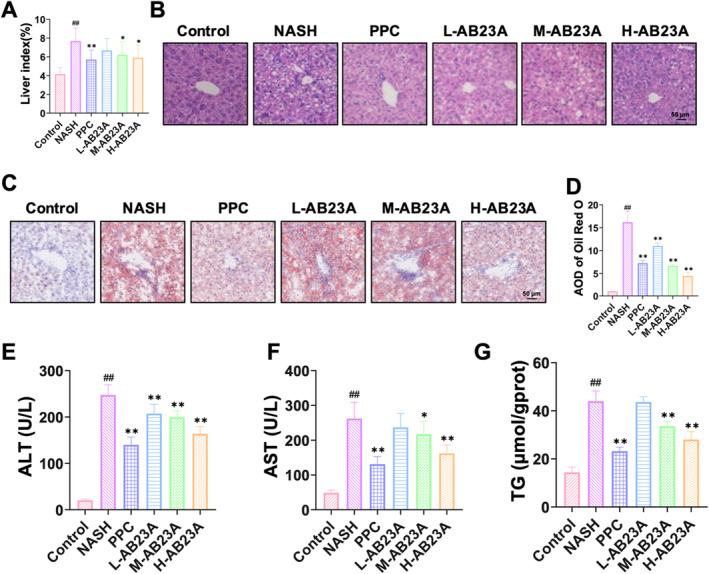
AB23A can improve hepatocyte steatosis in NASH mice. NASH mouse model was established through a MCD diet. Different doses of AB23A intervention were administered to evaluate the therapeutic effect of AB23A on NASH mice. (A) AB23A intervention promotes the recovery of liver index in NASH mice. (B–D) AB23A intervention reduces inflammatory infiltration in the liver of NASH mice (B) and improves lipid deposition in the liver (C, D). (E‐G) AB23A intervention improves serum ALT (E), AST activity (F), and liver TG levels (G). Data are presented as the mean ± SD. *n* = 10 per group. ^##^ *p* < 0.01 compared to the Control group; *p < 0.05, **p < 0.01 compared to the NASH group. One‐way or two‐way ANOVA followed by post hoc analysis with Tukey's test for comparison between more groups.

Building upon the in vitro experiments, we further substantiated the therapeutic efficacy of AB23A on NASH mice and its regulatory role in ER homeostasis through in vivo experiments. Firstly, we found that H‐AB23A can significantly alleviate MCD‐induced weight loss (Figure S1A). Furthermore, AB23A did not affect food intake (Figure S1B). Compared to the control group, the liver index of NASH mice exhibited a significant reduction. However, post AB23A treatment, the liver index of the mice exhibited some level of recovery, with the H‐AB23A group manifesting the most notable improvement (Figure 5A). HE staining and Oil Red O staining unveiled that while hepatic cords in the Control group were orderly arranged with a normal structure, the liver of NASH mice displayed severe fatty changes characterized by disorganized hepatic cord arrangement and substantial inflammatory cell infiltrations. Following AB23A intervention, these symptoms demonstrated varying degrees of improvement (Figure 5B–D). Furthermore, subsequent to AB23A treatment, the activities of ALT and AST in serum along with the liver TG level exhibited varying degrees of improvement relative to the NASH group, indicative of AB23A's ability to effectively restore liver function and ameliorate hepatic steatosis in NASH mice (Figure 5E–G).

**FIGURE 6:**
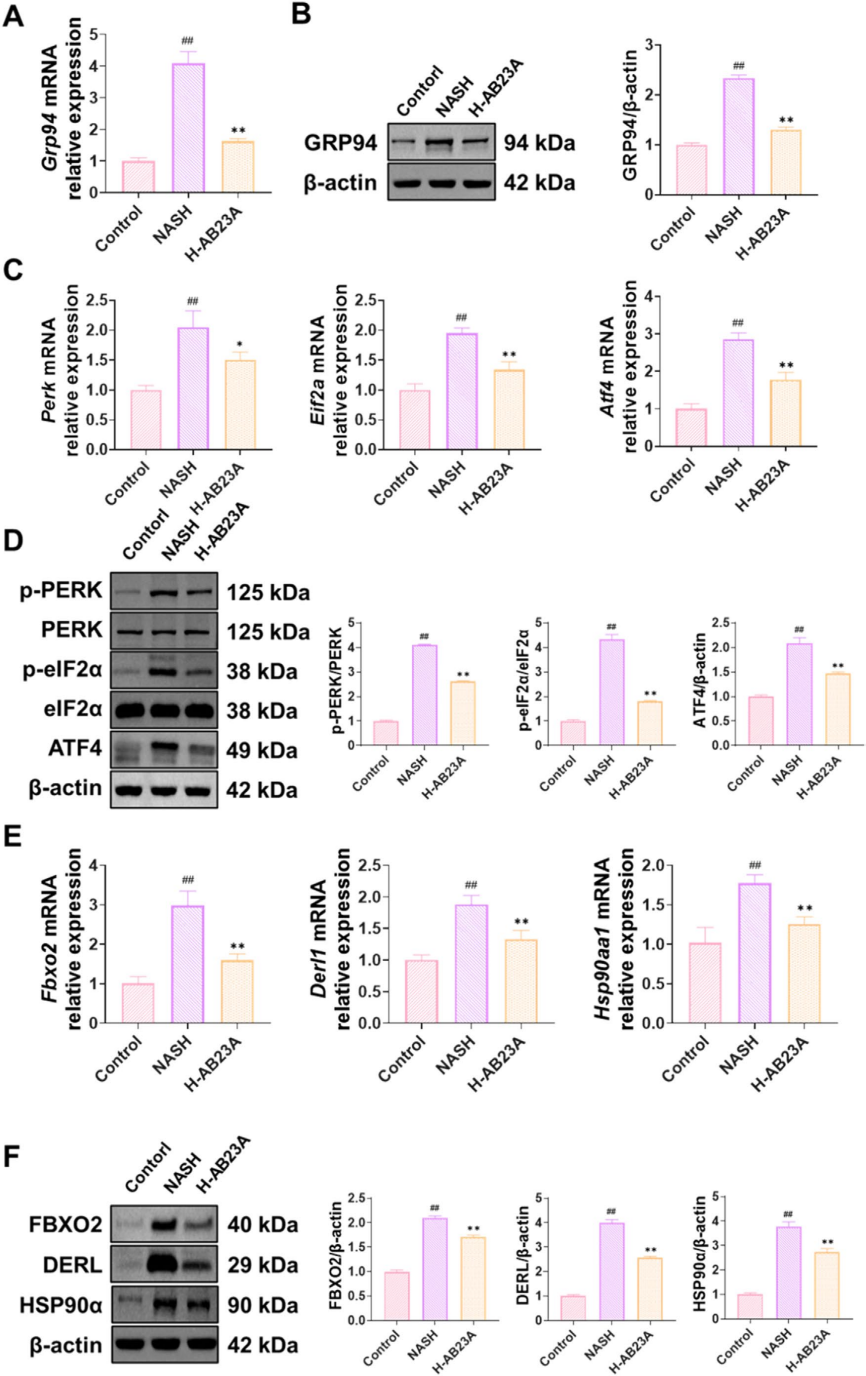
AB23A intervention can restore ER homeostasis in NASH mice hepatocytes. The effect of AB23A on ER homeostasis was investigated in hepatocytes of NASH mice using RT‐qPCR and Western blot. The results show that AB23A intervention downregulates the gene and protein expression of GRP94 (A, B), as well as the expression of ERS‐related genes and proteins (C, D) and ERAD‐related genes and proteins (E, F). Data are presented as the mean ± SD. *n* = 3 per group. ^##^ *p* < 0.01 compared to the Control group; *p < 0.05, **p < 0.01 compared to the NASH group. One‐way or two‐way ANOVA followed by post hoc analysis with Bonferroni test for comparison between more groups.

Additionally, we evaluated the effect of AB23A on ER homeostasis through RT‐qPCR and Western blot analyses. The outcomes revealed that compared to the Control group, the expression of GRP94, p‐PERK/PERK, p‐eIF2α/eIF2α, ATF4, FBXO2, DERL, and HSP90α genes and proteins was elevated in NASH mice, while AB23A intervention led to the downregulation of these genes and proteins in a dose‐dependent manner. Furthermore, we employed PPC as a positive control for intervention, and the results indicated no significant differences in the aforementioned indicators between the H‐AB23A group and the PPC group (Figure 6A–F).

## 4 Discussion

Numerous natural products, including betaine (Chen et al. 2021) and resveratrol (Binmowyna et al. 2024), have demonstrated efficacy in ameliorating hepatic steatosis in NASH animal models within laboratory settings. This study aims to assess the therapeutic potential of AB23A, the principal active compound derived from *Alisma orientale* (Sam.) Juzep., in NASH treatment through both in vitro and in vivo experimentation. In the in vitro phase, L02 cells subjected to FFA stimulation exhibited pronounced lipid accumulation, accompanied by an increase in cellular TG levels. In the subsequent in vivo phase, NASH‐afflicted mice, following a 6‐week MCD diet regimen, displayed marked hepatic steatosis, evidenced by a significant decrease in liver index and a notable rise in TG levels. These observations confirm the successful establishment of the NASH model in both experimental settings. Upon AB23A intervention, reductions in hepatic lipid deposition were evident in both in vitro and in vivo experiments, along with improvements in key indicators such as ALT, AST, and TG levels. These preliminary results underscore the potential therapeutic efficacy of AB23A in alleviating hepatic steatosis and addressing NASH.

Furthermore, transcriptome analysis following AB23A intervention revealed a significant downregulation in the expression of *GRP94*. Additionally, within the KEGG‐enriched pathways scrutinized, AB23A intervention notably modulated “Protein processing in endoplasmic reticulum,” primarily linked to improving ERS and ERAD function, crucial for maintaining ER homeostasis wherein GRP94 assumes significance (Ajoolabady et al. 2023; Krshnan et al. 2022). The ER serves as a pivotal organelle for lipid and sterol synthesis, processing, and metabolism, thereby playing a critical role in hepatic lipid homeostasis. In the progression of NASH, excessive lipid accumulation within hepatocytes precipitates ERS and ERAD dysregulation, exacerbating lipid metabolic disturbances and fostering NASH advancement (Dreher and Hoppe 2018; Lebeaupin et al. 2018; Rennert et al. 2020). Notably, interventions with ERS inducers have demonstrated a propensity to escalate hepatocytic lipid accumulation, perturbing ER equilibrium (Parafati et al. 2018). Conversely, inhibitory measures targeting GRP94 have exhibited efficacy in restoring ER homeostasis and ameliorating pathological sequelae across diverse ailments, including cancer and immune‐mediated inflammation (Pugh et al. 2022). The potential direct impact of AB23A on GRP94 awaits further validation through experiments such as cellular thermal shift assay (CETSA). Moreover, the function and activity of GRP94 may be influenced by more intricate mechanisms. For instance, 3‐(1,5‐diphenyl‐4,5‐dihydro‐1H‐pyrazol‐3‐yl)‐7‐hydroxy‐2H‐chromen‐2‐one (HCP1), a small molecule compound, can specifically bind to the third site of GRP94, thereby directly inhibiting its activity and affecting its role in protein folding and quality control within the ER (Wei et al. 2019). Additionally, Protein kinase CK2 (CK2), a multifunctional protein kinase composed of two α and two β subunits, has its CK2α subunit primarily responsible for the phosphorylation of GRP94 at Ser306 and Thr786 in the ER (Kim et al. 2024). Furthermore, GRP94 can be acetylated and binds to histone deacetylase 6 (HDAC6), a known activator of HSP90 proteins. Inhibition of HDAC6 leads to a decrease in PC2 levels, suggesting a synergistic role of HDAC6 and GRP94 in regulating PC2 levels (Yao, Outeda, et al. 2021; Yao, Ren, et al. 2021). Therefore, the upregulation of GRP94 may be associated with factors such as HCP1, CK2α, and HDAC6. These molecules could mediate AB23A's indirect effect on GRP94.

Moreover, our investigation revealed that AB23A intervention downregulated the expression of ERS‐associated proteins (PERK, eIF2α, ATF4). PERK, a type I transmembrane protein situated on the endoplasmic reticulum membrane, upon sensing unfolded protein accumulation, undergoes oligomerization and autophosphorylation, culminating in eIF2α phosphorylation and instigating the UPR to reinstate ER homeostasis (Fan and Jordan 2022). However, excessive ERS can precipitate a breakdown in the PERK‐eIF2α compensatory mechanism, with phosphorylated eIF2α activating ATF4, ultimately provoking apoptosis (Yao, Ren, et al. 2021). Studies corroborate that inhibitory measures targeting the PERK‐mediated signaling cascade can mitigate cell damage stemming from ERS (Rozpedek‐Kaminska et al. 2020). Notably, AB23A intervention downregulates PERK expression and its downstream effectors, suggesting a potential avenue through which AB23A may mitigate hepatic cell injury.

In addition, we observed that AB23A intervention downregulated the expression of ERAD‐related proteins (FBXO2, DERL, HSP90α). FBXO2, DERL, and HSP90α constitute integral components of ERAD. FBXO2, a ubiquitin ligase substrate adaptor protein predominantly localized in the cytoplasm, functions as a sensor for misfolded proteins, facilitating their identification and subsequent degradation (Yuan et al. 2017). Research indicates upregulated FBXO2 expression in liver tissue from NAFLD patients, with FBXO2 overexpression linked to lipid accumulation in HepG2 cells (Liu et al. 2023). DERL, a member of the Derlin family situated on the endoplasmic reticulum membrane, likewise participates in the degradation of misfolded proteins through ERAD. Studies underscore the pivotal role of Derlin family members in modulating NASH progression by regulating ERAD activation and ERS (Wang et al. 2024). HSP90α, a stress‐responsive subtype of the HSP90 family encoded by HSP90AA1, exhibits marked upregulation under cellular stress conditions, fostering cancer metastasis and inflammatory cascades (Zuehlke et al. 2015). AB23A's suppressive impact on FBXO2, DERL, and HSP90α underscores its potential in NASH treatment via anti‐ERAD mechanisms. Notably, GRP94, a central protein orchestrating both ERS and ERAD processes, emerges as a crucial regulator. Inhibition of GRP94 holds promise in ameliorating both pathways (Marzec et al. 2012). Notably, upon silencing GRP94, the ameliorative effects of AB23A intervention on ERS and ERAD diminished, implicating AB23A in restoring ER homeostasis by downregulatingGRP94, subsequently attenuating ERS and ERAD processes.

Based on our previous study (Li et al. 2024), AB23A's mechanism in treating NASH likely involves regulating alanine, aspartate, and glutamate metabolism, D‐glutamine and D‐glutamate pathways, and arginine biosynthesis. Interestingly, glutamine has been proved to attenuates endoplasmic reticulum stress and apoptosis in TNBS‐induced colitis (Crespo et al. 2012). Therefore, it remains to be investigated whether AB23A's ability to inhibit GRP94 could lead to the upregulation of glutamine, which may further suppress ER stress and mitigate NASH progression. Moreover, Pharmacokinetic studies are crucial for optimizing dosing regimens, understanding the drug's bioavailability, and ensuring its therapeutic potential is achieved without adverse effects. We plan to assess the pharmacokinetic properties of AB23A through in vivo experiments in future studies, which will provide valuable insights for its application.

**FIGURE 7:**
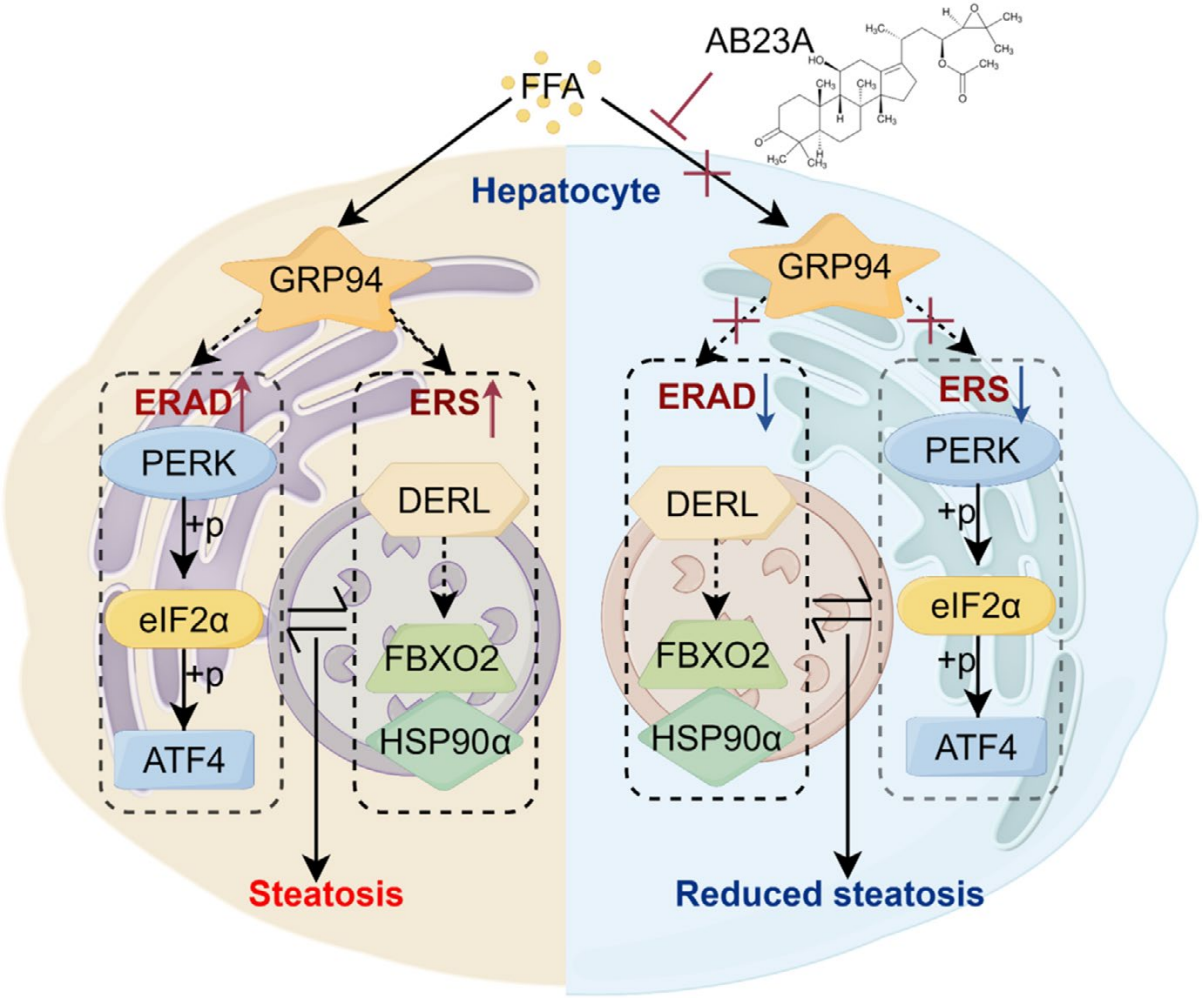
AB23A down‐regulated GRP94, thereby suppressing ERS and ERAD, restoring ER homeostasis, and improving hepatocyte steatosis.

In conclusion, our study, conducted through in vitro and in vivo experiments, underscores the potential of AB23A in ameliorating hepatic steatosis, likely via GRP94 inhibition, consequently impeding ERS and ERAD processes while restoring ER equilibrium (Figure 7). Nevertheless, there remains scope for refinement in our experimental approaches.

## Author Contributions

**Fei Qu:** conceptualization (equal), data curation (equal), writing – original draft (equal). **Yuming Wang:** data curation (equal), formal analysis (equal), writing – original draft (equal). **Yanping Zhang:** investigation (equal), project administration (equal). **Feng Chen:** methodology (equal), software (equal). **Yuanliang Ai:** investigation (equal), methodology (equal). **Weibo Wen:** conceptualization (equal), supervision (equal), validation (equal). **Jiabao Liao:** resources (equal), software (equal). **Hanzhou Li:** investigation (equal), methodology (equal). **Huan Pei:** validation (equal), visualization (equal). **Mingxi Lu:** methodology (equal). **Ling Yang:** supervision (equal), validation (equal). **Ning Wang:** resources (equal), software (equal). **Huantian Cui:** resources (equal), visualization (equal), writing – review and editing (equal).

## Conflicts of Interest

The authors declare no conflicts of interest.

## Supporting information


Appendix S1.

## Data Availability

Data will be made available upon request.

## References

[fsn370086-bib-0001] Ajoolabady, A. , N. Kaplowitz , C. Lebeaupin , et al. 2023. “Endoplasmic Reticulum Stress in Liver Diseases.” Hepatology 77, no. 2: 619–639. 10.1002/hep.32562. 35524448 PMC9637239

[fsn370086-bib-0002] Binmowyna, M. N. , N. A. Alfaris , E. A. Al‐Sanea , J. Z. Altamimi , and T. S. Aldayel . 2024. “Resveratrol Attenuates Against High‐Fat‐Diet‐Promoted Non‐Alcoholic Fatty Liver Disease in Rats Mainly by Targeting the miR‐34a/SIRT1 Axis.” Archives of Physiology and Biochemistry 130, no. 3: 300–315. 10.1080/13813455.2022.2046106. 35254877

[fsn370086-bib-0003] Chen, W. , X. Zhang , M. Xu , et al. 2021. “Betaine Prevented High‐Fat Diet‐Induced NAFLD by Regulating the FGF10/AMPK Signaling Pathway in ApoE(−/−) Mice.” European Journal of Nutrition 60, no. 3: 1655–1668. 10.1007/s00394-020-02362-6. 32808060

[fsn370086-bib-0004] Crespo, I. , B. San‐Miguel , C. Prause , et al. 2012. “Glutamine Treatment Attenuates Endoplasmic Reticulum Stress and Apoptosis in TNBS‐Induced Colitis.” PLoS One 7, no. 11: e50407. 10.1371/journal.pone.0050407. 23209735 PMC3508929

[fsn370086-bib-0005] Dreher, L. S. , and T. Hoppe . 2018. “Hepatic ERAD Takes Control of the Organism.” EMBO Journal 37, no. 22: e100676. 10.15252/embj.2018100676. 30389670 PMC6236333

[fsn370086-bib-0006] Fan, P. , and V. C. Jordan . 2022. “PERK, Beyond an Unfolded Protein Response Sensor in Estrogen‐Induced Apoptosis in Endocrine‐Resistant Breast Cancer.” Molecular Cancer Research 20, no. 2: 193–201. 10.1158/1541-7786.MCR-21-0702. 34728551 PMC8816868

[fsn370086-bib-0007] Kim, H. , Y. Kim , and S. Hong . 2024. “CK2alpha‐Mediated Phosphorylation of GRP94 Facilitates the Metastatic Cascade in Triple‐Negative Breast Cancer.” Cell Death Discovery 10, no. 1: 185. 10.1038/s41420-024-01956-x. 38649679 PMC11035675

[fsn370086-bib-0008] Krshnan, L. , M. L. van de Weijer , and P. Carvalho . 2022. “Endoplasmic Reticulum‐Associated Protein Degradation.” Cold Spring Harbor Perspectives in Biology 14, no. 12: a041247. 10.1101/cshperspect.a041247. 35940909 PMC9732900

[fsn370086-bib-0009] Lebeaupin, C. , D. Vallee , D. Rousseau , et al. 2018. “Bax Inhibitor‐1 Protects From Nonalcoholic Steatohepatitis by Limiting Inositol‐Requiring Enzyme 1 Alpha Signaling in Mice.” Hepatology 68, no. 2: 515–532. 10.1002/hep.29847. 29457838

[fsn370086-bib-0010] Li, H. , Y. Wang , Y. Wang , et al. 2024. “Mechanical Study of Alisol B 23‐Acetate on Methionine and Choline Deficient Diet‐Induced Nonalcoholic Steatohepatitis Based on Untargeted Metabolomics.” Biomedical Chromatography 38, no. 1: e5763. 10.1002/bmc.5763. 37858975

[fsn370086-bib-0011] Li, J. , X. Li , D. Liu , et al. 2020. “Phosphorylation of eIF2alpha Signaling Pathway Attenuates Obesity‐Induced Non‐alcoholic Fatty Liver Disease in an ER Stress and Autophagy‐Dependent Manner.” Cell Death & Disease 11, no. 12: 1069. 10.1038/s41419-020-03264-5. 33318479 PMC7736876

[fsn370086-bib-0012] Liu, Z. , N. Y. Chen , Z. Zhang , S. Zhou , and S. Y. Hu . 2023. “F‐Box Only Protein 2 Exacerbates Non‐alcoholic Fatty Liver Disease by Targeting the Hydroxyl CoA Dehydrogenase Alpha Subunit.” World Journal of Gastroenterology 29, no. 28: 4433–4450. 10.3748/wjg.v29.i28.4433. 37576703 PMC10415968

[fsn370086-bib-0013] Marzec, M. , D. Eletto , and Y. Argon . 2012. “GRP94: An HSP90‐Like Protein Specialized for Protein Folding and Quality Control in the Endoplasmic Reticulum.” Biochimica et Biophysica Acta 1823, no. 3: 774–787. 10.1016/j.bbamcr.2011.10.013. 22079671 PMC3443595

[fsn370086-bib-0014] Okada, L. , C. P. Oliveira , J. T. Stefano , et al. 2018. “Omega‐3 PUFA Modulate Lipogenesis, ER Stress, and Mitochondrial Dysfunction Markers in NASH—Proteomic and Lipidomic Insight.” Clinical Nutrition 37, no. 5: 1474–1484. 10.1016/j.clnu.2017.08.031. 29249532

[fsn370086-bib-0015] Parafati, M. , R. J. Kirby , S. Khorasanizadeh , F. Rastinejad , and S. Malany . 2018. “A Nonalcoholic Fatty Liver Disease Model in Human Induced Pluripotent Stem Cell‐Derived Hepatocytes, Created by Endoplasmic Reticulum Stress‐Induced Steatosis.” Disease Models & Mechanisms 11, no. 9: dmm033530. 10.1242/dmm.033530. 30254132 PMC6176998

[fsn370086-bib-0016] Povsic, M. , O. Y. Wong , R. Perry , and J. Bottomley . 2019. “A Structured Literature Review of the Epidemiology and Disease Burden of Non‐Alcoholic Steatohepatitis (NASH).” Advances in Therapy 36, no. 7: 1574–1594. 10.1007/s12325-019-00960-3. 31065991 PMC6824389

[fsn370086-bib-0017] Pugh, K. W. , M. Alnaed , C. M. Brackett , and B. Blagg . 2022. “The Biology and Inhibition of Glucose‐Regulated Protein 94/gp96.” Medicinal Research Reviews 42, no. 6: 2007–2024. 10.1002/med.21915. 35861260 PMC10003671

[fsn370086-bib-0018] Rennert, C. , T. Heil , G. Schicht , et al. 2020. “Prolonged Lipid Accumulation in Cultured Primary Human Hepatocytes Rather Leads to ER Stress Than Oxidative Stress.” International Journal of Molecular Sciences 21, no. 19: 7097. 10.3390/ijms21197097. 32993055 PMC7582586

[fsn370086-bib-0019] Rozpedek‐Kaminska, W. , N. Siwecka , A. Wawrzynkiewicz , et al. 2020. “The PERK‐Dependent Molecular Mechanisms as a Novel Therapeutic Target for Neurodegenerative Diseases.” International Journal of Molecular Sciences 21, no. 6: 2108. 10.3390/ijms21062108. 32204380 PMC7139310

[fsn370086-bib-0020] Shaukat, A. , I. Shaukat , S. A. Rajput , et al. 2021. “Ginsenoside Rb1 Protects From *Staphylococcus aureus*‐Induced Oxidative Damage and Apoptosis Through Endoplasmic Reticulum‐Stress and Death Receptor‐Mediated Pathways.” Ecotoxicology and Environmental Safety 219: 112353. 10.1016/j.ecoenv.2021.112353. 34034046

[fsn370086-bib-0021] Sheka, A. C. , O. Adeyi , J. Thompson , B. Hameed , P. A. Crawford , and S. Ikramuddin . 2020. “Nonalcoholic Steatohepatitis: A Review.” JAMA: The Journal of the American Medical Association 323, no. 12: 1175–1183. 10.1001/jama.2020.2298. 32207804

[fsn370086-bib-0022] Wang, T. , D. Wang , G. Kuang , et al. 2024. “Derlin‐1 Promotes Diet‐Induced Non‐Alcoholic Fatty Liver Disease via Increasing RIPK3‐Mediated Necroptosis.” Free Radical Biology and Medicine 217: 29–47. 10.1016/j.freeradbiomed.2024.03.014. 38522486

[fsn370086-bib-0023] Wei, Q. , J. Zhang , L. Su , et al. 2019. “Low‐Concentration HCP1 Inhibits Apoptosis in Vascular Endothelial Cells.” Biochemical and Biophysical Research Communications 511, no. 1: 92–98. 10.1016/j.bbrc.2019.02.003. 30770100

[fsn370086-bib-0024] Wei, S. , L. Wang , P. C. Evans , and S. Xu . 2024. “NAFLD and NASH: Etiology, Targets and Emerging Therapies.” Drug Discovery Today 29, no. 3: 103910. 10.1016/j.drudis.2024.103910. 38301798

[fsn370086-bib-0026] Xu, Y. , X. Li , F. Cheng , et al. 2024. “Heat Shock Protein gp96 Drives Natural Killer Cell Maturation and Anti‐Tumor Immunity by Counteracting Trim28 to Stabilize Eomes.” Nature Communications 15, no. 1: 1106. 10.1038/s41467-024-45426-5. PMC10847424 38321029

[fsn370086-bib-0027] Yao, Q. , P. Outeda , H. Xu , et al. 2021. “Polycystin‐1 Dependent Regulation of Polycystin‐2 via GRP94, a Member of HSP90 Family That Resides in the Endoplasmic Reticulum.” FASEB Journal: Official Publication of the Federation of American Societies for Experimental Biology 35, no. 10: e21865. 10.1096/fj.202100325RR. 34486178 PMC8477617

[fsn370086-bib-0028] Yao, R. Q. , C. Ren , Z. F. Xia , and Y. M. Yao . 2021. “Organelle‐Specific Autophagy in Inflammatory Diseases: A Potential Therapeutic Target Underlying the Quality Control of Multiple Organelles.” Autophagy 17, no. 2: 385–401. 10.1080/15548627.2020.1725377. 32048886 PMC8007140

[fsn370086-bib-0029] Yin, S. , L. Li , Y. Tao , et al. 2021. “The Inhibitory Effect of Artesunate on Excessive Endoplasmic Reticulum Stress Alleviates Experimental Colitis in Mice.” Frontiers in Pharmacology 12: 629798. 10.3389/fphar.2021.629798. 33767628 PMC7985062

[fsn370086-bib-0030] Yuan, L. , Z. Song , X. Deng , et al. 2017. “Genetic Analysis of FBXO2, FBXO6, FBXO12, and FBXO41 Variants in Han Chinese Patients With Sporadic Parkinson's Disease.” Neuroscience Bulletin 33, no. 5: 510–514. 10.1007/s12264-017-0122-5. 28341977 PMC5636729

[fsn370086-bib-0031] Zhou, X. , M. Ren , J. Yang , H. Pan , M. Yu , and F. Ji . 2021. “Curcumin Improves Epithelial Barrier Integrity of Caco‐2 Monolayers by Inhibiting Endoplasmic Reticulum Stress and Subsequent Apoptosis.” Gastroenterology Research and Practice 2021: 5570796. 10.1155/2021/5570796. 34659400 PMC8514927

[fsn370086-bib-0032] Zuehlke, A. D. , K. Beebe , L. Neckers , and T. Prince . 2015. “Regulation and Function of the Human HSP90AA1 Gene.” Gene 570, no. 1: 8–16. 10.1016/j.gene.2015.06.018. 26071189 PMC4519370

