# Supplementary material for: Alisol B 23‐Acetate Down‐Regulated GRP94 to Restore Endoplasmic Reticulum Homeostasis on Non‐Alcoholic Steatohepatitis
Source: Food Sci Nutr. 2025 Mar 5;13(3):e70086. doi: 10.1002/fsn3.70086 (PMC11883119; doi:10.1002/fsn3.70086)
Supplement: Supplementary file 1 — Appendix S1. [file FSN3-13-e70086-s001.docx]

**Supplementary materials**

**1 Reagents**

MCD chow was purchased from Sibeifu Bioscience Co., Ltd. (Beijing, China). Polyene phosphatidylcholine (PPC) was purchased from Sanofi. (Beijing, China). AB23A (C_32_H_50_O_5_, molecular weight 514.75 Da, purity ≥ 98%) was purchased from Push Bio-Technology (Chengdu, China). L02 cells was provided by iCell Bioscience,Inc. (Shanghai, China). RPMI-1640 medium (R20161), fetal bovine serum (R28177), penicillin and streptomycin (S17032) were purchased from Shanghai yuanye Bio-Technology Co., Ltd. SiGRP94 plasmid and the empty vector (siNC) were provided by Azenta (Tianjin, China). MTT (HY-15924) were provided by Medchemexpress (Shanghai, China). Lipofectamine 2000 Reagent ([11668030](https://www.thermofisher.cn/order/catalog/product/11668030)) was purchased from Thermo Fisher Scientific (Shanghai, China). Triglyceride (TG, A110-1-1), alanine aminotransferase (ALT, C009-1-1), aspartate aminotransferase (AST, C0101-2-1) were purchased from Nanjing Jiancheng Biological Engineering Institute (Nanjing, China). Primary antibodies: rabbit anti-GRP94 (ab238126), rabbit anti-PERK (ab229912), rabbit anti-eIF2α (ab169528), rabbit anti-DERL1 (ab176732) were purchased from Abcam (Shanghai, China); rabbit anti-p-PERK (82534-1-RR), rabbit anti-p-eIF2α (28740-1-AP), rabbit anti-ATF4 (10835-1-AP), rabbit anti-FBXO2 (14590-1-AP) were purchased from Proteintech (Wuhan, China); rabbit anti-HSP90α (K106929P) and rabbit anti-β-actin (K101527P) were purchased from Solarbio (Beijing, China). Secondary antibody for goat anti-mouse IgG H&L (ab6785) and goat anti-rabbit IgG H&L (ab207995) were purchased from Abcam (Shanghai, China). Total RNA extraction, first-strand cDNA reverse transcription, polymerase chain reaction (PCR) kits and primers were obtained from TianGen Biotechnology Co., Ltd. (Beijing, China).

**2 Primer sequence**

**Table S1 Primer sequence**

| Genes | Primer sequence (5’-3’) | |
| --- | --- | --- |
| *Actb* | Forward: | CCCCTGAACCCTAAGGCCA |
|  | Reverse: | ATGGCTACGTACATGGCTGG |
| *Grp94* | Forward: | GTTCGTCAGAGCTGATGATGAA |
|  | Reverse: | GCGTTTAACCCATCCAACTGAAT |
| *Perk* | Forward: | CGCGTCGGAGACAGTGTTT |
|  | Reverse: | GTCCTCCACGGTCACTTCG |
| *Eif2a* | Forward: | CACGGTGCTTCCCAGAGAATC |
|  | Reverse: | GTCCCTTGTTAGCGACATTGA |
| *Atf4* | Forward: | CCTGAACAGCGAAGTGTTGG |
|  | Reverse: | TGGAGAACCCATGAGGTTTCAA |
| *Fbxo2* | Forward: | ATGGGGTGGAATTTACCCAAGA |
|  | Reverse: | GACCCGAGTACCAGTCCTTCA |
| *Derl* | Forward: | AGGAGGGTTCTTTCCGTGG |
|  | Reverse: | ACGGCTCCATACATAGACCAG |
| *Hsp90aa1* | Forward: | GACGCTCTGGATAAAATCCGTT |
|  | Reverse: | TGGGAATGAGATTGATGTGCAG |

**3 Differential genes**

**Table S2 Differential genes**

| Gene Name | FFA vs Control | | FFA+80 μM AB23A vs FFA | |
| --- | --- | --- | --- | --- |
|  | Log_2_FoldChange | padj | Log_2_FoldChange | padj |
| ANKIB1 | -1.016 | 0.000 | 1.072 | 0.000 |
| KRIT1 | -2.140 | 0.000 | 2.026 | 0.000 |
| MTMR7 | -2.078 | 0.002 | 1.983 | 0.001 |
| ARF5 | 1.069 | 0.000 | -1.066 | 0.000 |
| RECQL | -2.002 | 0.000 | 1.777 | 0.000 |
| VPS50 | -1.187 | 0.000 | 1.051 | 0.000 |
| RPAP3 | -2.789 | 0.000 | 2.752 | 0.000 |
| KMT2E | -2.897 | 0.000 | 2.697 | 0.000 |
| IBTK | -1.348 | 0.000 | 1.532 | 0.000 |
| ZNF195 | -2.271 | 0.000 | 2.479 | 0.000 |
| MYCBP2 | -2.448 | 0.000 | 2.459 | 0.000 |
| ZFX | -2.838 | 0.000 | 1.844 | 0.000 |
| DBF4 | -1.980 | 0.000 | 1.664 | 0.000 |
| MYLIP | -2.942 | 0.000 | 1.745 | 0.000 |
| SPAG9 | -1.535 | 0.000 | 1.612 | 0.000 |
| IL32 | 5.470 | 0.000 | -2.614 | 0.000 |
| MAPK8IP2 | 1.249 | 0.000 | -1.292 | 0.000 |
| SEC62 | -3.003 | 0.000 | 2.719 | 0.000 |
| REV3L | -3.545 | 0.000 | 3.300 | 0.000 |
| MASP2 | -3.460 | 0.000 | 2.978 | 0.000 |
| BAZ1B | -1.736 | 0.000 | 1.896 | 0.000 |
| SLC6A13 | -2.379 | 0.000 | 2.519 | 0.000 |
| PIK3C2A | -2.632 | 0.000 | 2.522 | 0.000 |
| ANLN | -3.379 | 0.000 | 2.460 | 0.000 |
| BRCA1 | -1.908 | 0.000 | 2.282 | 0.000 |
| MAP4K5 | -1.901 | 0.000 | 1.520 | 0.000 |
| CLK1 | -1.612 | 0.000 | 1.218 | 0.000 |
| UFL1 | -1.702 | 0.000 | 1.575 | 0.000 |
| MATR3 | -3.327 | 0.000 | 3.125 | 0.000 |
| MRE11 | -1.624 | 0.000 | 1.528 | 0.000 |
| RB1CC1 | -4.657 | 0.000 | 4.377 | 0.000 |
| BIRC3 | -1.638 | 0.000 | 1.638 | 0.000 |
| AKAP11 | -1.818 | 0.000 | 2.016 | 0.000 |
| DEPDC1 | -1.678 | 0.000 | 1.413 | 0.000 |
| TYMP | 1.171 | 0.000 | -1.716 | 0.000 |
| SEC63 | -1.751 | 0.000 | 2.277 | 0.000 |
| RTEL1-TNFRSF6B | -2.607 | 0.006 | 2.066 | 0.022 |
| BCLAF1 | -3.688 | 0.000 | 3.108 | 0.000 |
| RABEP1 | -1.313 | 0.000 | 1.334 | 0.000 |
| IKZF2 | -2.948 | 0.000 | 2.349 | 0.000 |
| CENPQ | -2.275 | 0.000 | 1.919 | 0.000 |
| ARID4A | -5.088 | 0.000 | 4.813 | 0.000 |
| LRRC7 | -3.339 | 0.008 | 3.460 | 0.001 |
| UBA6 | -1.542 | 0.000 | 1.166 | 0.000 |
| SLC4A7 | -1.074 | 0.000 | 1.134 | 0.000 |
| RFC1 | -3.091 | 0.000 | 3.046 | 0.000 |
| CUL3 | -1.507 | 0.000 | 1.122 | 0.000 |
| BOD1L1 | -3.375 | 0.000 | 3.160 | 0.000 |
| MTREX | -1.428 | 0.000 | 1.108 | 0.000 |
| ZFYVE16 | -2.039 | 0.000 | 1.886 | 0.000 |
| RAI14 | -2.184 | 0.000 | 2.646 | 0.000 |
| SPDL1 | -2.779 | 0.000 | 2.276 | 0.000 |
| RTN4R | 2.182 | 0.000 | -1.255 | 0.000 |
| CAPG | 1.308 | 0.000 | -1.181 | 0.000 |
| AP2S1 | 1.145 | 0.000 | -1.190 | 0.000 |
| CNTLN | -1.707 | 0.000 | 1.751 | 0.000 |
| DSG2 | -1.130 | 0.000 | 1.256 | 0.000 |
| OFD1 | -3.312 | 0.000 | 3.636 | 0.000 |
| YTHDC2 | -2.183 | 0.000 | 2.037 | 0.000 |
| FAM214A | -2.146 | 0.000 | 2.026 | 0.000 |
| ARAP2 | -1.659 | 0.000 | 1.910 | 0.000 |
| TPR | -4.507 | 0.000 | 4.237 | 0.000 |
| ZNF800 | -1.370 | 0.000 | 1.546 | 0.000 |
| RSF1 | -3.088 | 0.000 | 3.062 | 0.000 |
| VPS13D | -1.452 | 0.000 | 1.240 | 0.000 |
| R3HDM1 | -1.047 | 0.000 | 1.352 | 0.000 |
| ADAMTS6 | -2.356 | 0.000 | 1.850 | 0.000 |
| TNFRSF9 | 7.077 | 0.000 | -1.083 | 0.000 |
| ARID1B | -1.342 | 0.000 | 1.386 | 0.000 |
| HERPUD1 | 2.108 | 0.000 | -1.553 | 0.000 |
| POLQ | -2.747 | 0.000 | 2.590 | 0.000 |
| CYBA | 1.098 | 0.000 | -1.142 | 0.000 |
| MPHOSPH9 | -2.893 | 0.000 | 2.667 | 0.000 |
| PLEKHA5 | -2.647 | 0.000 | 2.819 | 0.000 |
| MSMO1 | 1.726 | 0.000 | -2.454 | 0.000 |
| NNAT | -2.591 | 0.004 | 2.471 | 0.005 |
| NRIP2 | -2.220 | 0.000 | 1.766 | 0.001 |
| LAMA3 | -1.192 | 0.000 | 1.421 | 0.000 |
| KCNQ1 | 1.376 | 0.001 | -1.365 | 0.000 |
| THRAP3 | -1.735 | 0.000 | 1.227 | 0.000 |
| PHPT1 | 1.063 | 0.000 | -1.181 | 0.000 |
| ARID4B | -3.667 | 0.000 | 3.567 | 0.000 |
| SYNE2 | -3.494 | 0.000 | 3.020 | 0.000 |
| NOP58 | -3.367 | 0.000 | 3.320 | 0.000 |
| TAB2 | -1.001 | 0.000 | 1.116 | 0.000 |
| EIF2AK2 | 1.729 | 0.000 | -1.417 | 0.000 |
| KMT2C | -1.879 | 0.000 | 1.735 | 0.000 |
| ZNF280C | -1.335 | 0.000 | 1.432 | 0.000 |
| TRAF1 | 1.916 | 0.002 | -1.108 | 0.037 |
| PPP1R12A | -4.160 | 0.000 | 3.506 | 0.000 |
| WNK1 | -1.020 | 0.000 | 1.080 | 0.000 |
| CCAR1 | -4.395 | 0.000 | 4.396 | 0.000 |
| QSER1 | -2.370 | 0.000 | 2.223 | 0.000 |
| CDH3 | 1.109 | 0.040 | -1.329 | 0.008 |
| GPBP1 | -1.026 | 0.000 | 1.115 | 0.000 |
| ZNF112 | -2.148 | 0.026 | 2.039 | 0.017 |
| MRPS24 | 1.829 | 0.028 | -1.683 | 0.016 |
| LIMCH1 | -2.131 | 0.000 | 1.859 | 0.000 |
| SLC12A2 | -1.296 | 0.000 | 1.497 | 0.000 |
| FAR2 | -2.235 | 0.000 | 1.832 | 0.005 |
| PMS1 | -1.705 | 0.000 | 1.850 | 0.000 |
| PKN2 | -2.283 | 0.000 | 1.790 | 0.000 |
| MCM10 | -1.442 | 0.000 | 1.590 | 0.000 |
| SPEN | -1.621 | 0.000 | 1.410 | 0.000 |
| ZC3H15 | -2.134 | 0.000 | 1.914 | 0.000 |
| SLK | -3.952 | 0.000 | 3.803 | 0.000 |
| ASPM | -5.969 | 0.000 | 4.643 | 0.000 |
| ZBTB11 | -1.962 | 0.000 | 1.645 | 0.000 |
| ATXN3 | -1.166 | 0.000 | 1.098 | 0.000 |
| GOLGA5 | -1.135 | 0.000 | 1.105 | 0.000 |
| THUMPD1 | -1.690 | 0.000 | 1.376 | 0.000 |
| ATG2B | -1.156 | 0.000 | 1.153 | 0.000 |
| ARFGEF1 | -2.205 | 0.000 | 2.297 | 0.000 |
| MYO9A | -2.597 | 0.000 | 2.515 | 0.000 |
| CACNB1 | 1.806 | 0.000 | -1.027 | 0.000 |
| EVI5 | -2.101 | 0.000 | 2.208 | 0.000 |
| DHX29 | -2.389 | 0.000 | 2.201 | 0.000 |
| DNTTIP2 | -4.571 | 0.000 | 4.251 | 0.000 |
| TP53BP1 | -1.394 | 0.000 | 1.145 | 0.000 |
| RRP15 | -1.632 | 0.000 | 1.685 | 0.000 |
| NAV3 | -1.551 | 0.000 | 1.464 | 0.000 |
| ROCK1 | -4.154 | 0.000 | 4.210 | 0.000 |
| IFI35 | 1.525 | 0.000 | -1.049 | 0.000 |
| KIF2A | -1.474 | 0.000 | 1.364 | 0.000 |
| IFT80 | -2.108 | 0.000 | 2.205 | 0.000 |
| MAST4 | -1.519 | 0.000 | 1.048 | 0.006 |
| TGFBR3 | -1.568 | 0.000 | 1.040 | 0.000 |
| LRP6 | -1.180 | 0.000 | 1.470 | 0.000 |
| NUCB2 | -2.372 | 0.000 | 2.764 | 0.000 |
| EXOC5 | -1.251 | 0.000 | 1.021 | 0.000 |
| WIPI1 | 1.476 | 0.000 | -1.026 | 0.000 |
| RAD18 | -1.508 | 0.000 | 1.224 | 0.000 |
| ATP2B1 | -1.679 | 0.000 | 1.617 | 0.000 |
| SNX13 | -1.605 | 0.000 | 1.568 | 0.000 |
| HLTF | -2.075 | 0.000 | 2.217 | 0.000 |
| AFF4 | -1.435 | 0.000 | 1.601 | 0.000 |
| SMC1A | -1.530 | 0.000 | 1.406 | 0.000 |
| HMMR | -4.690 | 0.000 | 4.113 | 0.000 |
| MOV10L1 | 2.629 | 0.005 | -1.629 | 0.028 |
| PANX2 | 1.197 | 0.000 | -1.140 | 0.000 |
| SMARCE1 | -1.004 | 0.049 | 1.250 | 0.001 |
| GSDMB | -2.055 | 0.011 | 2.162 | 0.003 |
| KDM5A | -1.408 | 0.000 | 1.362 | 0.000 |
| PPP2R3A | -1.559 | 0.000 | 1.487 | 0.000 |
| ZNF532 | -1.209 | 0.000 | 1.108 | 0.000 |
| MYDGF | 1.018 | 0.000 | -1.014 | 0.000 |
| EIF4G3 | -2.256 | 0.000 | 2.189 | 0.000 |
| SEMA3A | -1.875 | 0.000 | 1.891 | 0.000 |
| SEMA3C | -1.304 | 0.000 | 1.109 | 0.000 |
| ZNF638 | -3.852 | 0.000 | 3.836 | 0.000 |
| RASAL2 | -1.481 | 0.000 | 1.559 | 0.000 |
| ZNF37A | -2.969 | 0.000 | 3.416 | 0.000 |
| FRYL | -2.043 | 0.000 | 2.004 | 0.000 |
| DLG1 | -1.327 | 0.000 | 1.012 | 0.000 |
| BCAP29 | -1.088 | 0.000 | 1.319 | 0.000 |
| SEC31B | -2.166 | 0.001 | 2.609 | 0.000 |
| SPAG5 | -2.196 | 0.000 | 1.219 | 0.000 |
| GPATCH1 | -1.452 | 0.000 | 1.087 | 0.000 |
| MBNL3 | -2.051 | 0.000 | 1.151 | 0.000 |
| RAP1GAP | 1.175 | 0.000 | -1.280 | 0.000 |
| TOP2B | -3.078 | 0.000 | 2.939 | 0.000 |
| PPP1R12B | -1.445 | 0.000 | 1.248 | 0.000 |
| USP33 | -1.117 | 0.000 | 1.001 | 0.000 |
| FAM76B | -1.463 | 0.000 | 1.142 | 0.000 |
| N4BP2 | -3.208 | 0.000 | 3.227 | 0.000 |
| PCM1 | -3.905 | 0.000 | 4.199 | 0.000 |
| TNRC6C | -1.346 | 0.000 | 1.507 | 0.000 |
| TOLLIP | 1.024 | 0.000 | -1.030 | 0.000 |
| THOC1 | -1.258 | 0.000 | 1.153 | 0.000 |
| FDFT1 | 1.144 | 0.000 | -1.977 | 0.000 |
| CARMIL1 | -1.485 | 0.000 | 1.142 | 0.000 |
| EPB41L2 | -1.983 | 0.000 | 1.510 | 0.000 |
| PTPRH | 3.466 | 0.000 | -1.127 | 0.043 |
| CRYBG3 | -3.231 | 0.000 | 3.404 | 0.000 |
| RIF1 | -3.551 | 0.000 | 3.389 | 0.000 |
| SMARCA2 | -1.062 | 0.000 | 1.470 | 0.000 |
| PUM3 | -2.846 | 0.000 | 2.968 | 0.000 |
| HSP90AA1 | 1.440 | 0.000 | -1.572 | 0.000 |
| NDC80 | -2.921 | 0.000 | 2.366 | 0.000 |
| MAGI3 | -1.574 | 0.000 | 1.598 | 0.000 |
| CXCL2 | 1.094 | 0.000 | 1.327 | 0.000 |
| COL4A4 | -2.071 | 0.000 | 1.138 | 0.000 |
| ZNF510 | -1.550 | 0.000 | 1.357 | 0.000 |
| CCNT2 | -1.628 | 0.000 | 1.440 | 0.000 |
| COBLL1 | -2.052 | 0.000 | 2.579 | 0.000 |
| ERC1 | -1.145 | 0.000 | 1.211 | 0.000 |
| DOP1A | -1.198 | 0.000 | 1.414 | 0.000 |
| KAT6A | -1.287 | 0.000 | 1.313 | 0.000 |
| TUT7 | -2.733 | 0.000 | 2.573 | 0.000 |
| DIS3 | -2.162 | 0.000 | 1.919 | 0.000 |
| PIBF1 | -4.615 | 0.000 | 4.824 | 0.000 |
| TDRD3 | -1.475 | 0.000 | 1.496 | 0.000 |
| PDS5B | -3.568 | 0.000 | 3.313 | 0.000 |
| RPS5 | 1.154 | 0.000 | -1.380 | 0.000 |
| FAT1 | -1.596 | 0.000 | 1.630 | 0.000 |
| YTHDC1 | -2.377 | 0.000 | 2.021 | 0.000 |
| REST | -3.136 | 0.000 | 2.740 | 0.000 |
| GSTP1 | 1.011 | 0.000 | -1.234 | 0.000 |
| NCOA1 | -1.102 | 0.000 | 1.273 | 0.000 |
| ATRX | -4.304 | 0.000 | 4.022 | 0.000 |
| MECOM | -2.136 | 0.000 | 1.599 | 0.000 |
| TXLNG | -1.738 | 0.000 | 2.048 | 0.000 |
| HSD17B14 | 2.583 | 0.027 | -2.773 | 0.007 |
| FTL | 1.056 | 0.000 | -1.210 | 0.000 |
| TXNDC16 | -1.539 | 0.000 | 1.132 | 0.000 |
| DNM1L | -1.469 | 0.000 | 1.372 | 0.000 |
| ERGIC2 | -1.730 | 0.000 | 1.420 | 0.000 |
| DDX18 | -1.017 | 0.000 | 1.060 | 0.000 |
| FER1L4 | -2.032 | 0.049 | 2.462 | 0.004 |
| TMEM40 | 1.544 | 0.001 | -1.154 | 0.004 |
| KIZ | -1.062 | 0.000 | 1.545 | 0.000 |
| ESF1 | -4.805 | 0.000 | 5.031 | 0.000 |
| RPLP0 | 1.002 | 0.000 | -1.134 | 0.000 |
| KIF16B | -1.095 | 0.000 | 1.403 | 0.000 |
| ZNF302 | -1.183 | 0.001 | 1.418 | 0.000 |
| CMTM1 | -2.515 | 0.000 | 2.835 | 0.000 |
| RBM41 | -1.778 | 0.000 | 1.601 | 0.000 |
| OTUB2 | 1.895 | 0.000 | -1.495 | 0.001 |
| GPATCH2L | -1.141 | 0.000 | 1.278 | 0.000 |
| BLVRB | 1.723 | 0.000 | -1.400 | 0.000 |
| PAPOLA | -1.269 | 0.000 | 1.018 | 0.000 |
| NDUFB2 | 1.001 | 0.000 | -1.020 | 0.000 |
| ZNF268 | -2.490 | 0.000 | 2.839 | 0.000 |
| CERS4 | 1.283 | 0.000 | -1.108 | 0.000 |
| USP48 | -1.808 | 0.000 | 1.363 | 0.000 |
| KIF4A | -1.908 | 0.000 | 1.476 | 0.000 |
| TNRC6A | -2.035 | 0.000 | 2.374 | 0.000 |
| RBM27 | -2.084 | 0.000 | 1.718 | 0.000 |
| OSBPL8 | -2.416 | 0.000 | 2.327 | 0.000 |
| DTX2 | 1.294 | 0.000 | -1.195 | 0.000 |
| PUS7 | -1.315 | 0.000 | 1.380 | 0.000 |
| LAMB1 | -1.605 | 0.000 | 1.724 | 0.000 |
| ITGA6 | -1.733 | 0.000 | 1.158 | 0.000 |
| ZFHX4 | -1.844 | 0.000 | 1.498 | 0.000 |
| CCDC80 | -1.808 | 0.000 | 1.123 | 0.000 |
| G2E3 | -1.647 | 0.000 | 1.259 | 0.000 |
| HECTD1 | -1.476 | 0.000 | 1.579 | 0.000 |
| SUPT16H | -1.949 | 0.000 | 1.664 | 0.000 |
| TRPM7 | -2.446 | 0.000 | 2.263 | 0.000 |
| CLSPN | -2.752 | 0.000 | 2.727 | 0.000 |
| TGFB2 | -1.353 | 0.000 | 1.153 | 0.000 |
| SUCO | -1.471 | 0.000 | 1.778 | 0.000 |
| MAP3K1 | -2.072 | 0.000 | 1.518 | 0.000 |
| DHPS | -2.280 | 0.004 | 2.168 | 0.003 |
| SH2D3C | 3.189 | 0.010 | -2.795 | 0.007 |
| BTAF1 | -1.344 | 0.000 | 1.477 | 0.000 |
| HIVEP1 | -1.918 | 0.000 | 2.086 | 0.000 |
| CDC5L | -2.774 | 0.000 | 2.460 | 0.000 |
| DSP | -2.034 | 0.000 | 1.664 | 0.000 |
| IFT74 | -3.580 | 0.000 | 3.608 | 0.000 |
| JAK2 | -1.055 | 0.001 | 1.280 | 0.000 |
| SYDE2 | -1.991 | 0.000 | 1.999 | 0.000 |
| PALMD | -2.215 | 0.000 | 1.924 | 0.000 |
| DERL3 | 1.613 | 0.042 | -1.950 | 0.005 |
| LRP5L | -1.348 | 0.004 | 1.481 | 0.001 |
| PLA2G3 | 1.410 | 0.000 | -3.692 | 0.000 |
| LGALS1 | 1.202 | 0.000 | -1.421 | 0.000 |
| CENPM | 1.085 | 0.000 | -1.133 | 0.000 |
| DDX17 | -1.245 | 0.000 | 1.026 | 0.000 |
| NEFH | -1.695 | 0.000 | 1.332 | 0.000 |
| BIK | 3.371 | 0.000 | -2.564 | 0.000 |
| HMOX1 | 2.380 | 0.000 | -1.806 | 0.000 |
| TSPO | 1.139 | 0.000 | -1.176 | 0.000 |
| TNRC6B | -2.087 | 0.000 | 1.488 | 0.000 |
| IL2RB | 3.341 | 0.005 | -1.803 | 0.043 |
| EP300 | -1.192 | 0.000 | 1.050 | 0.000 |
| SOS2 | -1.337 | 0.000 | 1.561 | 0.000 |
| CDKL1 | -1.539 | 0.000 | 1.314 | 0.000 |
| NIN | -2.834 | 0.000 | 3.188 | 0.000 |
| KIAA0586 | -2.907 | 0.000 | 2.660 | 0.000 |
| DAAM1 | -2.369 | 0.000 | 2.278 | 0.000 |
| CEP128 | -1.781 | 0.000 | 2.305 | 0.000 |
| HIF1A | -1.613 | 0.000 | 1.162 | 0.000 |
| DICER1 | -1.819 | 0.000 | 2.236 | 0.000 |
| ZC3H14 | -1.124 | 0.000 | 1.053 | 0.000 |
| CCNB1IP1 | 1.186 | 0.000 | -1.165 | 0.000 |
| TRIP11 | -3.064 | 0.000 | 3.056 | 0.000 |
| ARHGAP5 | -2.549 | 0.000 | 2.720 | 0.000 |
| PNN | -4.744 | 0.000 | 4.284 | 0.000 |
| PLTP | 2.042 | 0.000 | -1.512 | 0.000 |
| HNF4A | 2.238 | 0.003 | -1.307 | 0.030 |
| EEF1A2 | 1.085 | 0.000 | -1.153 | 0.000 |
| TRIB3 | 3.610 | 0.000 | -1.247 | 0.000 |
| PLCB4 | -3.354 | 0.000 | 3.572 | 0.000 |
| NOP56 | -1.086 | 0.000 | 1.017 | 0.000 |
| SMCHD1 | -2.549 | 0.000 | 2.537 | 0.000 |
| CEP192 | -1.839 | 0.000 | 1.358 | 0.000 |
| LIPG | 3.832 | 0.002 | -2.760 | 0.010 |
| ANKRD12 | -3.596 | 0.000 | 3.138 | 0.000 |
| POLI | -1.606 | 0.000 | 1.820 | 0.000 |
| RBBP8 | -2.014 | 0.000 | 2.557 | 0.000 |
| POLA1 | -1.388 | 0.000 | 1.218 | 0.000 |
| NKAP | -1.865 | 0.000 | 2.292 | 0.000 |
| STAG2 | -1.594 | 0.000 | 1.057 | 0.000 |
| ATP11C | -1.545 | 0.000 | 1.442 | 0.000 |
| MCF2 | -1.305 | 0.011 | 1.295 | 0.005 |
| RENBP | 2.624 | 0.029 | -4.354 | 0.002 |
| SMARCA1 | -1.846 | 0.000 | 1.506 | 0.000 |
| ZC3H12B | -3.476 | 0.011 | 3.312 | 0.011 |
| FMR1 | -1.641 | 0.000 | 1.040 | 0.000 |
| EEA1 | -3.965 | 0.000 | 4.074 | 0.000 |
| HTATSF1 | -1.499 | 0.000 | 1.337 | 0.000 |
| GABRE | -2.251 | 0.000 | 1.742 | 0.000 |
| DRP2 | -2.085 | 0.027 | 2.575 | 0.001 |
| FNDC3A | -1.360 | 0.000 | 2.300 | 0.000 |
| UGGT2 | -1.775 | 0.000 | 1.729 | 0.000 |
| PARP4 | -1.425 | 0.000 | 1.152 | 0.000 |
| VWA8 | -1.268 | 0.000 | 1.182 | 0.000 |
| DGKH | -1.897 | 0.000 | 1.936 | 0.000 |
| NFAT5 | -1.994 | 0.000 | 1.991 | 0.000 |
| PARD6A | 1.362 | 0.001 | -1.141 | 0.002 |
| SLC7A6 | -1.319 | 0.000 | 1.003 | 0.000 |
| NECAB2 | 4.109 | 0.000 | -1.156 | 0.047 |
| RPGRIP1L | -2.243 | 0.000 | 1.797 | 0.000 |
| IL21R | 5.947 | 0.000 | -3.641 | 0.000 |
| CCP110 | -2.515 | 0.000 | 2.734 | 0.000 |
| HERC1 | -1.863 | 0.000 | 1.676 | 0.000 |
| ZNF106 | -1.715 | 0.000 | 1.722 | 0.000 |
| CEP152 | -4.834 | 0.000 | 4.924 | 0.000 |
| TJP1 | -1.440 | 0.000 | 1.893 | 0.000 |
| DMXL2 | -2.089 | 0.000 | 2.651 | 0.000 |
| EIF3J | -1.248 | 0.000 | 1.103 | 0.000 |
| CSPP1 | -3.533 | 0.000 | 3.340 | 0.000 |
| NBN | -2.873 | 0.000 | 2.603 | 0.000 |
| UBR5 | -1.581 | 0.000 | 1.422 | 0.000 |
| PEX11G | 1.985 | 0.000 | -1.198 | 0.002 |
| CD37 | -1.036 | 0.014 | 1.216 | 0.000 |
| FCER2 | 5.686 | 0.000 | -1.736 | 0.026 |
| CNTD2 | 3.210 | 0.000 | -2.864 | 0.000 |
| RABAC1 | 1.508 | 0.000 | -1.409 | 0.000 |
| PTPRS | -1.424 | 0.000 | 1.060 | 0.000 |
| DBP | -1.114 | 0.000 | -1.861 | 0.000 |
| TMEM205 | 1.193 | 0.000 | -1.258 | 0.000 |
| FGF21 | 8.297 | 0.000 | -1.837 | 0.000 |
| RPL18A | 1.075 | 0.000 | -1.321 | 0.000 |
| CDK6 | -1.990 | 0.000 | 2.290 | 0.000 |
| DNAJC2 | -4.775 | 0.000 | 4.641 | 0.000 |
| TWISTNB | -1.268 | 0.000 | 1.069 | 0.000 |
| ITGB8 | -2.315 | 0.000 | 1.651 | 0.000 |
| DNAH11 | -1.841 | 0.000 | 1.708 | 0.000 |
| MET | -1.165 | 0.000 | 1.073 | 0.000 |
| TAX1BP1 | -3.073 | 0.000 | 3.016 | 0.000 |
| ZKSCAN1 | -1.381 | 0.000 | 1.527 | 0.000 |
| SNX8 | 1.044 | 0.000 | -1.184 | 0.000 |
| NUDT1 | 1.019 | 0.000 | -1.356 | 0.000 |
| RBM28 | -1.439 | 0.000 | 1.322 | 0.000 |
| PHF14 | -2.652 | 0.000 | 2.641 | 0.000 |
| SEC61B | 1.003 | 0.000 | -1.163 | 0.000 |
| MPDZ | -2.138 | 0.000 | 2.080 | 0.000 |
| EDF1 | 1.080 | 0.000 | -1.036 | 0.000 |
| SETX | -2.608 | 0.000 | 2.893 | 0.000 |
| RAB11FIP2 | -1.182 | 0.000 | 1.498 | 0.000 |
| EIF3A | -2.831 | 0.000 | 2.807 | 0.000 |
| ARHGAP21 | -3.254 | 0.000 | 2.791 | 0.000 |
| ANKRD26 | -4.593 | 0.000 | 4.749 | 0.000 |
| FAM208B | -2.078 | 0.000 | 2.528 | 0.000 |
| SMC3 | -4.434 | 0.000 | 3.979 | 0.000 |
| CUL2 | -1.721 | 0.000 | 1.346 | 0.000 |
| NUFIP2 | -2.196 | 0.000 | 1.491 | 0.000 |
| KPNB1 | -1.679 | 0.000 | 1.293 | 0.000 |
| RPS6KB1 | -1.715 | 0.000 | 1.609 | 0.000 |
| TRIM16L | 1.554 | 0.000 | -1.344 | 0.000 |
| CDK5RAP3 | -2.266 | 0.000 | 2.304 | 0.000 |
| GALK1 | 1.175 | 0.000 | -1.029 | 0.000 |
| INTS2 | -1.417 | 0.000 | 1.322 | 0.000 |
| MED13 | -1.549 | 0.000 | 1.554 | 0.000 |
| CCDC47 | -1.165 | 0.000 | 1.009 | 0.000 |
| DDX5 | -1.338 | 0.000 | 1.218 | 0.000 |
| VAT1 | 1.046 | 0.000 | -1.175 | 0.000 |
| LUC7L3 | -5.443 | 0.000 | 4.885 | 0.000 |
| ALDOC | 1.003 | 0.000 | -1.161 | 0.000 |
| FBXW7 | -1.341 | 0.000 | 1.527 | 0.000 |
| STIM2 | -1.252 | 0.000 | 1.323 | 0.000 |
| RAPGEF2 | -1.539 | 0.000 | 1.771 | 0.000 |
| NCAPG | -2.563 | 0.000 | 2.016 | 0.000 |
| CCDC34 | -3.042 | 0.000 | 2.817 | 0.000 |
| ZBTB16 | -3.460 | 0.000 | 2.290 | 0.001 |
| FNBP4 | -1.603 | 0.000 | 1.508 | 0.000 |
| SIAE | -1.234 | 0.000 | 1.075 | 0.000 |
| PPP6R3 | -1.413 | 0.000 | 1.137 | 0.000 |
| CEP126 | -3.265 | 0.000 | 3.274 | 0.000 |
| EXPH5 | -3.356 | 0.000 | 2.494 | 0.000 |
| CAPRIN2 | -1.020 | 0.000 | 1.973 | 0.000 |
| MVK | 1.381 | 0.000 | -2.011 | 0.000 |
| RSRC2 | -3.320 | 0.000 | 3.270 | 0.000 |
| CYP27B1 | -1.682 | 0.000 | 1.773 | 0.000 |
| METAP2 | -1.796 | 0.000 | 1.353 | 0.000 |
| DDX55 | -1.382 | 0.000 | 1.236 | 0.000 |
| KRR1 | -2.841 | 0.000 | 2.709 | 0.000 |
| UHRF1BP1L | -1.711 | 0.000 | 1.484 | 0.000 |
| GNPTAB | -2.020 | 0.000 | 2.087 | 0.000 |
| NT5DC3 | -1.139 | 0.000 | 1.036 | 0.000 |
| AC009533.1 | -1.229 | 0.000 | 1.102 | 0.000 |
| COL12A1 | -2.068 | 0.000 | 1.934 | 0.000 |
| PAK1IP1 | -1.057 | 0.000 | 1.243 | 0.000 |
| CEP85L | -1.510 | 0.000 | 1.994 | 0.000 |
| ULBP1 | 2.052 | 0.000 | 1.523 | 0.000 |
| ICK | -1.390 | 0.000 | 1.434 | 0.000 |
| MDN1 | -1.978 | 0.000 | 1.747 | 0.000 |
| ASCC3 | -1.732 | 0.000 | 1.601 | 0.000 |
| HECA | -1.292 | 0.000 | 1.331 | 0.000 |
| PHACTR2 | -1.307 | 0.000 | 1.881 | 0.000 |
| PDE10A | -1.238 | 0.000 | 1.035 | 0.000 |
| SENP6 | -2.629 | 0.000 | 2.847 | 0.000 |
| PRPF4B | -4.082 | 0.000 | 3.403 | 0.000 |
| TTK | -2.474 | 0.000 | 1.743 | 0.000 |
| TENT5A | -2.492 | 0.000 | 1.213 | 0.000 |
| PRSS16 | 2.105 | 0.000 | -1.046 | 0.002 |
| ERBIN | -1.783 | 0.000 | 2.092 | 0.000 |
| HMGCS1 | 1.023 | 0.000 | -1.679 | 0.000 |
| IK | -2.053 | 0.000 | 1.573 | 0.000 |
| COL4A3BP | -1.403 | 0.000 | 1.124 | 0.000 |
| MSH3 | -1.380 | 0.000 | 1.233 | 0.000 |
| PDE4D | -1.647 | 0.000 | 1.137 | 0.000 |
| BRIX1 | -1.541 | 0.000 | 1.318 | 0.000 |
| RAD50 | -2.973 | 0.000 | 3.301 | 0.000 |
| LIFR | -1.301 | 0.000 | 1.347 | 0.000 |
| TCERG1 | -2.652 | 0.000 | 2.347 | 0.000 |
| SMC4 | -5.109 | 0.000 | 4.300 | 0.000 |
| UBE3A | -1.160 | 0.000 | 1.127 | 0.000 |
| CEP70 | -2.377 | 0.000 | 1.926 | 0.000 |
| XRN1 | -2.297 | 0.000 | 2.208 | 0.000 |
| COL7A1 | -1.322 | 0.001 | 1.350 | 0.000 |
| ECT2 | -2.112 | 0.000 | 1.458 | 0.000 |
| BBX | -2.541 | 0.000 | 2.628 | 0.000 |
| IQCG | -1.490 | 0.000 | 2.081 | 0.000 |
| FRMD4B | -1.385 | 0.016 | 1.595 | 0.001 |
| TNNC1 | 1.215 | 0.000 | -1.195 | 0.000 |
| NKTR | -4.721 | 0.000 | 4.292 | 0.000 |
| FOXP1 | -1.199 | 0.000 | 1.006 | 0.000 |
| INO80D | -1.157 | 0.000 | 1.135 | 0.000 |
| PIKFYVE | -2.125 | 0.000 | 1.832 | 0.000 |
| FAHD2A | 1.107 | 0.000 | -1.003 | 0.000 |
| NCL | -2.216 | 0.000 | 1.874 | 0.000 |
| DNAJC27 | -1.505 | 0.002 | 1.553 | 0.000 |
| FNDC4 | 2.400 | 0.000 | -1.311 | 0.000 |
| IFIH1 | -2.212 | 0.000 | 1.729 | 0.000 |
| SPTBN1 | -1.591 | 0.000 | 1.220 | 0.000 |
| CCDC88A | -4.060 | 0.000 | 4.051 | 0.000 |
| USP34 | -2.233 | 0.000 | 1.925 | 0.000 |
| EHBP1 | -2.455 | 0.000 | 2.554 | 0.000 |
| SLC9A2 | 3.337 | 0.000 | 1.177 | 0.000 |
| ID2 | -3.853 | 0.000 | 1.945 | 0.000 |
| TAF1B | -1.164 | 0.000 | 1.107 | 0.000 |
| BIRC6 | -2.648 | 0.000 | 2.175 | 0.000 |
| NOL10 | -1.527 | 0.000 | 1.321 | 0.000 |
| STRN | -1.168 | 0.000 | 1.078 | 0.000 |
| CEBPZ | -4.050 | 0.000 | 3.644 | 0.000 |
| SOS1 | -1.601 | 0.000 | 2.019 | 0.000 |
| COX7A2L | 1.428 | 0.000 | -1.213 | 0.000 |
| ALMS1 | -2.520 | 0.000 | 2.800 | 0.000 |
| STXBP3 | -2.155 | 0.000 | 1.721 | 0.000 |
| EDEM3 | -1.476 | 0.000 | 1.567 | 0.000 |
| ASH1L | -2.587 | 0.000 | 2.399 | 0.000 |
| GON4L | -1.160 | 0.000 | 1.084 | 0.000 |
| FBXO2 | 1.469 | 0.000 | -2.785 | 0.000 |
| SWT1 | -1.173 | 0.018 | 1.041 | 0.025 |
| PRDM2 | -1.158 | 0.000 | 1.355 | 0.000 |
| TROVE2 | -1.087 | 0.000 | 1.037 | 0.000 |
| SRSF11 | -3.161 | 0.000 | 3.008 | 0.000 |
| TFAP2E | -2.485 | 0.049 | 2.572 | 0.018 |
| EXOC8 | -1.579 | 0.000 | 1.218 | 0.000 |
| MTR | -2.493 | 0.000 | 2.630 | 0.000 |
| RLF | -2.131 | 0.000 | 1.942 | 0.000 |
| SSX2IP | -1.294 | 0.000 | 1.490 | 0.000 |
| ZNHIT6 | -1.022 | 0.000 | 1.298 | 0.000 |
| ATP6V0B | 1.169 | 0.000 | -1.155 | 0.000 |
| AKR1A1 | 1.225 | 0.000 | -1.278 | 0.000 |
| TSPAN1 | 1.378 | 0.002 | -1.677 | 0.000 |
| PRRC2C | -1.902 | 0.000 | 1.653 | 0.000 |
| RSRP1 | -1.375 | 0.000 | 1.332 | 0.000 |
| CENPF | -5.543 | 0.000 | 4.677 | 0.000 |
| CTSD | 1.488 | 0.000 | -1.732 | 0.000 |
| KMT2A | -1.287 | 0.000 | 1.182 | 0.000 |
| KIF14 | -4.832 | 0.000 | 3.983 | 0.000 |
| CAMSAP2 | -2.412 | 0.000 | 2.317 | 0.000 |
| KLF7 | -1.564 | 0.000 | 1.484 | 0.000 |
| FILIP1 | -3.678 | 0.000 | 2.886 | 0.000 |
| CASP8AP2 | -3.594 | 0.000 | 3.715 | 0.000 |
| PHF3 | -4.308 | 0.000 | 4.195 | 0.000 |
| RAB32 | 1.523 | 0.000 | -1.142 | 0.000 |
| SLC16A7 | -1.518 | 0.000 | 1.752 | 0.000 |
| ZNF430 | -1.983 | 0.000 | 2.425 | 0.000 |
| RAB3GAP2 | -1.193 | 0.000 | 1.060 | 0.000 |
| HS1BP3 | 1.588 | 0.000 | -1.249 | 0.000 |
| WDR35 | -1.306 | 0.000 | 1.205 | 0.000 |
| KLF9 | -2.958 | 0.000 | 1.526 | 0.000 |
| FKBP15 | -1.212 | 0.000 | 1.197 | 0.000 |
| CNTRL | -3.685 | 0.000 | 3.884 | 0.000 |
| NR4A3 | -1.660 | 0.000 | 1.378 | 0.000 |
| C19orf25 | 1.319 | 0.000 | -1.147 | 0.000 |
| BBOF1 | -1.823 | 0.010 | 1.998 | 0.001 |
| MLH3 | -1.047 | 0.000 | 1.466 | 0.000 |
| RBM25 | -3.893 | 0.000 | 3.716 | 0.000 |
| GPR75 | -1.212 | 0.037 | 1.661 | 0.000 |
| ATAD2B | -1.541 | 0.000 | 1.756 | 0.000 |
| HELLS | -1.759 | 0.000 | 1.476 | 0.000 |
| KANSL1 | -1.234 | 0.000 | 1.193 | 0.000 |
| HOXB3 | -2.517 | 0.000 | 1.651 | 0.011 |
| MLANA | -3.435 | 0.011 | 2.912 | 0.042 |
| CYSTM1 | 1.420 | 0.000 | -1.063 | 0.000 |
| GORAB | -1.657 | 0.000 | 1.411 | 0.000 |
| PLXDC2 | -1.285 | 0.000 | 1.474 | 0.000 |
| CCDC77 | -1.445 | 0.000 | 1.289 | 0.000 |
| WBP4 | -2.852 | 0.000 | 2.869 | 0.000 |
| SMAD9 | -1.401 | 0.000 | 1.216 | 0.000 |
| HSPH1 | -1.847 | 0.000 | 1.646 | 0.000 |
| ZFP30 | -1.651 | 0.003 | 1.254 | 0.015 |
| UTP20 | -2.558 | 0.000 | 2.185 | 0.000 |
| TMEM131L | -1.325 | 0.000 | 1.105 | 0.000 |
| ECHDC2 | -1.569 | 0.000 | 1.325 | 0.000 |
| PYROXD1 | -1.608 | 0.000 | 2.242 | 0.000 |
| KIF18A | -4.500 | 0.000 | 3.886 | 0.000 |
| PILRB | -2.993 | 0.000 | 2.698 | 0.000 |
| ZMYM2 | -1.380 | 0.000 | 1.449 | 0.000 |
| TNFSF10 | -5.551 | 0.000 | 1.154 | 0.043 |
| PIK3CA | -1.815 | 0.000 | 1.893 | 0.000 |
| PDS5A | -1.681 | 0.000 | 1.528 | 0.000 |
| TMEM156 | 2.330 | 0.006 | -1.276 | 0.049 |
| TMEM54 | 2.187 | 0.000 | -1.152 | 0.000 |
| POLK | -2.134 | 0.000 | 2.391 | 0.000 |
| RBBP6 | -2.282 | 0.000 | 1.902 | 0.000 |
| PRXL2A | 1.085 | 0.000 | -1.332 | 0.000 |
| ODF2L | -3.412 | 0.000 | 3.450 | 0.000 |
| TRMT13 | -1.192 | 0.000 | 1.264 | 0.000 |
| ZNF644 | -2.754 | 0.000 | 2.492 | 0.000 |
| CCDC18 | -4.666 | 0.000 | 4.300 | 0.000 |
| RPAP2 | -1.550 | 0.000 | 1.541 | 0.000 |
| SEPT7 | -1.961 | 0.000 | 1.597 | 0.000 |
| TRIM24 | -2.288 | 0.000 | 2.294 | 0.000 |
| CALD1 | -2.579 | 0.000 | 2.358 | 0.000 |
| BICC1 | -1.493 | 0.000 | 1.654 | 0.000 |
| CIT | -1.784 | 0.000 | 1.374 | 0.000 |
| IFT81 | -3.155 | 0.000 | 2.719 | 0.000 |
| MED13L | -1.475 | 0.000 | 1.631 | 0.000 |
| RASSF8 | -2.065 | 0.000 | 2.128 | 0.000 |
| ITPR2 | -2.668 | 0.000 | 2.685 | 0.000 |
| CCDC91 | -2.425 | 0.000 | 2.471 | 0.000 |
| ZC3H13 | -3.132 | 0.000 | 2.956 | 0.000 |
| MMP19 | 2.476 | 0.000 | -1.096 | 0.043 |
| ATG101 | 1.188 | 0.000 | -1.071 | 0.000 |
| BAZ2B | -4.113 | 0.000 | 4.077 | 0.000 |
| KCNJ2 | -1.107 | 0.031 | 1.592 | 0.000 |
| PARD6B | -1.814 | 0.000 | 1.438 | 0.000 |
| CHD6 | -2.027 | 0.000 | 1.858 | 0.000 |
| SRSF6 | -1.272 | 0.000 | 1.152 | 0.000 |
| KCNK15 | 1.429 | 0.000 | -1.257 | 0.000 |
| MPHOSPH10 | -4.798 | 0.000 | 4.418 | 0.000 |
| POF1B | -2.419 | 0.001 | 1.688 | 0.034 |
| USP9X | -1.764 | 0.000 | 1.464 | 0.000 |
| CDKN1A | 2.281 | 0.000 | -1.130 | 0.000 |
| RIOK1 | -1.174 | 0.000 | 1.550 | 0.000 |
| DEK | -3.241 | 0.000 | 2.786 | 0.000 |
| LRRFIP1 | -2.756 | 0.000 | 2.679 | 0.000 |
| AHNAK | -1.961 | 0.000 | 1.455 | 0.000 |
| SSUH2 | -1.980 | 0.008 | 2.191 | 0.000 |
| UPF3B | -3.980 | 0.000 | 3.869 | 0.000 |
| NT5C | 1.187 | 0.000 | -1.322 | 0.000 |
| TTF1 | -2.094 | 0.000 | 1.851 | 0.000 |
| CHCHD5 | 1.327 | 0.000 | -1.178 | 0.000 |
| CCDC93 | -1.532 | 0.000 | 1.796 | 0.000 |
| ALKBH7 | 1.056 | 0.000 | -1.299 | 0.000 |
| THOC2 | -4.957 | 0.000 | 4.257 | 0.000 |
| MCM8 | -1.712 | 0.000 | 1.806 | 0.000 |
| ID1 | -2.755 | 0.000 | 1.465 | 0.000 |
| AGO3 | -1.120 | 0.000 | 1.455 | 0.000 |
| PRDX5 | 1.270 | 0.000 | -1.366 | 0.000 |
| NSRP1 | -3.739 | 0.000 | 3.836 | 0.000 |
| TIMM17B | 1.041 | 0.000 | -1.178 | 0.000 |
| KTN1 | -5.025 | 0.000 | 4.620 | 0.000 |
| DLGAP5 | -4.031 | 0.000 | 3.053 | 0.000 |
| PZP | -4.042 | 0.000 | 1.402 | 0.000 |
| WDR60 | -3.056 | 0.000 | 2.292 | 0.000 |
| ZNF484 | -2.016 | 0.000 | 2.083 | 0.000 |
| PTPRB | -1.757 | 0.000 | 1.250 | 0.004 |
| LRRC61 | 1.410 | 0.000 | -1.101 | 0.000 |
| HP1BP3 | -1.977 | 0.000 | 1.055 | 0.000 |
| F2RL3 | 2.754 | 0.000 | -1.275 | 0.000 |
| MACF1 | -1.573 | 0.000 | 1.638 | 0.000 |
| RNF6 | -1.520 | 0.000 | 1.994 | 0.000 |
| AKAP9 | -4.029 | 0.000 | 3.871 | 0.000 |
| GNGT1 | -2.212 | 0.000 | 2.763 | 0.000 |
| DGCR6L | 1.193 | 0.000 | -1.235 | 0.000 |
| SDF2L1 | 1.664 | 0.000 | -1.616 | 0.000 |
| ATF4 | 1.085 | 0.000 | -2.382 | 0.000 |
| TST | 1.224 | 0.000 | -1.056 | 0.000 |
| KRT17 | 2.266 | 0.000 | -1.445 | 0.000 |
| FOXP2 | -1.589 | 0.000 | 1.537 | 0.000 |
| DNAJB9 | 2.161 | 0.000 | -1.323 | 0.000 |
| VPS13C | -3.542 | 0.000 | 3.494 | 0.000 |
| PHF20L1 | -2.081 | 0.000 | 2.395 | 0.000 |
| PUS7L | -1.978 | 0.000 | 1.792 | 0.000 |
| MTUS1 | -2.786 | 0.000 | 2.151 | 0.000 |
| MIS18BP1 | -5.176 | 0.000 | 4.219 | 0.000 |
| DAD1 | 1.202 | 0.000 | -1.163 | 0.000 |
| MAP7D3 | -3.906 | 0.000 | 3.779 | 0.000 |
| SGO1 | -1.468 | 0.000 | 1.368 | 0.000 |
| GAMT | 1.090 | 0.000 | -1.096 | 0.000 |
| RPL36 | 1.076 | 0.000 | -1.195 | 0.000 |
| PGLS | 1.342 | 0.000 | -1.413 | 0.000 |
| ZSWIM6 | -1.333 | 0.000 | 1.039 | 0.000 |
| KLHDC7B | 7.844 | 0.000 | -1.757 | 0.000 |
| GDF15 | 4.156 | 0.000 | -1.156 | 0.000 |
| PGPEP1 | 1.742 | 0.000 | -1.015 | 0.000 |
| ZNF557 | -1.051 | 0.000 | 1.372 | 0.000 |
| LAMA5 | -1.942 | 0.000 | 1.044 | 0.000 |
| ASS1 | 1.622 | 0.000 | -1.057 | 0.000 |
| TMEM160 | 1.232 | 0.000 | -1.341 | 0.000 |
| SESN2 | 3.335 | 0.000 | -1.099 | 0.000 |
| CLIP1 | -3.877 | 0.000 | 3.606 | 0.000 |
| ZNF331 | -2.002 | 0.000 | 1.817 | 0.000 |
| POLN | -1.518 | 0.025 | 1.710 | 0.003 |
| PPIL4 | -2.142 | 0.000 | 2.066 | 0.000 |
| AKAP12 | -2.598 | 0.000 | 2.347 | 0.000 |
| ULBP3 | -1.367 | 0.004 | 1.927 | 0.000 |
| RBM39 | -1.605 | 0.000 | 1.653 | 0.000 |
| ACSS2 | 1.864 | 0.000 | -1.128 | 0.000 |
| KIF3A | -2.668 | 0.000 | 2.932 | 0.000 |
| PPFIA1 | -1.064 | 0.000 | 1.171 | 0.000 |
| KRT34 | 3.764 | 0.000 | -3.046 | 0.000 |
| TOP2A | -4.396 | 0.000 | 3.603 | 0.000 |
| FBXW9 | -1.521 | 0.000 | 1.016 | 0.001 |
| HSD17B7 | 1.272 | 0.000 | -2.137 | 0.000 |
| PNISR | -3.892 | 0.000 | 3.764 | 0.000 |
| SEC61G | 1.117 | 0.000 | -1.253 | 0.000 |
| ANKRD17 | -2.030 | 0.000 | 1.778 | 0.000 |
| UTP3 | -1.120 | 0.000 | 1.210 | 0.000 |
| VPS13B | -1.875 | 0.000 | 1.847 | 0.000 |
| BTBD3 | -2.055 | 0.000 | 1.132 | 0.000 |
| NASP | -1.251 | 0.000 | 1.107 | 0.000 |
| PATJ | -1.123 | 0.000 | 1.096 | 0.000 |
| MTUS2 | -1.710 | 0.009 | 1.703 | 0.004 |
| MYH10 | -1.842 | 0.000 | 1.888 | 0.000 |
| STARD13 | -1.042 | 0.002 | 1.088 | 0.000 |
| TCEAL4 | -1.770 | 0.000 | 1.802 | 0.000 |
| SRRM1 | -1.803 | 0.000 | 1.324 | 0.000 |
| SLF1 | -3.072 | 0.000 | 1.926 | 0.000 |
| PDZD2 | -3.468 | 0.003 | 3.914 | 0.000 |
| ZNF767P | -1.536 | 0.003 | 1.969 | 0.000 |
| SPINK5 | -3.377 | 0.000 | 1.697 | 0.001 |
| LRRCC1 | -3.211 | 0.000 | 3.767 | 0.000 |
| CCDC59 | -1.281 | 0.000 | 1.501 | 0.000 |
| ZFC3H1 | -3.414 | 0.000 | 3.132 | 0.000 |
| TEX15 | -3.969 | 0.000 | 4.243 | 0.000 |
| EIF2S1 | 2.519 | 0.008 | -2.424 | 0.001 |
| PRPF38B | -4.047 | 0.000 | 3.686 | 0.000 |
| NOTCH2 | -1.243 | 0.000 | 1.115 | 0.000 |
| KIDINS220 | -1.969 | 0.000 | 2.152 | 0.000 |
| ROCK2 | -3.762 | 0.000 | 3.806 | 0.000 |
| IL6ST | -1.300 | 0.000 | 1.472 | 0.000 |
| SPOCD1 | 2.333 | 0.000 | -1.605 | 0.000 |
| GNL2 | -2.915 | 0.000 | 3.056 | 0.000 |
| TUT4 | -2.570 | 0.000 | 2.296 | 0.000 |
| CLOCK | -1.545 | 0.000 | 1.540 | 0.000 |
| ARGLU1 | -2.130 | 0.000 | 2.053 | 0.000 |
| TPP2 | -1.827 | 0.000 | 1.563 | 0.000 |
| APC | -4.586 | 0.000 | 4.247 | 0.000 |
| TAOK3 | -2.462 | 0.000 | 2.375 | 0.000 |
| DMTF1 | -1.080 | 0.000 | 1.716 | 0.000 |
| KCP | 3.929 | 0.002 | -4.073 | 0.000 |
| CEP162 | -5.796 | 0.000 | 5.379 | 0.000 |
| LCA5 | -2.523 | 0.000 | 2.937 | 0.000 |
| RDH5 | -3.050 | 0.043 | 3.168 | 0.020 |
| CDK4 | 1.055 | 0.000 | -1.043 | 0.000 |
| LTV1 | -2.660 | 0.000 | 2.671 | 0.000 |
| AHI1 | -3.431 | 0.000 | 3.749 | 0.000 |
| SMPD2 | 1.037 | 0.000 | -1.073 | 0.000 |
| CCDC142 | -1.202 | 0.000 | 1.031 | 0.001 |
| USP15 | -1.800 | 0.000 | 1.535 | 0.000 |
| AGT | 2.429 | 0.018 | -1.489 | 0.038 |
| CEP350 | -3.239 | 0.000 | 3.097 | 0.000 |
| LAMC1 | -1.267 | 0.000 | 1.247 | 0.000 |
| RC3H1 | -1.965 | 0.000 | 1.585 | 0.000 |
| DOCK10 | -1.638 | 0.000 | 1.913 | 0.000 |
| GCC2 | -4.340 | 0.000 | 4.003 | 0.000 |
| ANKRD36 | -2.260 | 0.000 | 2.581 | 0.000 |
| EPC2 | -1.120 | 0.000 | 1.213 | 0.000 |
| WASHC4 | -1.102 | 0.000 | 1.094 | 0.000 |
| CKAP2 | -3.281 | 0.000 | 2.543 | 0.000 |
| LMO7 | -2.435 | 0.000 | 1.422 | 0.000 |
| SETDB2 | -1.026 | 0.000 | 1.185 | 0.000 |
| CIB2 | 1.501 | 0.000 | -1.063 | 0.000 |
| BRIP1 | -1.969 | 0.000 | 1.832 | 0.000 |
| SKIL | -2.675 | 0.000 | 2.148 | 0.000 |
| EPRS | -1.494 | 0.000 | 2.110 | 0.000 |
| GYPC | 1.769 | 0.000 | -1.179 | 0.000 |
| SMC2 | -4.508 | 0.000 | 3.990 | 0.000 |
| CDK5RAP2 | -1.393 | 0.000 | 1.384 | 0.000 |
| ZFP37 | -3.179 | 0.000 | 2.497 | 0.001 |
| DPM2 | 1.563 | 0.000 | -1.024 | 0.000 |
| GOLGA1 | -1.134 | 0.000 | 1.348 | 0.000 |
| RPL35 | 1.025 | 0.000 | -1.134 | 0.000 |
| RANBP6 | -1.175 | 0.000 | 1.100 | 0.000 |
| KIF13A | -1.903 | 0.000 | 1.039 | 0.000 |
| TUBB2B | 3.374 | 0.014 | -2.116 | 0.041 |
| IL18BP | -1.356 | 0.020 | 1.363 | 0.003 |
| SYTL2 | -1.138 | 0.034 | 2.165 | 0.000 |
| TGS1 | -2.046 | 0.000 | 1.842 | 0.000 |
| NEK1 | -2.713 | 0.000 | 2.738 | 0.000 |
| DDX60 | -1.729 | 0.006 | 2.578 | 0.000 |
| SORL1 | -1.092 | 0.000 | 1.076 | 0.000 |
| RDX | -3.540 | 0.000 | 2.668 | 0.000 |
| SLTM | -2.942 | 0.000 | 3.164 | 0.000 |
| THBS1 | -1.847 | 0.000 | 2.213 | 0.000 |
| KIF23 | -2.486 | 0.000 | 2.015 | 0.000 |
| KNL1 | -4.513 | 0.000 | 3.641 | 0.000 |
| RTF1 | -2.522 | 0.000 | 1.920 | 0.000 |
| UACA | -4.783 | 0.000 | 4.546 | 0.000 |
| ADAM10 | -1.701 | 0.000 | 1.433 | 0.000 |
| SEMA6D | -2.962 | 0.000 | 1.537 | 0.000 |
| SPTBN5 | -3.053 | 0.022 | 2.956 | 0.020 |
| TTLL7 | -1.680 | 0.000 | 1.954 | 0.000 |
| FNBP1L | -1.416 | 0.000 | 1.472 | 0.000 |
| IFI44 | -2.888 | 0.001 | 2.329 | 0.008 |
| KIF11 | -3.287 | 0.000 | 2.352 | 0.000 |
| CEP55 | -2.162 | 0.000 | 1.806 | 0.000 |
| KIF20B | -5.616 | 0.000 | 4.638 | 0.000 |
| PLCE1 | -1.689 | 0.000 | 2.287 | 0.000 |
| DNAJC13 | -2.116 | 0.000 | 1.946 | 0.000 |
| TET1 | -7.873 | 0.000 | 5.519 | 0.000 |
| SSB | -3.937 | 0.000 | 3.866 | 0.000 |
| PPIG | -4.969 | 0.000 | 4.724 | 0.000 |
| CIR1 | -4.236 | 0.000 | 3.549 | 0.000 |
| SENP7 | -1.686 | 0.000 | 1.083 | 0.003 |
| MNS1 | -4.248 | 0.000 | 4.335 | 0.000 |
| USP8 | -3.707 | 0.000 | 3.752 | 0.000 |
| SECISBP2L | -2.307 | 0.000 | 1.768 | 0.000 |
| ZGRF1 | -3.814 | 0.000 | 3.684 | 0.000 |
| KIAA1109 | -3.494 | 0.000 | 3.358 | 0.000 |
| USO1 | -2.194 | 0.000 | 2.226 | 0.000 |
| CENPE | -6.520 | 0.000 | 5.895 | 0.000 |
| FBN2 | -1.197 | 0.000 | 1.067 | 0.000 |
| MAPK8IP3 | -1.555 | 0.000 | 1.162 | 0.000 |
| RNF185 | 1.453 | 0.000 | -1.021 | 0.000 |
| KIF21A | -3.183 | 0.000 | 3.412 | 0.000 |
| FGD4 | -1.264 | 0.000 | 1.396 | 0.000 |
| SLCO1C1 | -3.878 | 0.002 | 3.064 | 0.028 |
| CLSTN3 | -1.562 | 0.000 | 1.238 | 0.000 |
| VAMP1 | -1.522 | 0.000 | 1.762 | 0.000 |
| RBP5 | -1.856 | 0.010 | 1.672 | 0.004 |
| SCAF11 | -2.766 | 0.000 | 2.429 | 0.000 |
| LLPH | -1.258 | 0.000 | 1.012 | 0.000 |
| MARCH9 | 1.485 | 0.000 | -1.399 | 0.000 |
| INHBE | 4.536 | 0.000 | -1.823 | 0.000 |
| TMTC3 | -2.225 | 0.000 | 2.075 | 0.000 |
| SDSL | 3.424 | 0.000 | -1.140 | 0.000 |
| MMAB | 1.431 | 0.000 | -1.754 | 0.000 |
| BRCA2 | -3.666 | 0.000 | 3.680 | 0.000 |
| CSAD | -1.611 | 0.000 | 1.584 | 0.000 |
| C12orf10 | 1.047 | 0.000 | -1.238 | 0.000 |
| SBNO1 | -2.539 | 0.000 | 2.064 | 0.000 |
| DIAPH3 | -2.536 | 0.000 | 2.397 | 0.000 |
| RBM26 | -2.360 | 0.000 | 2.238 | 0.000 |
| MBNL2 | -1.683 | 0.000 | 1.546 | 0.000 |
| STON2 | -1.076 | 0.000 | 1.063 | 0.000 |
| MFAP1 | -2.014 | 0.000 | 1.660 | 0.000 |
| SCAPER | -2.165 | 0.000 | 2.744 | 0.000 |
| NCOA2 | -1.359 | 0.000 | 1.320 | 0.000 |
| PIF1 | -2.121 | 0.000 | 1.046 | 0.000 |
| CYP1A1 | 2.995 | 0.000 | -1.086 | 0.000 |
| IQGAP1 | -2.113 | 0.000 | 1.608 | 0.000 |
| ZFHX3 | -1.477 | 0.000 | 1.423 | 0.000 |
| OSGIN1 | 3.064 | 0.000 | -1.658 | 0.000 |
| NCOR1 | -1.510 | 0.000 | 1.852 | 0.000 |
| ESCO1 | -3.570 | 0.000 | 3.502 | 0.000 |
| GREB1L | -1.171 | 0.000 | 1.027 | 0.000 |
| ZCCHC2 | -1.665 | 0.000 | 1.318 | 0.000 |
| IGFBP4 | 1.976 | 0.000 | -1.306 | 0.000 |
| TPGS1 | 1.236 | 0.000 | -1.417 | 0.000 |
| MVB12A | 1.287 | 0.000 | -1.245 | 0.000 |
| ZFP14 | -4.250 | 0.035 | 4.102 | 0.036 |
| ADAMTS10 | -1.277 | 0.003 | 1.077 | 0.011 |
| ZNF614 | -1.022 | 0.000 | 1.320 | 0.000 |
| PADI3 | 2.535 | 0.000 | -2.795 | 0.000 |
| PADI1 | 2.204 | 0.013 | -1.689 | 0.023 |
| PLK4 | -1.738 | 0.000 | 1.417 | 0.000 |
| POU2F1 | -1.355 | 0.000 | 1.154 | 0.000 |
| NUF2 | -3.224 | 0.000 | 2.543 | 0.000 |
| HMCN1 | -1.649 | 0.001 | 2.095 | 0.000 |
| CTSK | -2.555 | 0.001 | 1.999 | 0.012 |
| HAX1 | 1.778 | 0.000 | -1.108 | 0.000 |
| CEP170 | -2.561 | 0.000 | 2.729 | 0.000 |
| GUK1 | 1.263 | 0.000 | -1.061 | 0.000 |
| CDC42BPA | -2.180 | 0.000 | 2.862 | 0.000 |
| ETNK2 | 1.247 | 0.000 | -1.020 | 0.000 |
| PLEKHA6 | 2.116 | 0.000 | -1.292 | 0.000 |
| EML4 | -1.309 | 0.000 | 1.242 | 0.000 |
| ASXL2 | -1.293 | 0.000 | 1.134 | 0.000 |
| ETAA1 | -5.279 | 0.000 | 4.184 | 0.000 |
| MEIS1 | -2.302 | 0.000 | 1.624 | 0.000 |
| ZC3H8 | -1.065 | 0.000 | 1.520 | 0.000 |
| UBXN4 | -2.142 | 0.000 | 1.990 | 0.000 |
| SCN1A | -3.817 | 0.000 | 2.022 | 0.000 |
| UBR3 | -1.817 | 0.000 | 1.521 | 0.000 |
| CCDC150 | -3.773 | 0.000 | 3.790 | 0.000 |
| NBEAL1 | -2.251 | 0.000 | 2.075 | 0.000 |
| NYAP2 | -2.325 | 0.000 | 1.101 | 0.011 |
| CPNE9 | 2.788 | 0.006 | -1.691 | 0.026 |
| FANCD2 | -1.480 | 0.000 | 1.190 | 0.000 |
| GMPPA | 1.320 | 0.000 | -1.338 | 0.000 |
| GOLGA4 | -4.260 | 0.000 | 4.129 | 0.000 |
| TMF1 | -3.379 | 0.000 | 2.994 | 0.000 |
| NFKBIZ | -2.047 | 0.000 | 2.555 | 0.000 |
| PHLDB2 | -1.008 | 0.000 | 1.430 | 0.000 |
| LPP | -1.138 | 0.000 | 1.392 | 0.000 |
| MANF | 1.259 | 0.000 | -1.692 | 0.000 |
| FIP1L1 | -1.037 | 0.000 | 1.105 | 0.000 |
| LYAR | -2.405 | 0.000 | 2.532 | 0.000 |
| CENPC | -4.366 | 0.000 | 3.952 | 0.000 |
| EPHA5 | -2.062 | 0.000 | 1.146 | 0.000 |
| ANK2 | 3.663 | 0.000 | 1.982 | 0.000 |
| SPATA5 | -1.225 | 0.000 | 1.060 | 0.000 |
| USP53 | -1.391 | 0.000 | 2.287 | 0.000 |
| MARCH1 | -2.797 | 0.000 | 2.728 | 0.000 |
| PIK3R1 | -1.828 | 0.000 | 1.922 | 0.000 |
| IQGAP2 | -2.571 | 0.000 | 2.638 | 0.000 |
| RASA1 | -1.156 | 0.000 | 1.357 | 0.000 |
| PPIP5K2 | -2.158 | 0.000 | 1.533 | 0.000 |
| BDP1 | -3.997 | 0.000 | 3.864 | 0.000 |
| DDX46 | -3.780 | 0.000 | 2.589 | 0.000 |
| DOK3 | -1.591 | 0.000 | 1.005 | 0.035 |
| PHIP | -3.706 | 0.000 | 3.527 | 0.000 |
| TBC1D32 | -1.572 | 0.005 | 1.858 | 0.000 |
| ARHGAP18 | -2.467 | 0.000 | 1.403 | 0.000 |
| SHPRH | -1.848 | 0.000 | 1.891 | 0.000 |
| TIAM2 | -1.226 | 0.012 | 1.302 | 0.003 |
| ZMYM4 | -1.560 | 0.000 | 1.652 | 0.000 |
| VWDE | -1.061 | 0.037 | 1.136 | 0.004 |
| RBAK | -3.263 | 0.000 | 2.861 | 0.000 |
| CREB5 | 1.618 | 0.000 | 1.179 | 0.000 |
| LINC00525 | 2.324 | 0.003 | -1.530 | 0.012 |
| ZNF92 | -5.616 | 0.000 | 5.271 | 0.000 |
| ZNF182 | -3.051 | 0.000 | 2.795 | 0.000 |
| TAF1 | -1.441 | 0.000 | 1.457 | 0.000 |
| OGT | -2.092 | 0.000 | 2.387 | 0.000 |
| GCNA | -3.407 | 0.003 | 3.470 | 0.001 |
| DIAPH2 | -1.951 | 0.000 | 2.176 | 0.000 |
| DOCK11 | -2.150 | 0.000 | 1.732 | 0.000 |
| DOCK5 | -1.739 | 0.000 | 1.385 | 0.000 |
| NSD3 | -1.297 | 0.000 | 1.522 | 0.000 |
| TERF1 | -1.741 | 0.000 | 1.841 | 0.000 |
| UTP23 | -1.051 | 0.000 | 1.134 | 0.000 |
| FAM83A | 2.017 | 0.000 | -1.157 | 0.000 |
| NFIB | -1.641 | 0.000 | 1.537 | 0.000 |
| PLIN2 | 2.672 | 0.000 | -1.702 | 0.000 |
| HAUS6 | -1.127 | 0.000 | 1.285 | 0.000 |
| CEP78 | -1.218 | 0.000 | 1.484 | 0.000 |
| ZNF462 | -1.518 | 0.000 | 1.745 | 0.000 |
| UGCG | -1.309 | 0.000 | 1.266 | 0.000 |
| NR6A1 | -1.620 | 0.000 | 1.470 | 0.000 |
| LCN2 | 3.861 | 0.000 | -1.223 | 0.017 |
| ZEB1 | -2.957 | 0.000 | 3.321 | 0.000 |
| FAM13C | -2.424 | 0.000 | 1.320 | 0.012 |
| FRA10AC1 | -1.102 | 0.000 | 1.819 | 0.000 |
| ADD3 | -1.838 | 0.000 | 1.031 | 0.000 |
| MKI67 | -3.052 | 0.000 | 2.288 | 0.000 |
| FUOM | 1.308 | 0.000 | -1.226 | 0.000 |
| SLC43A1 | 3.588 | 0.000 | -1.111 | 0.000 |
| CCDC82 | -2.961 | 0.000 | 3.270 | 0.000 |
| NPAT | -2.879 | 0.000 | 2.691 | 0.000 |
| ATM | -2.855 | 0.000 | 2.586 | 0.000 |
| CCDC15 | -1.978 | 0.000 | 2.208 | 0.000 |
| FAU | 1.056 | 0.000 | -1.278 | 0.000 |
| ARID5B | -2.714 | 0.000 | 1.917 | 0.000 |
| FAM124A | -3.548 | 0.000 | 1.171 | 0.000 |
| MIA2 | -1.114 | 0.000 | 1.917 | 0.000 |
| PDCD4 | -1.302 | 0.000 | 1.091 | 0.000 |
| DOCK1 | -1.127 | 0.000 | 1.050 | 0.000 |
| NKAPD1 | -1.238 | 0.000 | 1.221 | 0.000 |
| FREM2 | -2.465 | 0.000 | 2.157 | 0.000 |
| CRIM1 | -1.534 | 0.000 | 1.311 | 0.000 |
| ITPR1 | -1.319 | 0.000 | 1.307 | 0.000 |
| ANK3 | -2.380 | 0.000 | 1.758 | 0.000 |
| CSNK1G3 | -1.060 | 0.000 | 1.224 | 0.000 |
| SRFBP1 | -2.088 | 0.000 | 2.144 | 0.000 |
| MIPOL1 | -2.831 | 0.000 | 3.186 | 0.000 |
| FER | -2.577 | 0.000 | 2.541 | 0.000 |
| UPF2 | -3.796 | 0.000 | 3.620 | 0.000 |
| SCLT1 | -1.287 | 0.000 | 1.161 | 0.000 |
| CCDC3 | 3.093 | 0.000 | -1.362 | 0.005 |
| FAM160B1 | -1.478 | 0.000 | 1.061 | 0.000 |
| TEX9 | -1.173 | 0.018 | 1.544 | 0.000 |
| WWC2 | -1.746 | 0.000 | 1.711 | 0.000 |
| CENPU | -1.401 | 0.000 | 1.590 | 0.000 |
| SACS | -2.967 | 0.000 | 2.835 | 0.000 |
| CENPJ | -4.679 | 0.000 | 4.558 | 0.000 |
| DST | -3.572 | 0.000 | 3.652 | 0.000 |
| BEND6 | -1.275 | 0.004 | 2.097 | 0.000 |
| SCHIP1 | -3.614 | 0.000 | 3.989 | 0.000 |
| MZT2B | 1.017 | 0.000 | -1.185 | 0.000 |
| GPATCH11 | -1.926 | 0.000 | 1.894 | 0.000 |
| CYSLTR2 | -3.429 | 0.000 | 1.813 | 0.013 |
| PDE3B | -2.080 | 0.000 | 1.495 | 0.000 |
| TGOLN2 | -1.023 | 0.000 | 1.309 | 0.000 |
| CWF19L2 | -3.826 | 0.000 | 3.367 | 0.000 |
| HOMER1 | -1.289 | 0.000 | 1.508 | 0.000 |
| DCLRE1C | -1.055 | 0.000 | 1.289 | 0.000 |
| PAN3 | -1.348 | 0.000 | 1.146 | 0.000 |
| IGSF10 | -5.095 | 0.000 | 2.840 | 0.000 |
| MBNL1 | -1.366 | 0.000 | 1.118 | 0.000 |
| UTRN | -2.949 | 0.000 | 2.830 | 0.000 |
| PLOD2 | -1.301 | 0.000 | 1.016 | 0.000 |
| SREK1IP1 | -3.296 | 0.000 | 2.990 | 0.000 |
| CWC27 | -2.861 | 0.000 | 3.134 | 0.000 |
| CAST | -1.995 | 0.000 | 1.690 | 0.000 |
| CLGN | 1.632 | 0.000 | 1.799 | 0.000 |
| SMARCA5 | -2.454 | 0.000 | 1.976 | 0.000 |
| RANBP2 | -4.072 | 0.000 | 3.633 | 0.000 |
| AHCTF1 | -4.066 | 0.000 | 4.011 | 0.000 |
| NR4A2 | -1.855 | 0.000 | 1.597 | 0.000 |
| TRAPPC8 | -1.332 | 0.000 | 1.051 | 0.000 |
| SREK1 | -4.467 | 0.000 | 4.227 | 0.000 |
| CHD1 | -3.555 | 0.000 | 3.896 | 0.000 |
| CACNA2D1 | -1.518 | 0.000 | 1.713 | 0.000 |
| SDHAF4 | 1.417 | 0.000 | -1.106 | 0.001 |
| ANGPT1 | -2.856 | 0.000 | 1.368 | 0.004 |
| CEP112 | -2.039 | 0.000 | 2.618 | 0.000 |
| ABCA5 | -1.416 | 0.000 | 1.594 | 0.000 |
| MIA3 | -2.306 | 0.000 | 2.510 | 0.000 |
| GABPA | -1.514 | 0.000 | 1.335 | 0.000 |
| ADAMTS5 | -1.133 | 0.000 | 1.015 | 0.000 |
| PIEZO2 | -2.970 | 0.000 | 2.078 | 0.000 |
| CCDC144B | -2.132 | 0.028 | 3.150 | 0.000 |
| OTUD6B | -1.109 | 0.000 | 1.398 | 0.000 |
| GOLGA7B | 3.784 | 0.004 | -2.159 | 0.030 |
| USP25 | -1.147 | 0.000 | 1.368 | 0.000 |
| TTN | -2.218 | 0.000 | 2.850 | 0.000 |
| RNF20 | -2.060 | 0.000 | 2.053 | 0.000 |
| PPARGC1B | -1.101 | 0.000 | 1.582 | 0.000 |
| RASA2 | -1.644 | 0.000 | 1.708 | 0.000 |
| PSD3 | -1.015 | 0.000 | 1.716 | 0.000 |
| CFAP70 | -3.562 | 0.002 | 4.083 | 0.000 |
| MMP16 | -1.904 | 0.000 | 2.219 | 0.000 |
| USP16 | -3.476 | 0.000 | 3.412 | 0.000 |
| RPGR | -1.790 | 0.000 | 2.189 | 0.000 |
| HKDC1 | 4.990 | 0.000 | -1.202 | 0.019 |
| KAT6B | -2.754 | 0.000 | 2.066 | 0.000 |
| MAPK13 | -2.431 | 0.002 | 2.587 | 0.000 |
| ATAD2 | -2.315 | 0.000 | 2.074 | 0.000 |
| SASS6 | -3.688 | 0.000 | 3.300 | 0.000 |
| BUB1B | -2.076 | 0.000 | 1.621 | 0.000 |
| EIF4A2 | -1.501 | 0.000 | 1.233 | 0.000 |
| SMG1 | -1.895 | 0.000 | 2.275 | 0.000 |
| FCHO2 | -2.167 | 0.000 | 1.917 | 0.000 |
| ODR4 | -1.788 | 0.000 | 1.936 | 0.000 |
| IL34 | 4.193 | 0.005 | -2.220 | 0.022 |
| APPL1 | -1.229 | 0.000 | 1.081 | 0.000 |
| DYRK1A | -1.052 | 0.000 | 1.074 | 0.000 |
| TAB3 | -1.024 | 0.000 | 1.443 | 0.000 |
| UBN2 | -1.440 | 0.000 | 1.476 | 0.000 |
| RNF207 | -2.117 | 0.000 | 1.070 | 0.000 |
| EIF5B | -4.453 | 0.000 | 4.044 | 0.000 |
| PPP1R9A | -2.139 | 0.000 | 2.638 | 0.000 |
| EMSY | -1.617 | 0.000 | 1.297 | 0.000 |
| ELK4 | -1.006 | 0.000 | 1.282 | 0.000 |
| DUSP23 | 1.758 | 0.000 | -1.191 | 0.000 |
| RAPGEF6 | -2.107 | 0.000 | 1.756 | 0.000 |
| PAXBP1 | -2.747 | 0.000 | 2.645 | 0.000 |
| SON | -1.772 | 0.000 | 1.358 | 0.000 |
| MORC3 | -1.475 | 0.000 | 1.444 | 0.000 |
| UBR1 | -1.206 | 0.000 | 1.165 | 0.000 |
| CCDC17 | -2.755 | 0.024 | 3.050 | 0.003 |
| TNFRSF13C | -1.161 | 0.024 | 2.158 | 0.000 |
| ABCG1 | 3.262 | 0.000 | -1.072 | 0.037 |
| ADAMTS13 | -1.254 | 0.029 | 1.591 | 0.000 |
| TAOK1 | -2.246 | 0.000 | 2.286 | 0.000 |
| PAQR6 | -1.307 | 0.022 | 1.704 | 0.000 |
| GPATCH4 | -2.146 | 0.000 | 2.320 | 0.000 |
| VPS28 | 1.016 | 0.000 | -1.003 | 0.000 |
| SQSTM1 | 1.667 | 0.000 | -1.350 | 0.000 |
| RPL8 | 1.015 | 0.000 | -1.221 | 0.000 |
| MFSD12 | 1.366 | 0.000 | -1.047 | 0.000 |
| MPP3 | -1.051 | 0.000 | 1.102 | 0.000 |
| JOSD2 | 1.298 | 0.000 | -1.436 | 0.000 |
| LARP4 | -1.412 | 0.000 | 1.291 | 0.000 |
| EIF4A1 | -1.590 | 0.000 | 2.395 | 0.000 |
| CCDC78 | -2.275 | 0.000 | 1.196 | 0.024 |
| MSLNL | -3.012 | 0.000 | 2.123 | 0.001 |
| AMDHD2 | 1.120 | 0.000 | -1.198 | 0.000 |
| ZNF75A | -2.398 | 0.000 | 2.254 | 0.000 |
| STX5 | 1.223 | 0.000 | -1.372 | 0.000 |
| USP24 | -1.768 | 0.000 | 1.325 | 0.000 |
| AKR7A3 | -2.460 | 0.011 | 2.304 | 0.006 |
| SCNN1D | -1.355 | 0.036 | 1.246 | 0.033 |
| NFIA | -1.690 | 0.000 | 1.523 | 0.000 |
| MYSM1 | -2.352 | 0.000 | 2.301 | 0.000 |
| USP1 | -1.630 | 0.000 | 1.466 | 0.000 |
| NEXN | -4.485 | 0.000 | 4.794 | 0.000 |
| TYW3 | -1.523 | 0.000 | 1.308 | 0.000 |
| FAM102B | -2.025 | 0.000 | 1.325 | 0.000 |
| ZNF326 | -2.796 | 0.000 | 2.116 | 0.000 |
| AGL | -1.712 | 0.000 | 1.487 | 0.000 |
| ZNF281 | -1.244 | 0.000 | 1.421 | 0.000 |
| BROX | -1.498 | 0.000 | 1.037 | 0.000 |
| NBPF20 | -1.606 | 0.000 | 1.316 | 0.000 |
| REL | -3.717 | 0.000 | 3.907 | 0.000 |
| PRORSD1P | -3.195 | 0.004 | 2.814 | 0.012 |
| CCDC138 | -1.269 | 0.000 | 1.230 | 0.000 |
| C2orf48 | -2.473 | 0.003 | 2.319 | 0.001 |
| ACTG2 | 1.028 | 0.000 | -1.393 | 0.000 |
| SMC6 | -4.471 | 0.000 | 4.523 | 0.000 |
| NOSTRIN | -1.271 | 0.046 | 1.544 | 0.003 |
| SGPP2 | -3.695 | 0.000 | 4.305 | 0.000 |
| SMARCAD1 | -1.969 | 0.000 | 1.823 | 0.000 |
| IWS1 | -2.234 | 0.000 | 2.205 | 0.000 |
| ARHGAP25 | 3.594 | 0.000 | -1.571 | 0.012 |
| ABRAXAS1 | -1.370 | 0.001 | 1.020 | 0.013 |
| LRRC58 | -1.600 | 0.000 | 1.269 | 0.000 |
| SSR2 | 1.104 | 0.000 | -1.302 | 0.000 |
| CIP2A | -3.567 | 0.000 | 2.933 | 0.000 |
| CWC22 | -4.591 | 0.000 | 4.616 | 0.000 |
| SGO2 | -4.667 | 0.000 | 4.012 | 0.000 |
| IFI16 | -2.105 | 0.000 | 1.681 | 0.000 |
| PPM1L | -1.363 | 0.000 | 1.447 | 0.000 |
| CCDC191 | -3.651 | 0.006 | 3.720 | 0.002 |
| ATXN7 | -2.803 | 0.005 | 2.110 | 0.045 |
| CCNL1 | -3.001 | 0.000 | 3.773 | 0.000 |
| SLMAP | -2.503 | 0.000 | 2.493 | 0.000 |
| RBM47 | -1.159 | 0.000 | 1.043 | 0.000 |
| U2SURP | -3.781 | 0.000 | 3.598 | 0.000 |
| TTC14 | -3.334 | 0.000 | 3.039 | 0.000 |
| TOPBP1 | -2.221 | 0.000 | 1.933 | 0.000 |
| SLC4A1AP | -1.921 | 0.000 | 1.598 | 0.000 |
| KIF15 | -4.758 | 0.000 | 4.655 | 0.000 |
| ZNF148 | -1.545 | 0.000 | 1.214 | 0.000 |
| RPL39L | 1.536 | 0.000 | -1.064 | 0.000 |
| GNL3 | -1.662 | 0.000 | 1.535 | 0.000 |
| PBRM1 | -1.534 | 0.000 | 1.194 | 0.000 |
| FAM208A | -2.060 | 0.000 | 2.116 | 0.000 |
| RNF168 | -1.488 | 0.000 | 1.400 | 0.000 |
| S100P | 1.871 | 0.000 | -1.125 | 0.000 |
| AIMP1 | -1.144 | 0.000 | 1.106 | 0.000 |
| DNAJB14 | -1.545 | 0.000 | 1.494 | 0.000 |
| HSPA4L | -1.910 | 0.000 | 2.706 | 0.000 |
| NAA15 | -2.886 | 0.000 | 2.516 | 0.000 |
| ICE1 | -2.729 | 0.000 | 2.440 | 0.000 |
| TMEM184C | -1.211 | 0.000 | 1.025 | 0.000 |
| NIPBL | -4.166 | 0.000 | 4.009 | 0.000 |
| CCDC112 | -5.198 | 0.000 | 5.563 | 0.000 |
| AGGF1 | -1.349 | 0.000 | 1.579 | 0.000 |
| CFAP97 | -1.346 | 0.000 | 1.541 | 0.000 |
| RICTOR | -1.941 | 0.000 | 1.848 | 0.000 |
| LEAP2 | -3.651 | 0.045 | 3.520 | 0.032 |
| CREBRF | -1.486 | 0.000 | 1.183 | 0.000 |
| TBX20 | -1.617 | 0.009 | 1.897 | 0.000 |
| SLU7 | -2.515 | 0.000 | 2.357 | 0.000 |
| USP49 | -1.344 | 0.000 | 1.151 | 0.000 |
| BRI3 | 1.045 | 0.000 | -1.348 | 0.000 |
| DEFB1 | 1.242 | 0.002 | -2.031 | 0.000 |
| OXR1 | -1.424 | 0.000 | 1.439 | 0.000 |
| FMC1 | 1.141 | 0.015 | -1.055 | 0.012 |
| PSIP1 | -3.008 | 0.000 | 3.107 | 0.000 |
| CCDC171 | -1.886 | 0.000 | 2.306 | 0.000 |
| SVEP1 | -1.206 | 0.000 | 1.021 | 0.000 |
| ZHX1 | -2.466 | 0.000 | 2.218 | 0.000 |
| BRWD3 | -2.416 | 0.000 | 2.128 | 0.000 |
| ARHGAP12 | -1.366 | 0.000 | 1.387 | 0.000 |
| HECTD2 | -1.122 | 0.000 | 1.175 | 0.000 |
| PGM2L1 | -2.647 | 0.000 | 1.620 | 0.000 |
| MICU2 | -1.439 | 0.000 | 1.302 | 0.000 |
| DDIAS | -1.129 | 0.000 | 1.169 | 0.000 |
| PCF11 | -2.629 | 0.000 | 2.570 | 0.000 |
| NEMF | -4.814 | 0.000 | 4.574 | 0.000 |
| TAF3 | -1.213 | 0.000 | 1.454 | 0.000 |
| PDZD8 | -2.124 | 0.000 | 2.028 | 0.000 |
| FAM204A | -1.133 | 0.000 | 1.135 | 0.000 |
| NSD1 | -1.136 | 0.000 | 1.186 | 0.000 |
| DDX21 | -2.137 | 0.000 | 2.275 | 0.000 |
| BMS1 | -2.116 | 0.000 | 1.998 | 0.000 |
| CCDC186 | -3.221 | 0.000 | 2.964 | 0.000 |
| CACNB2 | -1.598 | 0.000 | 1.123 | 0.011 |
| CEP295 | -4.028 | 0.000 | 4.079 | 0.000 |
| TAF1D | -1.606 | 0.000 | 1.585 | 0.000 |
| CEP57 | -1.414 | 0.000 | 1.346 | 0.000 |
| IKBIP | -1.412 | 0.000 | 1.090 | 0.000 |
| FBN1 | -1.175 | 0.000 | 1.308 | 0.000 |
| BRD7 | -1.344 | 0.000 | 1.286 | 0.000 |
| NOLC1 | -1.393 | 0.000 | 1.128 | 0.000 |
| COPS2 | -1.822 | 0.000 | 1.289 | 0.000 |
| STXBP4 | -1.520 | 0.000 | 1.225 | 0.000 |
| CUL5 | -1.724 | 0.000 | 1.585 | 0.000 |
| PLEKHF1 | 1.092 | 0.000 | -1.506 | 0.000 |
| TRIM66 | -1.257 | 0.000 | 1.813 | 0.000 |
| RNF169 | -1.584 | 0.000 | 1.609 | 0.000 |
| LEO1 | -3.217 | 0.000 | 3.025 | 0.000 |
| CCDC68 | -1.670 | 0.000 | 1.871 | 0.000 |
| RIMKLB | -1.332 | 0.000 | 1.150 | 0.000 |
| HSP90B1 | 4.358 | 0.000 | -4.402 | 0.000 |
| CASC4 | -1.145 | 0.000 | 1.410 | 0.000 |
| CATSPER2 | -2.225 | 0.003 | 2.505 | 0.000 |
| PPIB | 1.040 | 0.000 | -1.226 | 0.000 |
| FAM111A | -1.087 | 0.000 | 1.067 | 0.000 |
| ANKDD1A | -1.905 | 0.008 | 2.083 | 0.000 |
| C18orf54 | -1.016 | 0.000 | 1.354 | 0.000 |
| C15orf48 | 1.113 | 0.011 | -1.577 | 0.000 |
| MAP1A | -1.311 | 0.000 | 1.190 | 0.000 |
| FAM102A | 1.929 | 0.000 | -1.842 | 0.000 |
| ZNF91 | -2.349 | 0.000 | 3.061 | 0.000 |
| ZNF180 | -1.606 | 0.000 | 1.474 | 0.000 |
| MVD | 1.652 | 0.000 | -1.936 | 0.000 |
| SGK494 | -1.690 | 0.022 | 1.626 | 0.021 |
| ZNF701 | -1.253 | 0.000 | 1.200 | 0.000 |
| ZNF146 | -1.884 | 0.000 | 2.226 | 0.000 |
| YIF1B | 1.149 | 0.000 | -1.189 | 0.000 |
| RILP | 1.053 | 0.000 | -1.598 | 0.000 |
| ZNF83 | -2.551 | 0.000 | 2.854 | 0.000 |
| ANGPTL4 | 4.125 | 0.000 | -1.021 | 0.000 |
| CD320 | 1.035 | 0.000 | -1.163 | 0.000 |
| MLST8 | 1.185 | 0.000 | -1.195 | 0.000 |
| ECI1 | 1.094 | 0.000 | -1.008 | 0.000 |
| RAB3IL1 | 2.407 | 0.000 | -1.782 | 0.000 |
| FTH1 | 1.337 | 0.000 | -1.427 | 0.000 |
| KIAA1586 | -2.332 | 0.000 | 1.731 | 0.000 |
| THAP9 | -1.942 | 0.000 | 1.722 | 0.000 |
| RNF187 | 1.648 | 0.000 | -1.288 | 0.000 |
| HOOK3 | -2.652 | 0.000 | 2.300 | 0.000 |
| PCMTD1 | -1.424 | 0.000 | 1.097 | 0.000 |
| CAVIN2 | -1.858 | 0.000 | 2.045 | 0.000 |
| ADAM9 | -1.116 | 0.000 | 1.096 | 0.000 |
| LRP1B | -1.268 | 0.000 | 1.678 | 0.000 |
| DNAJC21 | -2.492 | 0.000 | 2.514 | 0.000 |
| TET2 | -2.158 | 0.000 | 2.225 | 0.000 |
| ZNF507 | -1.164 | 0.000 | 1.161 | 0.000 |
| ANKRD49 | -1.379 | 0.000 | 1.347 | 0.000 |
| RNF181 | 1.376 | 0.000 | -1.356 | 0.000 |
| CEP120 | -1.235 | 0.000 | 2.078 | 0.000 |
| JMJD7-PLA2G4B | -2.081 | 0.046 | 2.318 | 0.006 |
| COL4A3 | -1.664 | 0.000 | 1.786 | 0.000 |
| HNRNPH1 | -1.404 | 0.000 | 1.317 | 0.000 |
| UPF3A | -1.315 | 0.000 | 1.401 | 0.000 |
| ZNF354A | -1.778 | 0.000 | 1.817 | 0.000 |
| CPT1C | 1.357 | 0.010 | -1.255 | 0.005 |
| NPIPB12 | -2.851 | 0.002 | 2.508 | 0.005 |
| RGS14 | 1.629 | 0.000 | -1.664 | 0.000 |
| NPIPB3 | -2.537 | 0.000 | 1.975 | 0.001 |
| ZRSR2 | -1.568 | 0.000 | 1.724 | 0.000 |
| KCNAB1 | -2.582 | 0.000 | 2.671 | 0.000 |
| NR0B1 | 2.526 | 0.000 | -1.394 | 0.004 |
| ARL13B | -1.245 | 0.000 | 1.489 | 0.000 |
| CKAP2L | -3.641 | 0.000 | 3.186 | 0.000 |
| BOLA2B | 4.648 | 0.000 | -4.089 | 0.001 |
| RGPD8 | -1.266 | 0.001 | 1.910 | 0.000 |
| LUZP1 | -1.715 | 0.000 | 1.388 | 0.000 |
| RAC3 | 1.058 | 0.000 | -1.216 | 0.000 |
| PCDH7 | -1.629 | 0.000 | 1.526 | 0.000 |
| ROBO1 | -2.115 | 0.000 | 2.079 | 0.000 |
| TOR1AIP2 | -1.576 | 0.000 | 1.229 | 0.000 |
| OTUD3 | -1.559 | 0.000 | 1.603 | 0.000 |
| MAP3K2 | -1.462 | 0.000 | 1.595 | 0.000 |
| ALCAM | -2.654 | 0.000 | 1.992 | 0.000 |
| ADPRM | 1.454 | 0.000 | -1.041 | 0.000 |
| USP47 | -2.150 | 0.000 | 2.612 | 0.000 |
| FAM161A | -2.160 | 0.000 | 2.532 | 0.000 |
| PRDM10 | -1.561 | 0.000 | 1.013 | 0.000 |
| FOS | -1.933 | 0.000 | 1.038 | 0.000 |
| ZNF804A | -3.267 | 0.022 | 2.841 | 0.048 |
| KIF5B | -3.610 | 0.000 | 3.460 | 0.000 |
| ZNF160 | -1.963 | 0.000 | 2.176 | 0.000 |
| CAVIN3 | 1.018 | 0.000 | -1.102 | 0.000 |
| NRTN | 3.398 | 0.000 | -1.188 | 0.000 |
| KCNMB3 | -2.823 | 0.018 | 3.202 | 0.001 |
| C9orf16 | 1.234 | 0.000 | -1.165 | 0.000 |
| ZNF440 | -1.874 | 0.000 | 2.215 | 0.000 |
| KCNK3 | 1.100 | 0.000 | -1.369 | 0.000 |
| CHD7 | -1.637 | 0.000 | 2.256 | 0.000 |
| PDE7B | -1.659 | 0.000 | 1.226 | 0.000 |
| ZNF524 | 1.411 | 0.000 | -1.227 | 0.000 |
| DLK2 | 1.270 | 0.000 | -1.002 | 0.001 |
| ZNF318 | -1.100 | 0.000 | 1.010 | 0.000 |
| RSL1D1 | -1.988 | 0.000 | 1.810 | 0.000 |
| SPSB1 | 1.156 | 0.000 | -1.153 | 0.000 |
| BPTF | -3.137 | 0.000 | 2.815 | 0.000 |
| ATF7IP | -1.796 | 0.000 | 1.539 | 0.000 |
| SLFNL1 | -3.460 | 0.000 | 3.526 | 0.000 |
| PWWP2B | 1.377 | 0.000 | -1.029 | 0.000 |
| EXOSC10 | -1.410 | 0.000 | 1.049 | 0.000 |
| MLLT3 | -2.354 | 0.000 | 2.097 | 0.000 |
| SRGAP2C | -1.344 | 0.000 | 1.240 | 0.000 |
| SHLD1 | 1.590 | 0.001 | -1.614 | 0.000 |
| JMJD1C | -3.368 | 0.000 | 3.611 | 0.000 |
| MALT1 | -1.702 | 0.000 | 1.148 | 0.000 |
| ISG20 | 1.881 | 0.000 | -1.690 | 0.000 |
| NEGR1 | -1.257 | 0.000 | 1.443 | 0.000 |
| MANEA | -1.687 | 0.000 | 1.482 | 0.000 |
| PDE3A | -2.049 | 0.000 | 1.064 | 0.000 |
| RASGRP1 | -1.882 | 0.026 | 2.042 | 0.002 |
| NAA16 | -2.647 | 0.000 | 2.337 | 0.000 |
| DCP2 | -1.091 | 0.000 | 1.105 | 0.000 |
| DHCR7 | 1.079 | 0.000 | -1.759 | 0.000 |
| ZNF680 | -1.319 | 0.000 | 1.933 | 0.000 |
| HECTD4 | -1.259 | 0.000 | 1.038 | 0.000 |
| NOC3L | -1.780 | 0.000 | 1.893 | 0.000 |
| PARP14 | -1.079 | 0.000 | 1.456 | 0.000 |
| AHSA2P | -2.875 | 0.000 | 2.701 | 0.000 |
| GOLGB1 | -3.743 | 0.000 | 3.385 | 0.000 |
| C11orf86 | -1.660 | 0.000 | -1.493 | 0.000 |
| MZT2A | 1.002 | 0.000 | -1.159 | 0.000 |
| STOX2 | -2.004 | 0.000 | 1.422 | 0.000 |
| SAA1 | 1.578 | 0.030 | -1.669 | 0.007 |
| PPP1R14B | 1.031 | 0.000 | -1.182 | 0.000 |
| SMARCC1 | -1.260 | 0.000 | 1.110 | 0.000 |
| PEAK1 | -1.312 | 0.000 | 1.425 | 0.000 |
| MST1 | -1.446 | 0.010 | 1.162 | 0.022 |
| MOB1B | -1.728 | 0.000 | 2.178 | 0.000 |
| CHD2 | -1.877 | 0.000 | 2.234 | 0.000 |
| CEP83 | -4.015 | 0.000 | 4.143 | 0.000 |
| BCLAF3 | -1.496 | 0.001 | 2.213 | 0.000 |
| ZNF791 | -1.100 | 0.000 | 1.330 | 0.000 |
| PHC3 | -1.755 | 0.000 | 1.458 | 0.000 |
| GOLIM4 | -4.343 | 0.000 | 3.533 | 0.000 |
| RBM4 | -1.434 | 0.000 | 1.020 | 0.000 |
| TRMT10C | -1.759 | 0.000 | 1.422 | 0.000 |
| MGA | -1.957 | 0.000 | 1.776 | 0.000 |
| PHLDA3 | 1.461 | 0.000 | -1.036 | 0.000 |
| RALGAPA1 | -1.682 | 0.000 | 2.213 | 0.000 |
| LIG4 | -1.423 | 0.000 | 1.434 | 0.000 |
| DENND4A | -1.319 | 0.000 | 1.587 | 0.000 |
| ANKRD36C | -2.015 | 0.001 | 1.690 | 0.002 |
| GOLT1A | 3.706 | 0.000 | -1.608 | 0.003 |
| MSL2 | -1.401 | 0.000 | 1.216 | 0.000 |
| KIAA1551 | -3.739 | 0.000 | 3.690 | 0.000 |
| LARP7 | -5.639 | 0.000 | 5.099 | 0.000 |
| CEP135 | -4.405 | 0.000 | 4.342 | 0.000 |
| RSRC1 | -2.305 | 0.000 | 2.173 | 0.000 |
| DHX36 | -1.597 | 0.000 | 1.727 | 0.000 |
| ATR | -1.939 | 0.000 | 1.535 | 0.000 |
| ZNF654 | -1.983 | 0.000 | 1.341 | 0.000 |
| CKAP5 | -1.717 | 0.000 | 1.620 | 0.000 |
| GOLGA8A | -3.708 | 0.000 | 3.600 | 0.000 |
| CCDC14 | -2.381 | 0.000 | 2.244 | 0.000 |
| TOMM5 | -2.656 | 0.000 | 2.524 | 0.000 |
| SFN | 1.037 | 0.000 | -1.150 | 0.000 |
| YES1 | -1.190 | 0.000 | 1.116 | 0.000 |
| ATAD5 | -4.203 | 0.000 | 3.848 | 0.000 |
| COX8A | 1.070 | 0.000 | -1.173 | 0.000 |
| VPS37D | 5.600 | 0.000 | -1.340 | 0.009 |
| WDR25 | 1.043 | 0.000 | -1.036 | 0.000 |
| PRR15 | 1.015 | 0.000 | -1.040 | 0.000 |
| USF3 | -1.915 | 0.000 | 1.551 | 0.000 |
| CCDC121 | -1.205 | 0.029 | 1.534 | 0.001 |
| NCKAP5 | -1.576 | 0.007 | 1.671 | 0.001 |
| TCIM | -1.666 | 0.000 | 1.820 | 0.000 |
| FIBIN | 5.736 | 0.000 | -1.849 | 0.001 |
| MTX3 | -1.069 | 0.000 | 1.818 | 0.000 |
| CHD9 | -2.608 | 0.000 | 2.669 | 0.000 |
| ZBTB38 | -1.485 | 0.000 | 1.642 | 0.000 |
| RBM44 | -2.672 | 0.012 | 2.986 | 0.000 |
| THAP5 | -1.164 | 0.000 | 1.309 | 0.000 |
| ZNF518A | -2.737 | 0.000 | 3.258 | 0.000 |
| MAMDC4 | -1.225 | 0.035 | 1.876 | 0.000 |
| ODF3B | 1.573 | 0.000 | -1.172 | 0.001 |
| C2orf69 | -1.083 | 0.000 | 1.203 | 0.000 |
| PDE4DIP | -1.545 | 0.000 | 1.039 | 0.000 |
| DDX10 | -2.715 | 0.000 | 2.605 | 0.000 |
| NDUFV2 | -2.699 | 0.000 | 2.366 | 0.000 |
| LCORL | -2.450 | 0.000 | 2.051 | 0.000 |
| PRSS36 | 2.065 | 0.001 | -1.153 | 0.030 |
| GEN1 | -2.866 | 0.000 | 2.444 | 0.000 |
| TMPRSS9 | -1.531 | 0.006 | 1.496 | 0.000 |
| ZNF354B | -1.281 | 0.024 | 1.822 | 0.000 |
| KLHL11 | -1.208 | 0.000 | 1.348 | 0.000 |
| EPM2AIP1 | -1.850 | 0.000 | 1.758 | 0.000 |
| SUZ12 | -2.214 | 0.000 | 1.992 | 0.000 |
| TMEM52 | 2.100 | 0.000 | -1.659 | 0.001 |
| MSC | 3.624 | 0.000 | -1.375 | 0.000 |
| SPTY2D1 | -1.277 | 0.000 | 1.193 | 0.000 |
| PTPN11 | -1.128 | 0.000 | 1.158 | 0.000 |
| LINC00174 | -1.496 | 0.000 | 1.965 | 0.000 |
| LACC1 | -1.408 | 0.000 | 2.124 | 0.000 |
| ARL14 | -2.198 | 0.031 | 2.299 | 0.012 |
| PPP1R14BP3 | 1.012 | 0.000 | -1.251 | 0.000 |
| SOCS4 | -1.177 | 0.000 | 1.495 | 0.000 |
| ANKRD18A | -3.342 | 0.000 | 3.706 | 0.000 |
| HERC2P3 | -2.062 | 0.000 | 2.086 | 0.000 |
| FGD6 | -1.498 | 0.000 | 1.539 | 0.000 |
| MEIOC | -2.296 | 0.000 | 2.155 | 0.000 |
| CCDC66 | -2.757 | 0.000 | 2.860 | 0.000 |
| NRIP1 | -2.776 | 0.000 | 2.217 | 0.000 |
| SKIDA1 | -2.455 | 0.000 | 1.594 | 0.000 |
| HIST1H2BC | -1.379 | 0.000 | 1.047 | 0.000 |
| YOD1 | -2.202 | 0.000 | 1.960 | 0.000 |
| SMG1P3 | -1.509 | 0.002 | 1.407 | 0.002 |
| GREM2 | -2.700 | 0.000 | 1.265 | 0.001 |
| SSR4 | 1.126 | 0.000 | -1.208 | 0.000 |
| OXTR | -4.628 | 0.000 | 4.449 | 0.000 |
| TRAPPC5 | 2.114 | 0.008 | -1.256 | 0.048 |
| DDX60L | -1.738 | 0.000 | 1.727 | 0.000 |
| ZNF678 | -2.105 | 0.000 | 2.283 | 0.000 |
| FANCB | -1.168 | 0.000 | 1.151 | 0.000 |
| SETD2 | -2.894 | 0.000 | 2.501 | 0.000 |
| C6orf223 | 2.606 | 0.000 | -2.748 | 0.000 |
| GPR135 | -1.929 | 0.012 | 2.199 | 0.000 |
| PLAG1 | -1.672 | 0.000 | 1.634 | 0.000 |
| ZBTB20 | -2.895 | 0.000 | 2.334 | 0.000 |
| RFX7 | -1.319 | 0.000 | 1.026 | 0.000 |
| ZNF708 | -2.412 | 0.000 | 2.614 | 0.000 |
| ERCC6L2 | -2.271 | 0.000 | 2.403 | 0.000 |
| SHMT2 | 1.600 | 0.000 | -1.014 | 0.000 |
| FIGN | -1.362 | 0.000 | 1.751 | 0.000 |
| TSHZ2 | -2.226 | 0.000 | 1.520 | 0.000 |
| CEP97 | -1.201 | 0.000 | 1.215 | 0.000 |
| PLCB1 | -1.289 | 0.000 | 1.212 | 0.000 |
| TTC3 | -3.789 | 0.000 | 3.647 | 0.000 |
| PAPPA | -1.137 | 0.000 | 1.239 | 0.000 |
| ZNF721 | -3.261 | 0.000 | 3.508 | 0.000 |
| CEP63 | -2.619 | 0.000 | 2.733 | 0.000 |
| SPATA13 | -1.412 | 0.000 | 1.251 | 0.000 |
| CEP57L1 | -2.240 | 0.000 | 2.242 | 0.000 |
| C2CD4C | 2.592 | 0.003 | -1.539 | 0.026 |
| RPSAP19 | 1.006 | 0.000 | -1.389 | 0.000 |
| KIAA2026 | -3.380 | 0.000 | 3.501 | 0.000 |
| FHL3 | 1.266 | 0.000 | -1.056 | 0.000 |
| PRR14L | -2.049 | 0.000 | 2.081 | 0.000 |
| GPR1 | 4.273 | 0.000 | -1.471 | 0.000 |
| NOG | -1.403 | 0.007 | 1.443 | 0.002 |
| UPP1 | 2.496 | 0.000 | -1.299 | 0.000 |
| TRIM52 | -2.169 | 0.000 | 1.971 | 0.000 |
| GPR173 | -1.024 | 0.013 | 1.027 | 0.005 |
| C22orf46 | -1.382 | 0.000 | 2.377 | 0.000 |
| ACOT1 | 1.360 | 0.000 | -1.247 | 0.000 |
| MAML2 | -2.332 | 0.000 | 2.077 | 0.000 |
| KNTC1 | -1.558 | 0.000 | 1.438 | 0.000 |
| WDR27 | -1.557 | 0.000 | 1.800 | 0.000 |
| CDCA2 | -2.137 | 0.000 | 1.123 | 0.000 |
| SMIM10 | -1.975 | 0.000 | 1.599 | 0.000 |
| FAM227A | -1.858 | 0.004 | 2.347 | 0.000 |
| TMEM121 | 2.096 | 0.000 | -1.048 | 0.001 |
| ZNF445 | -1.104 | 0.000 | 1.284 | 0.000 |
| TCEAL9 | -1.725 | 0.000 | 1.074 | 0.000 |
| PRPF39 | -1.943 | 0.000 | 2.371 | 0.000 |
| KIAA0825 | -2.304 | 0.000 | 1.039 | 0.000 |
| ZBTB37 | -1.691 | 0.000 | 1.869 | 0.000 |
| L3MBTL1 | -1.447 | 0.001 | 1.278 | 0.001 |
| BRCC3 | -2.916 | 0.000 | 2.726 | 0.000 |
| BRWD1 | -1.415 | 0.000 | 1.460 | 0.000 |
| MYBL1 | -1.617 | 0.000 | 1.533 | 0.000 |
| SMG1P4 | -2.487 | 0.040 | 2.449 | 0.029 |
| KCNIP4 | -3.000 | 0.001 | 2.775 | 0.002 |
| NPIPB4 | -2.499 | 0.000 | 1.802 | 0.010 |
| PTCH1 | -1.525 | 0.000 | 1.490 | 0.000 |
| RNPC3 | -1.630 | 0.000 | 1.450 | 0.000 |
| ZNF267 | -2.779 | 0.000 | 2.957 | 0.000 |
| SDHAP3 | -3.363 | 0.000 | 3.068 | 0.000 |
| MRTFB | -1.125 | 0.000 | 1.148 | 0.000 |
| ZNF197 | -1.206 | 0.000 | 1.790 | 0.000 |
| PCLO | 1.410 | 0.003 | 1.573 | 0.000 |
| INSIG1 | 1.367 | 0.000 | -2.677 | 0.000 |
| GPATCH8 | -1.373 | 0.000 | 1.204 | 0.000 |
| SMIM29 | 1.227 | 0.000 | -1.082 | 0.000 |
| LYRM7 | -1.331 | 0.000 | 1.227 | 0.000 |
| KRT16 | 4.380 | 0.000 | -2.251 | 0.000 |
| TRABD2A | -1.721 | 0.000 | 1.090 | 0.000 |
| RTN4RL2 | 1.055 | 0.000 | -1.017 | 0.000 |
| ZDHHC17 | -1.233 | 0.000 | 1.005 | 0.000 |
| RHD | -1.144 | 0.016 | 1.199 | 0.005 |
| TEAD1 | -2.209 | 0.000 | 2.270 | 0.000 |
| PLCD1 | 1.244 | 0.043 | -1.233 | 0.034 |
| MITF | -1.741 | 0.000 | 1.250 | 0.000 |
| AKR1C1 | 1.548 | 0.000 | -1.738 | 0.000 |
| SHTN1 | -2.057 | 0.000 | 1.998 | 0.000 |
| DYNC2H1 | -3.242 | 0.000 | 3.228 | 0.000 |
| RSBN1L | -1.936 | 0.000 | 1.252 | 0.000 |
| PLEKHN1 | -1.241 | 0.041 | 1.098 | 0.046 |
| ZNF286A | -1.290 | 0.000 | 1.321 | 0.000 |
| FANCM | -3.147 | 0.000 | 2.961 | 0.000 |
| EIF4EBP1 | 1.996 | 0.000 | -1.016 | 0.000 |
| AC091057.1 | -2.625 | 0.000 | 2.108 | 0.000 |
| EYS | -2.154 | 0.003 | 2.174 | 0.001 |
| COL4A5 | -1.395 | 0.000 | 1.047 | 0.000 |
| NHS | -2.192 | 0.000 | 2.103 | 0.000 |
| TMPPE | -1.372 | 0.000 | 1.268 | 0.000 |
| LAMTOR4 | 1.030 | 0.000 | -1.150 | 0.000 |
| AGAP4 | -1.855 | 0.000 | 1.269 | 0.003 |
| C3orf62 | -1.780 | 0.000 | 1.462 | 0.000 |
| SRSF10 | -1.375 | 0.000 | 1.022 | 0.000 |
| DUSP28 | -1.314 | 0.003 | 1.019 | 0.013 |
| TMEM198 | 1.428 | 0.000 | -1.047 | 0.000 |
| ZDHHC11 | -2.405 | 0.000 | 2.202 | 0.000 |
| ZNF292 | -4.241 | 0.000 | 3.536 | 0.000 |
| ZNF567 | -1.010 | 0.026 | 1.006 | 0.016 |
| FAM111B | -4.787 | 0.000 | 4.548 | 0.000 |
| ARID2 | -1.936 | 0.000 | 2.123 | 0.000 |
| ZNF33A | -2.499 | 0.000 | 1.898 | 0.000 |
| ZNF600 | -1.463 | 0.007 | 1.867 | 0.000 |
| BTBD8 | -2.875 | 0.038 | 3.369 | 0.003 |
| LIN54 | -1.132 | 0.000 | 1.174 | 0.000 |
| RPS2P46 | 1.000 | 0.000 | -1.263 | 0.000 |
| SH2D5 | 2.917 | 0.000 | -1.241 | 0.000 |
| USP32P3 | -4.526 | 0.000 | 4.924 | 0.000 |
| ZNF724 | -2.385 | 0.001 | 2.625 | 0.000 |
| FAT4 | -2.842 | 0.000 | 2.231 | 0.000 |
| ACADSB | -1.108 | 0.000 | 1.340 | 0.000 |
| MPHOSPH8 | -3.085 | 0.000 | 2.813 | 0.000 |
| FAM217B | -1.204 | 0.000 | 1.307 | 0.000 |
| LCOR | -1.298 | 0.000 | 1.453 | 0.000 |
| ZNF107 | -3.061 | 0.000 | 3.172 | 0.000 |
| ZNF493 | -2.381 | 0.020 | 2.940 | 0.000 |
| ATP2A1 | -1.862 | 0.000 | 1.458 | 0.007 |
| ZNF765 | -2.576 | 0.000 | 2.371 | 0.000 |
| ZNF605 | -2.289 | 0.000 | 2.575 | 0.000 |
| PRPF40A | -3.848 | 0.000 | 3.510 | 0.000 |
| CACNA1H | 1.559 | 0.012 | -1.035 | 0.046 |
| XRCC2 | -1.362 | 0.000 | 1.883 | 0.000 |
| MYO6 | -2.473 | 0.000 | 2.581 | 0.000 |
| ZNF782 | -2.038 | 0.000 | 2.006 | 0.000 |
| TCF4 | -2.382 | 0.000 | 1.719 | 0.000 |
| LINC00173 | -3.008 | 0.000 | 2.558 | 0.000 |
| TRPV1 | -2.705 | 0.001 | 3.199 | 0.000 |
| ZNF33B | -1.782 | 0.000 | 1.587 | 0.000 |
| PDXDC2P-NPIPB14P | -2.903 | 0.001 | 2.740 | 0.001 |
| ZNF431 | -1.533 | 0.000 | 1.782 | 0.000 |
| NF1 | -1.447 | 0.000 | 1.211 | 0.000 |
| CLPSL2 | 2.674 | 0.004 | -1.924 | 0.011 |
| ZNF700 | -1.764 | 0.000 | 2.206 | 0.000 |
| MAML3 | -1.465 | 0.000 | 1.164 | 0.000 |
| STRN3 | -1.376 | 0.000 | 1.135 | 0.000 |
| ANKRD36B | -1.973 | 0.000 | 2.020 | 0.000 |
| ARHGEF12 | -1.561 | 0.000 | 1.279 | 0.000 |
| ZNF252P | -1.007 | 0.000 | 1.675 | 0.000 |
| ZNF100 | -2.657 | 0.000 | 3.386 | 0.000 |
| ZNF441 | -4.512 | 0.000 | 5.312 | 0.000 |
| ZMYM1 | -1.271 | 0.000 | 1.203 | 0.000 |
| PGAP1 | -1.964 | 0.000 | 2.009 | 0.000 |
| CH17-340M24.3 | 2.206 | 0.001 | -1.626 | 0.003 |
| BLM | -1.540 | 0.000 | 1.236 | 0.000 |
| ZNF720 | -1.116 | 0.001 | 1.159 | 0.000 |
| DDI2 | -1.253 | 0.000 | 1.195 | 0.000 |
| TRIM33 | -2.024 | 0.000 | 1.898 | 0.000 |
| ZNF675 | -3.057 | 0.000 | 2.821 | 0.000 |
| ZNF860 | -2.121 | 0.000 | 1.563 | 0.004 |
| ZNF695 | -1.542 | 0.003 | 1.492 | 0.001 |
| MYO5A | -1.846 | 0.000 | 2.403 | 0.000 |
| TOPORS | -3.689 | 0.000 | 3.483 | 0.000 |
| GPX1P1 | 1.140 | 0.000 | -1.307 | 0.000 |
| CPLANE1 | -3.359 | 0.000 | 3.081 | 0.000 |
| ZNF841 | -1.376 | 0.003 | 1.901 | 0.000 |
| ZNF615 | -1.404 | 0.027 | 1.796 | 0.001 |
| CFD | 1.179 | 0.000 | -1.178 | 0.000 |
| EME2 | -1.390 | 0.000 | 1.017 | 0.000 |
| ZNF461 | -4.472 | 0.019 | 3.943 | 0.043 |
| ZNF181 | -1.181 | 0.000 | 1.263 | 0.000 |
| ZNF121 | -2.740 | 0.000 | 2.810 | 0.000 |
| VPS13A | -3.789 | 0.000 | 3.492 | 0.000 |
| GOLGA6L9 | -1.712 | 0.003 | 2.754 | 0.000 |
| NOL8 | -3.909 | 0.000 | 3.738 | 0.000 |
| ZNF84 | -1.893 | 0.000 | 2.540 | 0.000 |
| CD2AP | -2.546 | 0.000 | 2.463 | 0.000 |
| MB | 1.256 | 0.003 | -1.617 | 0.000 |
| ZNF770 | -1.024 | 0.000 | 1.096 | 0.000 |
| HMGN5 | -5.247 | 0.000 | 4.956 | 0.000 |
| MAN1A2 | -1.356 | 0.000 | 1.307 | 0.000 |
| HELZ | -2.131 | 0.000 | 1.669 | 0.000 |
| SDAD1 | -2.453 | 0.000 | 2.395 | 0.000 |
| ZNF813 | -1.769 | 0.000 | 1.840 | 0.000 |
| ITSN2 | -2.971 | 0.000 | 3.270 | 0.000 |
| OGA | -1.147 | 0.000 | 1.049 | 0.000 |
| TCAF1 | -2.300 | 0.000 | 2.838 | 0.000 |
| ZNF480 | -2.815 | 0.000 | 2.905 | 0.000 |
| ZNF808 | -1.968 | 0.000 | 1.819 | 0.000 |
| ZNF28 | -1.313 | 0.000 | 1.902 | 0.000 |
| WDHD1 | -2.060 | 0.000 | 2.147 | 0.000 |
| DDX39B | -2.421 | 0.000 | 2.169 | 0.000 |
| TLK1 | -2.275 | 0.000 | 2.017 | 0.000 |
| BAZ1A | -4.152 | 0.000 | 3.973 | 0.000 |
| TTC37 | -2.328 | 0.000 | 2.267 | 0.000 |
| CEP290 | -3.464 | 0.000 | 3.817 | 0.000 |
| TOGARAM1 | -1.384 | 0.000 | 1.234 | 0.000 |
| CTR9 | -2.420 | 0.000 | 2.327 | 0.000 |
| ZNF652 | -1.550 | 0.000 | 1.064 | 0.000 |
| SLC5A3 | -1.143 | 0.000 | 1.495 | 0.000 |
| RASSF9 | -2.384 | 0.000 | 1.598 | 0.001 |
| ZNF358 | 1.148 | 0.000 | -1.125 | 0.000 |
| ARHGAP11A | -2.582 | 0.000 | 2.286 | 0.000 |
| GJC2 | 1.887 | 0.000 | -1.152 | 0.000 |
| OPA1 | -1.862 | 0.000 | 1.578 | 0.000 |
| RYR3 | -3.089 | 0.000 | 2.602 | 0.000 |
| MT-ND3 | 1.161 | 0.000 | -1.044 | 0.000 |
| FICD | 2.172 | 0.000 | -1.555 | 0.000 |
| LTN1 | -1.591 | 0.000 | 1.476 | 0.000 |
| SMC5 | -4.232 | 0.000 | 3.773 | 0.000 |
| SHISA4 | 1.483 | 0.000 | -1.501 | 0.000 |
| TOP1 | -2.298 | 0.000 | 1.984 | 0.000 |
| DZIP3 | -2.329 | 0.000 | 2.460 | 0.000 |
| SNORA73B | -2.703 | 0.002 | 2.118 | 0.019 |
| SNORA33 | -3.118 | 0.001 | 2.727 | 0.004 |
| SNORA55 | -3.528 | 0.003 | 3.464 | 0.002 |
| SNORA71C | -2.059 | 0.003 | 1.902 | 0.004 |
| ZNF525 | -1.875 | 0.000 | 2.183 | 0.000 |
| CHML | -3.287 | 0.000 | 2.966 | 0.000 |
| SAMD5 | -1.375 | 0.000 | 1.173 | 0.000 |
| HIST2H2BF | -2.739 | 0.049 | 2.873 | 0.021 |
| SLC12A5-AS1 | 1.449 | 0.028 | -1.458 | 0.019 |
| TRAF3IP1 | -2.146 | 0.000 | 2.225 | 0.000 |
| GIGYF2 | -1.612 | 0.000 | 1.268 | 0.000 |
| RUFY2 | -2.086 | 0.000 | 2.463 | 0.000 |
| AGAP6 | -1.662 | 0.000 | 1.646 | 0.000 |
| AGAP9 | -2.115 | 0.010 | 2.547 | 0.000 |
| BMS1P1 | -1.212 | 0.009 | 1.249 | 0.001 |
| MACO1 | -1.456 | 0.000 | 1.651 | 0.000 |
| ZDBF2 | -1.805 | 0.000 | 2.313 | 0.000 |
| BMPR2 | -1.559 | 0.000 | 1.540 | 0.000 |
| HSD17B8 | 1.802 | 0.000 | -1.063 | 0.000 |
| HLA-DMA | 1.075 | 0.012 | -1.237 | 0.000 |
| NEU1 | 1.431 | 0.000 | -1.697 | 0.000 |
| MSH5 | -3.086 | 0.000 | 2.931 | 0.000 |
| GPANK1 | 1.235 | 0.000 | -1.281 | 0.000 |
| NFKBIL1 | 1.481 | 0.000 | -1.118 | 0.000 |
| ZNF616 | -1.059 | 0.035 | 1.580 | 0.000 |
| GABBR1 | -1.714 | 0.000 | 2.795 | 0.000 |
| SLFN12L | -4.158 | 0.046 | 4.887 | 0.004 |
| ZBTB10 | -1.067 | 0.000 | 1.356 | 0.000 |
| SAMD9 | -4.027 | 0.000 | 3.351 | 0.000 |
| ITSN1 | -2.578 | 0.000 | 2.065 | 0.000 |
| C1RL-AS1 | -1.553 | 0.001 | 1.252 | 0.002 |
| HERC2P9 | -2.726 | 0.000 | 2.909 | 0.000 |
| HLA-H | 1.022 | 0.015 | -1.054 | 0.005 |
| CFAP44 | -4.533 | 0.000 | 4.331 | 0.000 |
| AC117402.1 | 3.800 | 0.000 | -1.127 | 0.006 |
| VGLL3 | -2.667 | 0.000 | 1.676 | 0.000 |
| SNORD20 | -2.943 | 0.012 | 2.809 | 0.009 |
| MT-TN | 1.348 | 0.001 | -2.059 | 0.000 |
| MT-TC | 1.450 | 0.000 | -1.860 | 0.000 |
| MT-TY | 1.391 | 0.000 | -1.471 | 0.000 |
| DIO2 | -2.655 | 0.000 | 1.486 | 0.000 |
| DENND1B | -1.085 | 0.000 | 1.760 | 0.000 |
| LPAL2 | -1.788 | 0.033 | 1.598 | 0.031 |
| SCAF8 | -1.407 | 0.000 | 1.184 | 0.000 |
| ZNF254 | -1.675 | 0.000 | 2.032 | 0.000 |
| TRIM59 | -1.905 | 0.000 | 1.410 | 0.000 |
| RPL18AP3 | 1.208 | 0.000 | -1.477 | 0.000 |
| ARL2 | 1.087 | 0.000 | -1.047 | 0.000 |
| FIRRE | -1.044 | 0.013 | 1.078 | 0.004 |
| IMPDH1P5 | -3.195 | 0.000 | 1.349 | 0.043 |
| SRA1 | 1.254 | 0.000 | -1.009 | 0.000 |
| RPLP0P6 | 1.009 | 0.000 | -1.116 | 0.000 |
| PIGCP1 | -1.594 | 0.000 | 1.677 | 0.000 |
| DDX47 | -2.844 | 0.000 | 3.070 | 0.000 |
| ZNF888 | -1.356 | 0.001 | 1.161 | 0.001 |
| ZNF845 | -2.746 | 0.000 | 2.553 | 0.000 |
| LTB4R | -1.196 | 0.000 | 1.491 | 0.000 |
| ITGA1 | -2.065 | 0.000 | 2.210 | 0.000 |
| TTLL3 | -1.350 | 0.000 | 1.415 | 0.000 |
| ZNF891 | -1.796 | 0.000 | 1.903 | 0.000 |
| PAXIP1-AS2 | -1.288 | 0.010 | 1.620 | 0.000 |
| PLEKHM1P1 | -2.376 | 0.000 | 1.969 | 0.000 |
| ANG | 1.359 | 0.009 | -1.311 | 0.004 |
| HAUS3 | -1.604 | 0.000 | 1.653 | 0.000 |
| LRRC37A4P | -2.398 | 0.000 | 2.739 | 0.000 |
| EML6 | -1.124 | 0.000 | 1.526 | 0.000 |
| AC092821.1 | -3.474 | 0.004 | 3.808 | 0.000 |
| DDX12P | -1.385 | 0.000 | 1.296 | 0.000 |
| PPIAP29 | 1.452 | 0.000 | -1.111 | 0.000 |
| TTC3P1 | -2.257 | 0.001 | 3.674 | 0.000 |
| BX322639.1 | -1.843 | 0.008 | 2.333 | 0.000 |
| AC138409.2 | -1.294 | 0.016 | 1.027 | 0.031 |
| GOLGA8B | -3.734 | 0.000 | 3.948 | 0.000 |
| MIR17HG | -3.315 | 0.000 | 3.533 | 0.000 |
| ZNF407 | -2.188 | 0.000 | 1.361 | 0.000 |
| RPS2P55 | 1.122 | 0.044 | -1.099 | 0.024 |
| AL390719.1 | 2.206 | 0.000 | -1.465 | 0.000 |
| AC016739.1 | 1.046 | 0.000 | -1.307 | 0.000 |
| TATDN2P2 | -1.703 | 0.000 | 1.561 | 0.000 |
| AL591135.1 | -2.479 | 0.000 | 1.763 | 0.003 |
| FTH1P8 | 1.055 | 0.000 | -1.083 | 0.000 |
| HIST2H2BD | -1.706 | 0.005 | 1.844 | 0.000 |
| SNORD100 | -3.963 | 0.001 | 3.329 | 0.012 |
| GAS8-AS1 | -3.821 | 0.017 | 3.608 | 0.020 |
| TRIM16 | 1.367 | 0.000 | -1.082 | 0.000 |
| CCNL2 | -1.907 | 0.000 | 1.698 | 0.000 |
| TCAF1P1 | -1.768 | 0.043 | 2.698 | 0.001 |
| MIR503HG | -1.897 | 0.006 | 1.917 | 0.002 |
| ENTPD3-AS1 | 1.245 | 0.011 | -1.134 | 0.007 |
| AFG3L1P | -1.523 | 0.000 | 1.463 | 0.000 |
| RPL26P28 | -1.607 | 0.001 | 2.034 | 0.000 |
| EPB41L4A-AS1 | 1.003 | 0.000 | -1.056 | 0.000 |
| AL109613.1 | -4.610 | 0.022 | 4.655 | 0.006 |
| AC000123.1 | -3.275 | 0.021 | 3.235 | 0.017 |
| RPL12P14 | 1.410 | 0.000 | -1.209 | 0.000 |
| SMIM25 | 2.562 | 0.001 | -1.942 | 0.007 |
| HSP90AA2P | -3.531 | 0.000 | 3.410 | 0.000 |
| SH3BP5-AS1 | -3.553 | 0.000 | 3.215 | 0.000 |
| SNX18P3 | -1.762 | 0.031 | 2.220 | 0.001 |
| AC069282.1 | -1.661 | 0.023 | 1.520 | 0.027 |
| DNAJC19P5 | -3.467 | 0.010 | 2.899 | 0.040 |
| FAM229A | -1.937 | 0.001 | 1.783 | 0.001 |
| ERCC6 | -1.216 | 0.000 | 1.129 | 0.000 |
| AHCTF1P1 | -3.369 | 0.000 | 3.486 | 0.000 |
| LINC-PINT | -3.420 | 0.017 | 3.989 | 0.001 |
| CYP4F26P | -1.247 | 0.018 | 2.129 | 0.000 |
| ZNF451-AS1 | -2.510 | 0.043 | 2.454 | 0.027 |
| SUGT1P1 | 1.095 | 0.007 | -1.006 | 0.005 |
| RPS28P7 | 1.123 | 0.000 | -1.373 | 0.000 |
| PARG | -1.393 | 0.000 | 1.160 | 0.000 |
| STK4-AS1 | 2.635 | 0.000 | -1.416 | 0.007 |
| AL390728.4 | -2.244 | 0.000 | 2.162 | 0.000 |
| AC138969.1 | -2.800 | 0.000 | 2.494 | 0.000 |
| AL365277.1 | -2.988 | 0.004 | 3.532 | 0.000 |
| AC003991.1 | -4.970 | 0.000 | 3.223 | 0.008 |
| AC093495.1 | -2.499 | 0.003 | 2.279 | 0.003 |
| AL669831.1 | -2.048 | 0.001 | 2.214 | 0.000 |
| CCT6P1 | -1.171 | 0.001 | 1.149 | 0.000 |
| RAB11FIP1P1 | -4.733 | 0.010 | 5.260 | 0.001 |
| MIR34AHG | -1.351 | 0.046 | 2.064 | 0.000 |
| CALM2P2 | -2.844 | 0.000 | 2.673 | 0.000 |
| ANKRD10-IT1 | -4.703 | 0.001 | 4.409 | 0.000 |
| AC018638.1 | -2.298 | 0.043 | 3.054 | 0.000 |
| RALGAPA1P1 | -1.387 | 0.005 | 1.900 | 0.000 |
| MBNL1-AS1 | -2.106 | 0.000 | 1.623 | 0.000 |
| ITGB1-DT | 2.116 | 0.000 | -1.124 | 0.020 |
| SOS1-IT1 | -2.703 | 0.002 | 3.103 | 0.000 |
| LINC01315 | 2.020 | 0.000 | -1.176 | 0.000 |
| ALMS1-IT1 | -2.549 | 0.034 | 2.952 | 0.002 |
| PHBP9 | -4.411 | 0.000 | 3.817 | 0.001 |
| ANKRD18B | -2.231 | 0.000 | 2.767 | 0.000 |
| AC021078.1 | -2.077 | 0.000 | 1.861 | 0.000 |
| FAM133DP | -2.848 | 0.000 | 2.570 | 0.000 |
| FTX | -3.178 | 0.000 | 2.801 | 0.000 |
| AC018638.2 | -1.754 | 0.000 | 1.857 | 0.000 |
| YY2 | -2.776 | 0.000 | 2.458 | 0.000 |
| AGAP10P | -2.687 | 0.001 | 2.132 | 0.014 |
| AC079250.1 | 1.357 | 0.002 | -1.968 | 0.000 |
| LINC00852 | -1.963 | 0.001 | 1.830 | 0.001 |
| FAM225A | 3.249 | 0.000 | -1.180 | 0.000 |
| AL133406.2 | -5.584 | 0.001 | 5.148 | 0.001 |
| Z97180.1 | -1.205 | 0.024 | 2.053 | 0.000 |
| PIK3CD-AS2 | 1.055 | 0.031 | -1.198 | 0.005 |
| ZBED9 | -1.892 | 0.007 | 1.690 | 0.013 |
| SLC16A6P1 | -4.249 | 0.035 | 4.280 | 0.016 |
| KANTR | -1.401 | 0.001 | 1.260 | 0.001 |
| LINC00342 | -4.352 | 0.000 | 4.763 | 0.000 |
| MIR3936HG | -1.159 | 0.001 | 1.253 | 0.000 |
| AC133785.1 | 1.912 | 0.004 | -1.937 | 0.001 |
| LINC00472 | -3.039 | 0.000 | 2.346 | 0.000 |
| GPX1 | 1.001 | 0.000 | -1.182 | 0.000 |
| TMEM238 | 1.094 | 0.000 | -1.261 | 0.000 |
| AC007969.1 | 1.002 | 0.000 | -1.312 | 0.000 |
| YEATS2-AS1 | -2.688 | 0.045 | 2.856 | 0.010 |
| AC138951.1 | -4.253 | 0.046 | 4.459 | 0.010 |
| AC013476.1 | -2.456 | 0.029 | 2.380 | 0.023 |
| AC012146.1 | 1.795 | 0.000 | -1.071 | 0.000 |
| ZNF37BP | -3.991 | 0.000 | 4.071 | 0.000 |
| FAM133B | -2.755 | 0.000 | 2.801 | 0.000 |
| SLC25A25-AS1 | -2.438 | 0.000 | 1.946 | 0.000 |
| FTH1P2 | 1.404 | 0.000 | -1.551 | 0.000 |
| SEMA3F-AS1 | -3.102 | 0.000 | 3.079 | 0.000 |
| ID2-AS1 | -2.433 | 0.039 | 2.598 | 0.012 |
| AL356599.1 | -1.693 | 0.035 | 1.799 | 0.006 |
| AC009299.3 | -3.703 | 0.033 | 3.800 | 0.011 |
| OLMALINC | 1.763 | 0.000 | -1.227 | 0.000 |
| AC006978.1 | -2.631 | 0.000 | 2.109 | 0.000 |
| NEXN-AS1 | -3.649 | 0.001 | 4.429 | 0.000 |
| PNMA6A | 3.364 | 0.009 | -1.860 | 0.043 |
| ASMTL-AS1 | -2.404 | 0.015 | 2.661 | 0.001 |
| ZBED5 | -1.110 | 0.000 | 1.113 | 0.000 |
| MIR600HG | -1.055 | 0.018 | 1.010 | 0.009 |
| FTH1P11 | 1.952 | 0.017 | -1.614 | 0.029 |
| TTN-AS1 | -1.322 | 0.050 | 1.333 | 0.017 |
| Z95152.1 | -4.461 | 0.000 | 3.477 | 0.010 |
| RPL35P1 | 1.233 | 0.019 | -1.298 | 0.004 |
| LRRC37A2 | -1.984 | 0.000 | 1.614 | 0.000 |
| GOLGA2P5 | -3.151 | 0.000 | 2.969 | 0.000 |
| PAXBP1-AS1 | -2.394 | 0.019 | 2.410 | 0.009 |
| AC108058.1 | -2.289 | 0.000 | 1.637 | 0.005 |
| RN7SL535P | -3.859 | 0.017 | 3.411 | 0.035 |
| PSMD6-AS2 | -3.712 | 0.005 | 3.992 | 0.001 |
| AL157392.3 | -1.808 | 0.007 | 1.853 | 0.001 |
| PARGP1 | -1.221 | 0.021 | 1.067 | 0.030 |
| PSMB9 | 1.559 | 0.000 | -1.228 | 0.000 |
| AL450384.2 | -3.102 | 0.002 | 3.049 | 0.001 |
| RPS2P5 | 1.047 | 0.000 | -1.160 | 0.000 |
| CDKN2B-AS1 | -1.413 | 0.017 | 1.608 | 0.001 |
| PLCXD2 | -1.193 | 0.001 | 1.644 | 0.000 |
| SUCLG2-AS1 | -1.826 | 0.001 | 1.598 | 0.001 |
| RPL36A | -1.638 | 0.000 | 1.938 | 0.000 |
| ACTG1P20 | -2.666 | 0.016 | 2.357 | 0.036 |
| AC069499.1 | -4.502 | 0.000 | 4.051 | 0.000 |
| MUC20-OT1 | -2.023 | 0.000 | 1.387 | 0.000 |
| MTFP1 | 1.291 | 0.008 | -1.148 | 0.009 |
| EIF6 | 1.301 | 0.000 | -1.160 | 0.000 |
| AC108010.1 | -1.384 | 0.015 | 1.729 | 0.000 |
| CNTF | -2.487 | 0.000 | 2.114 | 0.001 |
| AC018638.4 | -1.251 | 0.001 | 1.338 | 0.000 |
| C4orf48 | 1.207 | 0.000 | -1.371 | 0.000 |
| AC018638.5 | -1.313 | 0.004 | 1.708 | 0.000 |
| NPIPB5 | -2.132 | 0.000 | 1.989 | 0.000 |
| FLNB-AS1 | -2.828 | 0.009 | 2.973 | 0.001 |
| TMEM141 | 1.296 | 0.000 | -1.245 | 0.000 |
| ODCP | -3.504 | 0.038 | 3.603 | 0.021 |
| GABPB1-AS1 | -1.057 | 0.000 | 1.864 | 0.000 |
| RNF139-AS1 | -1.703 | 0.038 | 2.045 | 0.001 |
| NEAT1 | -2.537 | 0.000 | 2.453 | 0.000 |
| ZNF585B | -1.326 | 0.006 | 1.535 | 0.000 |
| NADK2-AS1 | -3.351 | 0.011 | 4.232 | 0.000 |
| AP003352.1 | -2.261 | 0.000 | 1.979 | 0.000 |
| AL049840.1 | -2.920 | 0.001 | 2.604 | 0.003 |
| H2AFJ | 1.015 | 0.000 | -1.158 | 0.000 |
| STARD4-AS1 | -3.407 | 0.000 | 3.107 | 0.000 |
| UBAP1L | -3.280 | 0.001 | 3.522 | 0.000 |
| AC091948.1 | -4.260 | 0.000 | 3.109 | 0.021 |
| MARCOL | -2.312 | 0.000 | 1.724 | 0.000 |
| RBM14-RBM4 | -2.815 | 0.040 | 2.657 | 0.037 |
| CASC9 | -1.858 | 0.000 | 2.071 | 0.000 |
| YJEFN3 | -2.808 | 0.002 | 3.092 | 0.000 |
| AP002884.1 | 4.017 | 0.000 | -1.237 | 0.014 |
| AC122718.1 | -2.602 | 0.000 | 2.360 | 0.000 |
| AC034213.1 | 6.370 | 0.000 | -1.827 | 0.000 |
| RPL32P3 | -1.445 | 0.004 | 1.285 | 0.001 |
| MALAT1 | -1.590 | 0.021 | 2.566 | 0.000 |
| FAM160A1-DT | -1.973 | 0.014 | 2.078 | 0.003 |
| AP001574.1 | -2.864 | 0.008 | 2.670 | 0.009 |
| AC107959.3 | 3.589 | 0.000 | -1.814 | 0.000 |
| AC022893.1 | -4.137 | 0.006 | 4.057 | 0.007 |
| PRKDC | -1.242 | 0.000 | 1.069 | 0.000 |
| OTUD6B-AS1 | -1.839 | 0.000 | 1.915 | 0.000 |
| UTP14C | -1.309 | 0.001 | 1.137 | 0.002 |
| ZNF260 | -1.766 | 0.000 | 2.268 | 0.000 |
| AC113191.1 | -1.012 | 0.002 | 1.054 | 0.000 |
| AP003392.1 | -2.571 | 0.013 | 2.460 | 0.007 |
| AC084337.1 | -3.215 | 0.001 | 2.903 | 0.001 |
| SMG1P6 | -5.926 | 0.000 | 4.936 | 0.002 |
| ANKHD1-EIF4EBP3 | -2.730 | 0.000 | 2.729 | 0.000 |
| AC069185.1 | 3.446 | 0.000 | -1.948 | 0.001 |
| EID3 | -1.609 | 0.000 | 1.329 | 0.000 |
| EBLN2 | -4.281 | 0.033 | 4.933 | 0.002 |
| AP003486.1 | -1.216 | 0.028 | 2.145 | 0.000 |
| ZNF432 | -1.911 | 0.000 | 1.988 | 0.000 |
| ZNF10 | -1.575 | 0.000 | 1.669 | 0.000 |
| ZNF350 | -1.157 | 0.010 | 1.472 | 0.000 |
| ZNF253 | -2.319 | 0.000 | 2.319 | 0.000 |
| PWAR6 | -2.547 | 0.000 | 2.739 | 0.000 |
| AC008147.2 | -3.892 | 0.018 | 4.024 | 0.007 |
| ZBED6 | -2.381 | 0.041 | 2.486 | 0.009 |
| AC048341.1 | -2.256 | 0.000 | 1.982 | 0.000 |
| AC004241.1 | 2.374 | 0.001 | -1.363 | 0.029 |
| AC010203.1 | -3.317 | 0.002 | 3.312 | 0.001 |
| AC121761.1 | -1.566 | 0.002 | 1.790 | 0.000 |
| AC078778.1 | -3.485 | 0.003 | 3.041 | 0.011 |
| AC073611.1 | 1.290 | 0.010 | -1.545 | 0.001 |
| PSMA3-AS1 | -1.855 | 0.000 | 1.805 | 0.000 |
| KRT7-AS | -2.581 | 0.000 | 2.814 | 0.000 |
| AC025034.1 | -3.640 | 0.000 | 3.583 | 0.000 |
| LINC00641 | -2.151 | 0.000 | 2.625 | 0.000 |
| CEP95 | -2.255 | 0.000 | 2.214 | 0.000 |
| HNRNPCP1 | -2.663 | 0.024 | 2.690 | 0.010 |
| AC116158.1 | -5.017 | 0.004 | 4.243 | 0.019 |
| ISCA1P4 | 5.162 | 0.000 | -1.898 | 0.003 |
| UBE2Q2P2 | -1.686 | 0.008 | 1.932 | 0.000 |
| LINC00052 | -2.637 | 0.000 | 1.382 | 0.011 |
| HSP90B2P | -4.393 | 0.020 | 4.416 | 0.012 |
| AC083843.2 | -3.107 | 0.000 | 2.567 | 0.004 |
| AL390728.6 | -2.044 | 0.002 | 2.007 | 0.002 |
| AC107375.1 | -1.999 | 0.041 | 2.038 | 0.018 |
| AC107068.1 | -1.623 | 0.001 | 1.693 | 0.000 |
| AC009120.2 | -3.006 | 0.002 | 3.482 | 0.000 |
| TGFBR3L | 1.351 | 0.000 | -1.120 | 0.000 |
| AC124944.3 | -1.727 | 0.007 | 2.082 | 0.000 |
| KCNJ18 | 2.337 | 0.000 | -2.296 | 0.000 |
| AC132872.1 | -1.932 | 0.003 | 1.539 | 0.020 |
| AC110597.1 | -3.941 | 0.000 | 3.338 | 0.003 |
| SNAI3-AS1 | 2.026 | 0.000 | -1.070 | 0.007 |
| LINC01963 | -1.927 | 0.000 | 1.356 | 0.009 |
| AC004656.1 | -1.354 | 0.000 | 1.221 | 0.000 |
| FBXL19-AS1 | -1.219 | 0.000 | 1.521 | 0.000 |
| CCPG1 | -1.795 | 0.000 | 2.324 | 0.000 |
| AP001486.2 | -3.399 | 0.000 | 2.893 | 0.000 |
| AP000766.1 | -3.149 | 0.031 | 3.196 | 0.019 |
| AC107027.3 | -1.923 | 0.001 | 2.056 | 0.000 |
| AC012181.2 | 1.826 | 0.005 | -1.048 | 0.036 |
| AC010168.2 | -2.583 | 0.000 | 2.341 | 0.000 |
| LINC01355 | -2.866 | 0.033 | 3.006 | 0.013 |
| AC097461.1 | -1.060 | 0.020 | 1.016 | 0.015 |
| AC005674.2 | -4.924 | 0.004 | 4.766 | 0.004 |
| SMG1P7 | -2.259 | 0.000 | 2.792 | 0.000 |
| HIST2H2BC | -1.101 | 0.001 | 1.050 | 0.000 |
| AL136537.2 | -1.511 | 0.036 | 2.089 | 0.000 |
| AC007406.5 | -1.653 | 0.000 | 1.638 | 0.000 |
| AC090826.1 | -2.685 | 0.002 | 2.501 | 0.003 |
| CORO7 | 1.138 | 0.001 | -1.086 | 0.001 |
| AC087741.1 | -2.439 | 0.000 | 2.335 | 0.000 |
| TMPOP2 | -2.605 | 0.003 | 2.961 | 0.000 |
| AC004148.2 | -2.533 | 0.000 | 2.526 | 0.000 |
| AC112907.3 | -2.188 | 0.001 | 2.466 | 0.000 |
| AP005899.1 | -3.945 | 0.003 | 3.128 | 0.025 |
| NBPF11 | -1.828 | 0.000 | 2.289 | 0.000 |
| AC015813.1 | -4.275 | 0.000 | 4.239 | 0.000 |
| MIR3153 | -4.536 | 0.020 | 4.640 | 0.008 |
| AL359922.2 | -4.362 | 0.000 | 4.288 | 0.000 |
| AC005256.1 | 1.111 | 0.002 | -1.195 | 0.000 |
| AC060766.1 | -2.426 | 0.006 | 2.402 | 0.002 |
| LINC02081 | 1.223 | 0.000 | -1.680 | 0.000 |
| AC020934.1 | 1.692 | 0.039 | -2.111 | 0.007 |
| ZNF224 | -1.622 | 0.002 | 1.452 | 0.001 |
| AC060766.7 | -2.561 | 0.037 | 2.541 | 0.019 |
| AC021594.2 | -3.220 | 0.026 | 3.123 | 0.023 |
| AC005261.1 | -1.425 | 0.000 | 1.458 | 0.000 |
| AC007842.1 | -3.889 | 0.014 | 3.338 | 0.042 |
| MAGIX | 1.265 | 0.024 | -1.196 | 0.022 |
| IKBKG | 1.500 | 0.002 | -1.448 | 0.010 |
| AC008982.2 | -3.186 | 0.000 | 2.636 | 0.001 |
| KCNQ1OT1 | -5.906 | 0.000 | 6.341 | 0.000 |
| AC022150.4 | -2.622 | 0.000 | 2.452 | 0.000 |
| EGLN2 | -2.916 | 0.000 | 2.586 | 0.001 |
| SNHG8 | 1.484 | 0.000 | -1.238 | 0.000 |
| AL606834.1 | -1.739 | 0.000 | 1.095 | 0.002 |
| AC010969.2 | -2.619 | 0.000 | 2.141 | 0.000 |
| AC087481.3 | -1.660 | 0.000 | 1.503 | 0.000 |
| AL606834.2 | -4.539 | 0.016 | 4.694 | 0.007 |
| MIR222HG | -3.516 | 0.000 | 4.090 | 0.000 |
| YTHDF3-AS1 | 2.461 | 0.001 | -1.325 | 0.031 |
| AC095055.1 | -1.302 | 0.019 | 1.091 | 0.030 |
| GAS2L2 | 2.315 | 0.024 | -2.445 | 0.008 |
| AC015849.3 | -2.668 | 0.023 | 2.376 | 0.032 |
| RASL10B | 1.853 | 0.000 | -1.088 | 0.008 |
| AL049844.2 | -4.395 | 0.025 | 4.746 | 0.005 |
| NBPF19 | -1.131 | 0.000 | 1.345 | 0.000 |
| NBPF10 | -1.557 | 0.016 | 2.182 | 0.000 |
| Z83843.1 | -3.528 | 0.001 | 3.510 | 0.000 |
| AC104118.1 | -3.048 | 0.001 | 2.809 | 0.002 |
| AC024060.1 | -2.941 | 0.000 | 2.408 | 0.003 |
| AL603756.1 | -1.672 | 0.041 | 2.390 | 0.000 |
| AL021368.2 | -2.137 | 0.012 | 2.098 | 0.007 |
| AC093297.2 | -1.754 | 0.000 | 1.414 | 0.000 |
| AL031775.2 | -4.731 | 0.011 | 4.935 | 0.003 |
| GTF2IP13 | -1.350 | 0.005 | 1.495 | 0.000 |
| AC067750.1 | -2.118 | 0.000 | 1.694 | 0.005 |
| GTF2IP20 | -1.981 | 0.000 | 2.064 | 0.000 |
| AL021707.6 | -2.816 | 0.049 | 3.020 | 0.015 |
| AL592148.3 | -2.403 | 0.006 | 2.025 | 0.025 |
| AC018645.2 | -1.876 | 0.005 | 1.690 | 0.006 |
| LINC02035 | -1.371 | 0.000 | 1.787 | 0.000 |
| AL365203.2 | -2.735 | 0.007 | 3.061 | 0.000 |
| AC005046.1 | -5.575 | 0.001 | 4.714 | 0.005 |
| AC144652.1 | 2.027 | 0.000 | -1.754 | 0.000 |
| NBPF26 | -1.141 | 0.006 | 1.593 | 0.000 |
| AC097376.2 | -2.362 | 0.000 | 2.360 | 0.000 |
| AC016717.2 | -2.592 | 0.000 | 1.205 | 0.001 |
| AL355488.1 | -5.303 | 0.003 | 5.110 | 0.002 |
| AC245041.1 | -3.713 | 0.000 | 2.913 | 0.000 |
| TBC1D3D | -2.909 | 0.013 | 3.251 | 0.000 |
| AC079684.1 | -3.479 | 0.001 | 3.818 | 0.000 |
| AC241952.1 | -3.603 | 0.000 | 2.791 | 0.000 |
| AL162731.1 | 2.615 | 0.032 | -2.096 | 0.045 |
| AL136981.2 | -5.076 | 0.003 | 4.517 | 0.012 |
| AC092747.4 | -2.830 | 0.000 | 2.527 | 0.000 |
| TUBGCP5 | -1.067 | 0.000 | 1.010 | 0.000 |
| WHAMMP3 | -1.751 | 0.004 | 2.208 | 0.000 |
| AC004556.1 | 1.009 | 0.000 | -1.375 | 0.000 |
| AL133243.3 | -3.357 | 0.016 | 3.345 | 0.015 |
| HERC2P2 | -3.188 | 0.000 | 2.806 | 0.000 |
| AL161891.1 | -2.010 | 0.000 | 1.455 | 0.000 |
| AC245041.2 | -2.400 | 0.000 | 1.498 | 0.000 |
| TYW1B | -1.874 | 0.021 | 1.811 | 0.014 |
| AC005332.5 | -1.760 | 0.031 | 2.126 | 0.001 |
| AP003900.1 | -3.403 | 0.001 | 2.946 | 0.003 |
| AC139100.2 | -3.946 | 0.012 | 3.793 | 0.013 |
| DDX52 | -1.223 | 0.000 | 1.047 | 0.000 |
| ZNF8 | -1.582 | 0.000 | 1.050 | 0.000 |
| GGNBP2 | -1.236 | 0.000 | 1.007 | 0.000 |
| AL031673.1 | -2.717 | 0.028 | 2.873 | 0.007 |
| AL354822.1 | -1.952 | 0.016 | 1.628 | 0.042 |
| GOLGA6L10 | -2.174 | 0.038 | 2.390 | 0.005 |
| AC007325.4 | 1.450 | 0.005 | -1.152 | 0.011 |
| CU633906.2 | -1.604 | 0.034 | 2.286 | 0.000 |
| HEIH | 2.146 | 0.000 | -1.087 | 0.000 |
| AP001148.1 | -4.055 | 0.001 | 3.631 | 0.003 |
| AC090181.3 | -4.309 | 0.001 | 4.311 | 0.000 |
| AC022400.7 | -1.567 | 0.000 | 1.422 | 0.000 |
| AC005839.1 | -1.850 | 0.009 | 1.461 | 0.047 |
| AC018628.1 | -2.792 | 0.000 | 2.355 | 0.003 |
| AC003681.1 | -4.641 | 0.001 | 4.523 | 0.001 |
| PWAR5 | -2.552 | 0.000 | 2.938 | 0.000 |
| AC122688.3 | -1.630 | 0.009 | 1.802 | 0.001 |
| AL136164.4 | -5.986 | 0.000 | 4.729 | 0.004 |
| AP000866.6 | -2.754 | 0.018 | 2.861 | 0.007 |
| AC021945.1 | -4.469 | 0.002 | 3.667 | 0.019 |
| AC007191.1 | -1.582 | 0.004 | 1.540 | 0.001 |
| AC112497.1 | -2.887 | 0.000 | 2.602 | 0.000 |
| AC007382.1 | -2.759 | 0.000 | 2.337 | 0.000 |
| AC020978.9 | -3.002 | 0.027 | 3.255 | 0.003 |
| AC069547.1 | -2.372 | 0.004 | 2.272 | 0.003 |
| AP001350.2 | -3.594 | 0.002 | 2.783 | 0.028 |
| AC012676.5 | -3.217 | 0.001 | 3.149 | 0.000 |
| AC126474.2 | -2.111 | 0.000 | 1.327 | 0.004 |
| PLAC4 | -2.649 | 0.003 | 3.306 | 0.000 |
| AC073857.1 | -1.896 | 0.000 | 1.549 | 0.000 |
| AL662795.2 | -1.931 | 0.024 | 3.292 | 0.000 |
| AC027290.2 | -2.154 | 0.000 | 2.192 | 0.000 |
| AC011498.7 | -2.065 | 0.005 | 1.734 | 0.014 |
| AC000123.3 | -3.571 | 0.000 | 3.593 | 0.000 |
| AP000648.3 | -3.233 | 0.000 | 2.822 | 0.000 |
| LINC00294 | -1.622 | 0.000 | 1.253 | 0.000 |
| NPTN-IT1 | -2.672 | 0.000 | 2.794 | 0.000 |
| SLFNL1-AS1 | -2.541 | 0.000 | 2.779 | 0.000 |
| HELLPAR | -3.473 | 0.000 | 2.803 | 0.000 |
| CICP14 | -1.773 | 0.000 | 1.582 | 0.000 |
| AC233280.19 | -4.322 | 0.035 | 4.105 | 0.035 |
| FRG1CP | -1.202 | 0.000 | 1.097 | 0.000 |
| LBHD2 | 1.665 | 0.013 | -1.126 | 0.047 |
| AP002851.1 | 1.872 | 0.000 | -1.254 | 0.001 |
| EEF1AKMT4 | 1.309 | 0.000 | -1.048 | 0.000 |
| AC091057.6 | -2.724 | 0.000 | 2.001 | 0.001 |
| AC068831.7 | -2.432 | 0.000 | 1.815 | 0.000 |
| AL591485.1 | 1.492 | 0.048 | -1.207 | 0.048 |
| TFAP2A-AS2 | -1.842 | 0.004 | 1.902 | 0.001 |
| AP001362.2 | 1.348 | 0.016 | -1.392 | 0.005 |
| AC010422.8 | -3.195 | 0.003 | 2.551 | 0.029 |
| AL162458.1 | -1.263 | 0.038 | 1.283 | 0.025 |
| AC009090.6 | -3.924 | 0.000 | 3.789 | 0.000 |

**4 Figure**


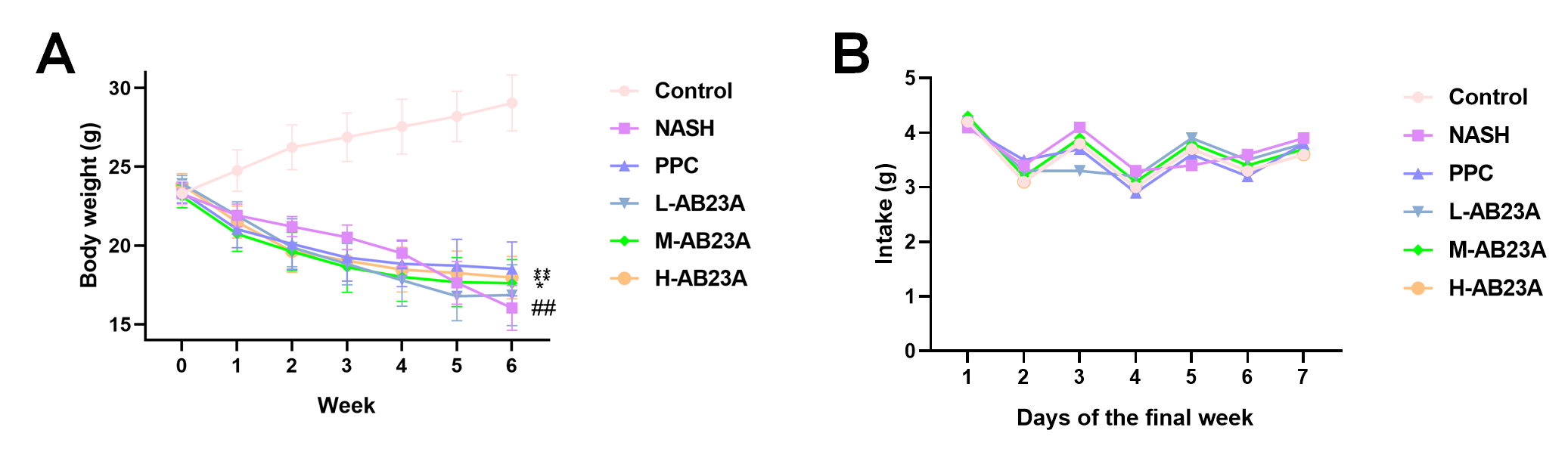


**Figure S1** The effect of AB23A on the body weight (A) and food intake (B) of NASH mice. Data are presented as the mean ± SD. n = 10 per group. ##*P* < 0.01 compared to the Control group; **P* < 0.05, ***P* < 0.01 compared to the NASH group.
